# Distributed Hypothesis Testing over a Noisy Channel: Error-Exponents Trade-Off

**DOI:** 10.3390/e25020304

**Published:** 2023-02-06

**Authors:** Sreejith Sreekumar, Deniz Gündüz

**Affiliations:** 1 Department of Electrical and Computer Engineering, Cornell University, Ithaca, NY 14850, USA; 2 Department of Electrical and Electronic Engineering, Imperial College London, London SW72AZ, UK

**Keywords:** distributed hypothesis testing, noisy channel, error-exponents, source-channel separation, joint source-channel coding, hybrid coding

## Abstract

A two-terminal distributed binary hypothesis testing problem over a noisy channel is studied. The two terminals, called the observer and the decision maker, each has access to *n* independent and identically distributed samples, denoted by U and V, respectively. The observer communicates to the decision maker over a discrete memoryless channel, and the decision maker performs a binary hypothesis test on the joint probability distribution of (U,V) based on V and the noisy information received from the observer. The trade-off between the exponents of the type I and type II error probabilities is investigated. Two inner bounds are obtained, one using a separation-based scheme that involves type-based compression and unequal error-protection channel coding, and the other using a joint scheme that incorporates type-based hybrid coding. The separation-based scheme is shown to recover the inner bound obtained by Han and Kobayashi for the special case of a rate-limited noiseless channel, and also the one obtained by the authors previously for a corner point of the trade-off. Finally, we show via an example that the joint scheme achieves a strictly tighter bound than the separation-based scheme for some points of the error-exponents trade-off.

## 1. Introduction

Hypothesis testing (HT), which refers to the problem of choosing between one or more alternatives based on available data, plays a central role in statistics and information theory. Distributed HT (DHT) problems arise in situations where the test data are scattered across multiple terminals, and need to be communicated to a central terminal, called the *decision maker*, which performs the hypothesis test. The need to jointly optimize the communication scheme and the hypothesis test makes DHT problems much more challenging than their centralized counterparts. Indeed, while an efficient characterization of the optimal hypothesis test and its asymptotic performance is well known in the centralized setting, thanks to [1,2,3,4,5], the same problem in even the simplest distributed setting remains open, except for some special cases (see [6,7,8,9,10,11]).

In this work, we consider a DHT problem with two parties, an *observer* and a decision maker, such that the former communicates to the latter over a noisy channel. The observer and the decision maker each has access to independent and identically distributed samples, denoted by U and V, respectively. Based on the information received from the observer and its own observations V, the decision maker performs a binary hypothesis test on the joint distribution of (U,V). Our goal is to characterize the trade-off between the best achievable rate of decay (or exponent) of the type I and type II error probabilities with respect to the sample size. We will refer to this problem as *DHT over a noisy channel*, and its special instance with the noisy channel replaced by a rate-limited noiseless channel as *DHT over a noiseless channel*.

### 1.1. Background

Distributed statistical inference problems were first conceived in [12] and the information-theoretic study of DHT over a noiseless channel was first investigated in [6], where the objective is to characterize Stein’s exponent $\kappa_{\mathrm{se}}\left(\epsilon \right)$, i.e., the optimal *type II error-exponent* subject to the type I error probability constrained to be at most ϵ∈(0,1). The authors therein established a multi-letter characterization of this quantity including a strong converse, which shows that $\kappa_{\mathrm{se}}\left(\epsilon \right)$ is independent of ϵ. Furthermore, a single-letter characterization of $\kappa_{\mathrm{se}}\left(\epsilon \right)$ is obtained for a special case of HT known as *testing against independence* (TAI), in which the joint distribution factors as a product of the marginal distributions under the alternative hypothesis. Improved lower bounds on $\kappa_{\mathrm{se}}\left(\epsilon \right)$ were subsequently obtained in [7,8], respectively, and the strong converse was extended to zero-rate settings [13]. While all the aforementioned works focus on $\kappa_{\mathrm{se}}\left(\epsilon \right)$, the trade-off between the exponents of both the type I and type II error probabilities in the same setting was first explored in [14].

In the recent years, there has been a renewed interest in distributed statistical inference problems motivated by emerging machine learning applications to be served at the wireless edge, particularly in the context of semantic communications in 5G/6G communication systems [15,16]. Several extensions of the DHT over a noiseless channel problem have been studied, such as generalizations to multi-terminal settings [9,17,18,19,20,21], DHT under security or privacy constraints [22,23,24,25], DHT with lossy compression [26], interactive settings [27,28], successive refinement models [29], and more. Improved bounds have been obtained on the type I and type II error-exponents region [30,31], and on $\kappa_{\mathrm{se}}\left(\epsilon \right)$ for testing correlation between bivariate standard normal distributions [32]. In the simpler zero-rate communication setting, there has been some progress in terms of second-order optimal schemes [33], geometric interpretation of type I and type II error-exponent region [34], and characterization of $\kappa_{\mathrm{se}}\left(\epsilon \right)$ for sequential HT [35]. DHT over noisy communication channels with the goal of characterizing $\kappa_{\mathrm{se}}\left(\epsilon \right)$ has been considered in [10,11,36,37].

### 1.2. Contributions

In this work, our objective is to explore the trade-off between the type I and type II error-exponents for DHT over a noisy channel. This problem is a generalization of [14] from noiseless rate-limited channels to noisy channels, and also of [10,11] from a type I error probability constraint to a positive type I error-exponent constraint.

Our main contributions can be summarized as follows:

(i) We obtain an inner bound (Theorem 1) on the error-exponents trade-off by using a *separate HT and channel coding* scheme (SHTCC) that is a combination of a type-based (type here refers to the empirical probability distribution of a sequence, see [38]) quantize-bin strategy and unequal error-protection scheme of [39]. This result is shown to recover the bounds established in [10,14]. Furthermore, we evaluate Theorem 1 for two important instances of DHT, namely TAI and its opposite, i.e., *testing against dependence* (TAD) in which the joint distribution under the null hypothesis factors as a product of marginal distributions.
(ii) We also obtain a second inner bound (Theorem 2) on the error-exponents trade-off by using a *joint HT and channel coding scheme* (JHTCC) based on *hybrid coding* [40]. Subsequently, we show via an example that the JHTCC scheme strictly outperforms the SHTCC scheme for some points on the error-exponent trade-off.

While the above schemes are inspired from those in [10], which have been proposed with the goal of maximizing the type II error-exponent, novel modifications in its design and analysis are required when considering both of the error-exponents. More specifically, the schemes presented here perform separate quantization-binning or hybrid coding on each individual source sequence type at the observer/encoder (as opposed to a typical ball in [10]) with the corresponding reverse operation implemented at the decision-maker/decoder. This necessitates a different analysis to compute the probabilities of the various error events contributing to the overall error-exponents. We finally mention that the DHT problem considered here was recently investigated in [41], where an inner bound on the error-exponents trade-off (Theorem 2 in [41]) is obtained using a combination of a type-based quantization scheme and unequal error protection scheme of [42] with two special messages. A qualitative comparison between Theorem 2 and Theorem 2 in [41] seems to suggest that the JHTCC scheme here uses a stronger decoding rule depending jointly on the source-channel statistics. In comparison, the metric used at the decoder for the scheme in [41] factors as the sum of two metrics, one which depends only on the source statistics, and the other which depends only on the channel statistics. Importantly, this hints that the inner bound achieved by JHTCC scheme is not subsumed by that in [41]. That said, a direct computational comparison appears difficult, as evaluating the latter requires optimization over several parameters as mentioned in the last paragraph of [41].

The remainder of the paper is organized as follows. Section 2 formulates the operational problem along with the required definitions. The main results are presented in Section 3. The proofs are furnished in Section 4. Finally, concluding remarks are given in Section 5.

## 2. Preliminaries

### 2.1. Notation

We use the following notation. All logarithms are with respect to the natural base *e*. N, R, $R_{\geq 0}$, and $\bar{R}$ denotes the set of natural, real, non-negative real and extended real numbers, respectively. For $a,b\in R_{\geq 0}$, [a:b]:={n∈N:a≤n≤b} and [b]:=[1:b]. Calligraphic letters, e.g., X, denote sets, while $X^{c}$ and |X| stands for its complement and cardinality, respectively. For n∈N, $X^{n}$ denotes the *n*-fold Cartesian product of X, and $x^{n}=\left(x_{1},\cdots ,x_{n}\right)$ denotes an element of $X^{n}$. Bold-face letters denote vectors or sequences, e.g., x for $x^{n}$; its length *n* will be clear from the context. For i,j∈N such that i≤j, $x_{i}^{j}:=\left(x_{i},x_{i+1},\cdots ,x_{j}\right)$, the subscript is omitted when i=1. ${𝟙}_{A}$ denotes the indicator of set A. For a real sequence ${\left\{a_{n}\right\}}_{n\in N}$, $a_{n}\overset{\left(n\right)}{\to }b$ stands for $\lim_{n\to \infty }a_{n}=b$, while $a_{n}≳b$ denotes $\lim_{n\to \infty }a_{n}\geq b$. Similar notations apply for other inequalities. O(·), Ω(·) and o(·) denote standard asymptotic notations.

Random variables and their realizations are denoted by uppercase and lowercase letters, respectively, e.g., *X* and *x*. Similar conventions apply for random vectors and their realizations. The set of all probability mass functions (PMFs) on a finite set X is denoted by P(X). The joint PMF of two discrete random variables *X* and *Y* is denoted by $P_{XY}$; the corresponding marginals are $P_{X}$ and $P_{Y}$. The conditional PMF of *X* given *Y* is represented by $P_{X|Y}$. Expressions such as $P_{XY}=P_{X}P_{Y|X}$ are to be understood as pointwise equality, i.e., $P_{XY}\left(x,y\right)=P_{X}\left(x\right)P_{Y|X}\left(y|x\right)$, for all (x,y)∈X×Y. When the joint distribution of a triple (X,Y,Z) factors as $P_{XYZ}=P_{XY}P_{Z|X}$, these variables form a Markov chain X−Y−Z. When *X* and *Y* are statistically independent, we write X⫫Y. If the entries of $X^{n}$ are drawn in an independent and identically distributed manner, i.e., if $P_{X^{n}}\left(x\right)=\prod_{i=1}^{n}P_{X}\left(x_{i}\right)$, $\forall \;x\in X^{n}$, then the PMF $P_{X^{n}}$ is denoted by $P_{X}^{⊗n}$. Similarly, if $P_{Y^{n}|X^{n}}\left(y|x\right)=\prod_{i=1}^{n}P_{Y|X}\left(y_{i}|x_{i}\right)$ for all $\left(x,y\right)\in X^{n}\times Y^{n}$, then we write $P_{Y|X}^{⊗n}$ for $P_{Y^{n}|X^{n}}$. The conditional product PMF given a fixed $x\in X^{n}$ is designated by $P_{Y|X}^{⊗n}\left(\cdot |x\right)$. The probability measure induced by a PMF *P* is denoted by $P_{P}$. The corresponding expectation is designated by $E_{P}$.

The *type* or empirical PMF of a sequence $x\in X^{n}$ is designated by $P_{x}$, i.e., $P_{x}\left(x\right):=\frac{1}{n}\sum_{i=1}^{n}{𝟙}_{\left\{x_{i}=x\right\}}$. The set of *n*-length sequences $x\in X^{n}$ of type $P_{X}$ is $T_{n}\left(P_{X},X^{n}\right):=\left\{x\in X^{n}:P_{x}=P_{X}\right\}$. Whenever the underlying alphabet $X^{n}$ is clear from the context, $T_{n}\left(P_{X},X^{n}\right)$ is simplified to $T_{n}\left(P_{X}\right)$. The set of all possible types of *n*-length sequences $x\in X^{n}$ is $T\left(X^{n}\right):=\left\{P_{X}\in P\left(X\right):|T_{n}\left(P_{X},X^{n}\right)|\geq 1\right\}$. Similar notations are used for larger combinations, e.g., $P_{xy}$, $T_{n}\left(P_{XY},X\times Y\right)$ and $T(X^{n}\times Y^{n})$. For a given $x\in T_{n}\left(P_{X},X^{n}\right)$ and a conditional PMF $P_{Y|X}$, $T_{n}\left(P_{Y|X},x\right):=\left\{y\in Y^{n}:\left(x,y\right)\in T_{n}\left(P_{XY},X^{n}\times Y^{n}\right)\right\}$ stands for the $P_{Y|X}$-conditional type class of x.

For PMFs P,Q∈P(X), the Kullback–Leibler (KL) divergence between *P* and *Q* is $D\left(P||Q\right):=\sum_{x\in X}P\left(x\right)\log\left(P(x)/Q(x)\right)$. The conditional KL divergence between $P_{Y|X}$ and $Q_{Y|X}$ given $P_{X}$ is $D\left(P_{Y|X}\left|\right|Q_{Y|X}|P_{X}\right):=\sum_{x\in X}P_{X}\left(x\right)D\left(P_{Y|X}\left(\cdot |x\right)\left|\right|Q_{Y|X}\left(\cdot |x\right)\right)$. The mutual information and entropy terms are denoted by $I_{P}\left(\cdot \right)$ and $H_{P}\left(\cdot \right)$, respectively, where *P* denotes the PMF of the relevant random variables. When the PMF is clear from the context, the subscript is omitted. For $\left(x,y\right)\in X^{n}\times Y^{n}$, the empirical conditional entropy of y given x is $H_{e}\left(y|x\right):=H_{P}\left(\tilde{Y}|\tilde{X}\right)$, where $P_{\tilde{X}\tilde{Y}}=P_{xy}$. For a given function f:Z→R and a random variable $Z∼P_{Z}$, the log-moment generating function of *Z* with respect to *f* is $\psi_{P_{Z},f}\left(\lambda \right):=\log E_{P_{Z}}\left[e^{\lambda f(Z)}\right]$ whenever the expectation exists. Finally, let

(1) $$\begin{array}{c} \psi_{P_{Z},f}^{*}\left(\theta \right):=\underset{\lambda \in R}{\sup}\theta \lambda -\psi_{P_{Z},f}\left(\lambda \right), \end{array}$$

denote the rate function (see, e.g., Definition 15.5 in [43]).

### 2.2. Problem Formulation

**Figure 1:**
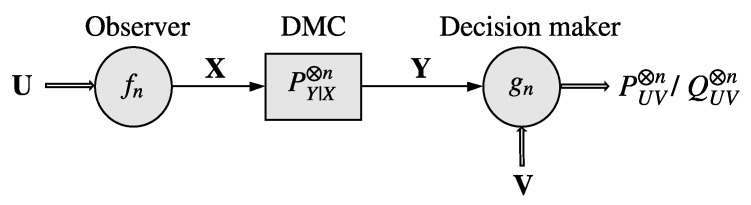
DHT over a noisy channel. The observer observes an *n*-length independent and identically distributed sequence U, and transmits X over the DMC $P_{Y|X}^{⊗n}$. Based on the channel output Y and the *n*-length independent and identically distributed sequence V, the decision maker performs a binary HT to determine whether $\left(U,V\right)∼P_{UV}^{⊗n}$ or $\left(U,V\right)∼Q_{UV}^{⊗n}$.

Let U, V, X and Y be finite sets, and n∈N. The DHT over a noisy channel setting is depicted in Figure 1. Herein, the observer and the decision maker observe *n* independent and identically distributed samples, denoted by u and v, respectively. Based on its observations u, the observer outputs a sequence $x\in X^{n}$ as the channel input sequence (note that the ratio of the number of channel uses to the number of data samples, termed the bandwidth ratio, is taken to be 1 for simplicity; however, our results easily generalize to arbitrary bandwidth ratios). The discrete memoryless channel (DMC) with transition kernel $P_{Y|X}$ produces a sequence $y\in Y^{n}$ according to the probability law $P_{Y|X}^{⊗n}\left(\cdot |x\right)$ as its output. We will assume that $P_{Y|X}\left(\cdot |x\right)\ll P_{Y|X}\left(\cdot |x^{\prime }\right)$, $\forall \;\left(x,x^{\prime }\right)\in X^{2}$, where P≪Q indicates the absolute continuity of *P* with respect to *Q*. Based on its observations, y and v, the decision maker performs binary HT on the joint probability distribution of (U,V) with the null ($H_{0}$) and alternative ($H_{1}$) hypotheses given by

(2a) $$\begin{array}{cc} \; & H_{0}:\;\left(U,V\right)∼P_{UV}^{⊗n}, \end{array}$$

(2b) $$\begin{array}{cc} \; & H_{1}:\;\left(U,V\right)∼Q_{UV}^{⊗n}. \end{array}$$

The decision maker outputs $\hat{h}\in \hat{H}:=\left\{0,1\right\}$ as the decision of the hypothesis test, where 0 and 1 denote $H_{0}$ and $H_{1}$, respectively.

A length-*n* DHT code $c_{n}$ is a pair of functions $\left(f_{n},g_{n}\right)$, where

(i) $f_{n}:U^{n}\to P\left(X^{n}\right)$ denotes the encoding function;
(ii) $g_{n}:V^{n}\times Y^{n}\to \hat{H}$ denotes a deterministic decision function specified by an acceptance region (for null hypothesis $H_{0}$) $A_{n}\subseteq V^{n}\times Y^{n}$ as $g_{n}\left(v,y\right)=1-{𝟙}_{\left\{\left(v,y\right)\in A_{n}\right\}},\;\forall \left(v,y\right)\in V^{n}\times Y^{n}$.

We emphasize at this point that there is no loss in generality in restricting our attention to a deterministic decision function for the objective of characterizing the error-exponents trade-off in HT (for e.g., see Lemma 3 in [24])).

A code $c_{n}=\left(f_{n},g_{n}\right)$ induces the joint PMFs $P_{UVXY\hat{H}}^{\left(c_{n}\right)}$ and $Q_{UVXY\hat{H}}^{\left(c_{n}\right)}$ under the null and alternative hypotheses, respectively, where

(3) $$\begin{array}{c} P_{UVXY\hat{H}}^{\left(c_{n}\right)}\left(u,v,x,y,\hat{h}\right):=P_{UV}^{⊗n}\left(u,v\right)\;f_{n}\left(x|u\right)\;P_{Y|X}^{⊗n}\left(y|x\right)\;{𝟙}_{\left\{g_{n}\left(v,y\right)=\hat{h}\right\}}, \end{array}$$

and

(4) $$\begin{array}{c} Q_{UVXY\hat{H}}^{\left(c_{n}\right)}\left(u,v,x,y,\hat{h}\right):=Q_{UV}^{⊗n}\left(u,v\right)\;f_{n}\left(x|u\right)\;P_{Y|X}^{⊗n}\left(y|x\right)\;{𝟙}_{\left\{g_{n}\left(v,y\right)=\hat{h}\right\}}, \end{array}$$

respectively. For a given code $c_{n}$, the type I and type II error probabilities are $\alpha_{n}\left(c_{n}\right):=P_{P^{\left(c_{n}\right)}}\left(\hat{H}=1\right)$ and $\beta_{n}\left(c_{n}\right):=P_{Q^{\left(c_{n}\right)}}\left(\hat{H}=0\right)$ respectively. The following definition formally states the error-exponents trade-off we aim to characterize.

**Definition 1**  **(Error-exponent region).** *An error-exponent pair $\left(\kappa_{\alpha },\kappa_{\beta }\right)\in R_{\geq 0}^{2}$ is said to be achievable if there exists a sequence of codes ${\left\{c_{n}\right\}}_{n\in N}$ such that*

(5a) $$\underset{n\to \infty }{lim inf}-\frac{1}{n}\log\alpha_{n}\left(c_{n}\right)\geq \kappa_{\alpha },$$

(5b) $$\underset{n\to \infty }{lim inf}-\frac{1}{n}\log\beta_{n}\left(c_{n}\right)\geq \kappa_{\beta }.$$

*The error-exponent region $\bar{R}$ is the closure of the set of all achievable error-exponent pairs $\left(\kappa_{\alpha },\kappa_{\beta }\right)$. Set $R:=\{\left(\kappa_{\alpha },\kappa \left(\kappa_{\alpha }\right)\right):\kappa_{\alpha }\in \left(0,\kappa_{\alpha }^{★}\right)\}$, where $\kappa_{\alpha }^{★}=\inf\left\{\kappa_{\alpha }:\kappa \left(\kappa_{\alpha }\right)=0\right\}$ and $\kappa \left(\kappa_{\alpha }\right):=\sup\left\{\kappa_{\beta }:\left(\kappa_{\alpha },\kappa_{\beta }\right)\in \bar{R}\right\}$.*

We are interested in a computable characterization of R, which pertains to the region of positive error-exponents (i.e., excluding the boundary points corresponding to Stein’s exponent). To this end, we present two inner bounds on R in the next section.

## 3. Main Results

In this section, we obtain two inner bounds on R, first using a separation-based scheme which performs independent HT and channel coding, termed the SHTCC scheme, and the second via a joint HT and channel coding scheme that uses hybrid coding for communication between the observer and the decision maker.

### 3.1. Inner Bound on R via SHTCC Scheme

Let S=X and $P_{SXY}=P_{SX}P_{Y|X}$ be a PMF under which S−X−Y forms a Markov chain. For x∈X, let $\Lambda_{x,P_{SXY}}\left(y\right):=\log\left(P_{Y|X=x}\left(y\right)/P_{Y|S=x}\left(y\right)\right)$ and define

$$\begin{array}{c} E_{\mathrm{sp}}\left(P_{SX},\theta \right):=\sum_{s\in S}P_{S}\left(s\right)\psi_{P_{Y|S=s},\Lambda_{s,P_{SXY}}}^{*}\left(\theta \right), \end{array}$$

where the rate function $\psi^{*}$ is defined in (1). For a fixed $P_{SX}$ and R≥0, let

$$\begin{array}{ccc} E_{\mathrm{ex}}\left(R,P_{SX}\right):=\max_{\rho \geq 1}-\rho R-\rho \log( & \sum_{s,x,\tilde{x}}P_{S}\left(s\right)P_{X|S}\left(x|s\right)P_{X|S}\left(\tilde{x}|s\right)\\ & \;\;\;\;{\left(\sum_{y}{\left(P_{Y|X}\left(y|x\right)P_{Y|X}\left(y|\tilde{x}\right)\right)}^{\frac{1}{2}}\right)}^{\frac{1}{\rho }}), & \end{array}$$

denote the expurgated exponent [38,44]. Let W be a finite set and F denote the set of all continuous mappings from P(U) to P(W|U), where P(W|U) is the set of all conditional distributions $P_{W|U}$. Set $\theta_{l}\left(P_{SX}\right):=\sum_{s\in S}P_{S}\left(s\right)D\left(P_{Y|S=s}\left|\right|P_{Y|X=s}\right)$, $\theta_{u}\left(P_{SX}\right):=\sum_{s\in S}P_{S}\left(s\right)D\left(P_{Y|X=s}\left|\right|P_{Y|S=s}\right)$, $\Theta \left(P_{SX}\right):=\left(-\theta_{l}\left(P_{SX}\right),\theta_{u}\left(P_{SX}\right)\right)$. Denote an arbitrary element of $F\times R_{\geq 0}\times P\left(S\times X\right)\times \Theta \left(P_{SX}\right)$ by $\left(\omega ,R,P_{SX},\theta \right)$, and set

$$\begin{array}{cc} \; & L\left(\kappa_{\alpha }\right):=\left\{\begin{array}{cc} (\omega ,R,P_{SX},\theta ):\; & \zeta \left(\kappa_{\alpha },\omega \right)-\rho \left(\kappa_{\alpha },\omega \right)\leq R<I_{P}\left(X;Y|S\right),\;P_{SXY}=P_{SX}P_{Y|X}\\ \; & \min\left\{E_{\mathrm{sp}}\left(P_{SX},\theta \right),E_{\mathrm{ex}}\left(R,P_{SX}\right),E_{b}\left(\kappa_{\alpha },\omega ,R\right)\right\}\geq \kappa_{\alpha } \end{array}\right\}, \end{array}$$

(6a) $$\begin{array}{cc} \; & \hat{L}\left(\kappa_{\alpha },\omega \right):=\left\{P_{\hat{U}\hat{V}\hat{W}}:\;D\left(P_{\hat{U}\hat{V}\hat{W}}\left|\right|P_{UV\hat{W}}\right)\leq \kappa_{\alpha },P_{\hat{W}|\hat{U}}=\omega \left(P_{\hat{U}}\right),P_{UV\hat{W}}=P_{UV}P_{\hat{W}|\hat{U}}\right\},\\ \; & E_{b}\left(\kappa_{\alpha },\omega ,R\right):=\left\{\begin{array}{cc} R-\zeta \left(\kappa_{\alpha },\omega \right)+\rho \left(\kappa_{\alpha },\omega \right), & \mathrm{if}\;0\leq R<\zeta (\kappa_{\alpha },\omega ),\\ \;\;\;\;\;\infty , & \mathrm{otherwise}, \end{array}\right. \end{array}$$

(6b) $$\begin{array}{cc} \; & \zeta \left(\kappa_{\alpha },\omega \right):=\max_{\begin{array}{c} P_{\hat{U}\hat{W}}:\;\exists \;P_{\hat{V}},P_{\hat{U}\hat{V}\hat{W}}\in \hat{L}\left(\kappa_{\alpha },\omega \right) \end{array}}I_{P}\left(\hat{U};\hat{W}\right), \end{array}$$

(6c) $$\begin{array}{cc} \; & \rho \left(\kappa_{\alpha },\omega \right):=\min_{\begin{array}{c} P_{\hat{V}\hat{W}}:\;\exists \;P_{\hat{U}},P_{\hat{U}\hat{V}\hat{W}}\in \hat{L}\left(\kappa_{\alpha },\omega \right) \end{array}}I_{P}\left(\hat{V};\hat{W}\right),\\ \; & E_{1}\left(\kappa_{\alpha },\omega \right):=\min_{\begin{array}{c} \left(P_{\tilde{U}\tilde{V}\tilde{W}},Q_{\tilde{U}\tilde{V}\tilde{W}}\right)\in T_{1}\left(\kappa_{\alpha },\omega \right) \end{array}}D(P_{\tilde{U}\tilde{V}\tilde{W}}\left|\right|Q_{\tilde{U}\tilde{V}\tilde{W}}), \end{array}$$

$$\begin{array}{cc} \; & E_{2}\left(\kappa_{\alpha },\omega ,R\right):=\left\{\begin{array}{cc} \min_{\begin{array}{c} \left(P_{\tilde{U}\tilde{V}\tilde{W}},Q_{\tilde{U}\tilde{V}\tilde{W}}\right)\in T_{2}\left(\kappa_{\alpha },\omega \right) \end{array}}D(P_{\tilde{U}\tilde{V}\tilde{W}}\left|\right|Q_{\tilde{U}\tilde{V}\tilde{W}})+E_{b}\left(\kappa_{\alpha },\omega ,R\right), & \mathrm{if}\;R<\zeta (\kappa_{\alpha },\omega ),\\ \;\;\;\;\;\;\infty , & \mathrm{otherwise}, \end{array}\right. \end{array}$$

$$\begin{array}{cc} \; & E_{3}\left(\kappa_{\alpha },\omega ,R,P_{SX}\right):=\left\{\begin{array}{cc} \min_{\begin{array}{c} \left(P_{\tilde{U}\tilde{V}\tilde{W}},Q_{\tilde{U}\tilde{V}\tilde{W}}\right)\in T_{3}\left(\kappa_{\alpha },\omega \right) \end{array}}D(P_{\tilde{U}\tilde{V}\tilde{W}}\left|\right|Q_{\tilde{U}\tilde{V}\tilde{W}})+E_{b}\left(\kappa_{\alpha },\omega ,R\right)+E_{\mathrm{ex}}\left(R,P_{SX}\right), & \\ \;\;\;\;\;\;\;\;\;\;\;\;\mathrm{if}\;R<\zeta (\kappa_{\alpha },\omega ), & \\ \min_{\begin{array}{c} \left(P_{\tilde{U}\tilde{V}\tilde{W}},Q_{\tilde{U}\tilde{V}\tilde{W}}\right)\in T_{3}\left(\kappa_{\alpha },\omega \right) \end{array}}D(P_{\tilde{U}\tilde{V}\tilde{W}}\left|\right|Q_{\tilde{U}\tilde{V}\tilde{W}})+\rho \left(\kappa_{\alpha },\omega \right)+E_{\mathrm{ex}}\left(R,P_{SX}\right), & \\ \;\;\;\;\;\;\;\;\;\;\;\;\mathrm{otherwise}, \end{array}\right. \end{array}$$

$$\begin{array}{cc} \; & E_{4}\left(\kappa_{\alpha },\omega ,R,P_{SX},\theta \right):=\left\{\begin{array}{cc} \min_{P_{\hat{V}}:P_{\hat{U}\hat{V}\hat{W}}\in \hat{L}\left(\kappa_{\alpha },\omega \right)}D(P_{\hat{V}}\left|\right|Q_{V})+E_{b}\left(\kappa_{\alpha },\omega ,R\right)+E_{m}\left(P_{SX},\theta \right)-\theta , & \\ \;\;\;\;\;\;\;\;\;\;\;\;\mathrm{if}\;R<\zeta (\kappa_{\alpha },\omega ), & \\ \min_{P_{\hat{V}}:P_{\hat{U}\hat{V}\hat{W}}\in \hat{L}\left(\kappa_{\alpha },\omega \right)}D(P_{\hat{V}}\left|\right|Q_{V})+\rho \left(\kappa_{\alpha },\omega \right)+E_{m}\left(P_{SX},\theta \right)-\theta , & \\ \;\;\;\;\;\;\;\;\;\;\;\;\mathrm{otherwise}, \end{array}\right.\\ \; & \mathrm{where}, \end{array}$$

(6d) $$\begin{array}{cc} \; & T_{1}\left(\kappa_{\alpha },\omega \right):=\left\{\begin{array}{cc} (P_{\tilde{U}\tilde{V}\tilde{W}},Q_{\tilde{U}\tilde{V}\tilde{W}}): & P_{\tilde{U}\tilde{W}}=P_{\hat{U}\hat{W}},\;P_{\tilde{V}\tilde{W}}=P_{\hat{V}\hat{W}},\;Q_{\tilde{U}\tilde{V}\tilde{W}}\\ & :=Q_{UV}P_{\tilde{W}|\tilde{U}}\mathrm{for}\;\mathrm{some}P_{\hat{U}\hat{V}\hat{W}}\in \hat{L}\left(\kappa_{\alpha },\omega \right) \end{array}\right\},\\ \; & T_{2}\left(\kappa_{\alpha },\omega \right):=\left\{\begin{array}{cc} (P_{\tilde{U}\tilde{V}\tilde{W}},Q_{\tilde{U}\tilde{V}\tilde{W}}): & P_{\tilde{U}\tilde{W}}=P_{\hat{U}\hat{W}},\;P_{\tilde{V}}=P_{\hat{V}},\;H_{P}\left(\tilde{W}|\tilde{V}\right)\geq H_{P}\left(\hat{W}|\hat{V}\right),\\ & Q_{\tilde{U}\tilde{V}\tilde{W}}:=Q_{UV}P_{\tilde{W}|\tilde{U}}\mathrm{for}\;\mathrm{some}P_{\hat{U}\hat{V}\hat{W}}\in \hat{L}\left(\kappa_{\alpha },\omega \right) \end{array}\right\},\\ \; & T_{3}\left(\kappa_{\alpha },\omega \right):=\left\{\begin{array}{cc} (P_{\tilde{U}\tilde{V}\tilde{W}},Q_{\tilde{U}\tilde{V}\tilde{W}}): & P_{\tilde{U}\tilde{W}}=P_{\hat{U}\hat{W}},\;P_{\tilde{V}}=P_{\hat{V}},\;Q_{\tilde{U}\tilde{V}\tilde{W}}:=Q_{UV}P_{\tilde{W}|\tilde{U}}\\ & \mathrm{for}\;\mathrm{some}P_{\hat{U}\hat{V}\hat{W}}\in \hat{L}\left(\kappa_{\alpha },\omega \right) \end{array}\right\}. \end{array}$$

We have the following lower bound for $\kappa (\kappa_{\alpha })$, which translates to an inner bound for R.

**Theorem 1**  (Inner bound via SHTCC scheme)**.** *$\kappa \left(\kappa_{\alpha }\right)\geq \kappa_{s}^{★}\left(\kappa_{\alpha }\right),$ where*

(7) $$\begin{array}{cc} \kappa_{s}^{★}\left(\kappa_{\alpha }\right) & :=\max_{\begin{array}{c} \left(\omega ,R,P_{SX},\theta \right)\in \;L\left(\kappa_{\alpha }\right) \end{array}}\min\{E_{1}\left(\kappa_{\alpha },\omega \right),E_{2}\left(\kappa_{\alpha },\omega ,R\right),E_{3}\left(\kappa_{\alpha },\omega ,R,P_{SX}\right),\\ \; & \;\;\;\;\;\;\;\;E_{4}\left(\kappa_{\alpha },\omega ,R,P_{SX},\theta \right)\}. \end{array}$$

The proof of Theorem 1 is presented in Section 4.1. The SHTCC scheme, which achieves the error-exponent pair $\left(\kappa_{\alpha },\kappa_{s}^{★}\left(\kappa_{\alpha }\right)\right)$, is a coding scheme analogous to separate source and channel coding for the lossy transmission of a source over a communication channel with correlated side-information at the receiver [45], however, with the objective of reliable HT. In this scheme, the source samples are first compressed to an index, which acts as the message to be transmitted over the channel. But, in contrast to standard communication problems, there is a need to protect certain messages more reliably than others; hence, an unequal error-protection scheme [39,42] is used. To describe briefly, the SHTCC scheme involves (i) the quantization and binning of u sequences, whose type $P_{u}$ is within a $\kappa_{\alpha }$-neighborhood (in terms of KL divergence) of $P_{U}$, using V as side information at the decision maker for decoding, and (ii) unequal error-protection channel coding scheme in [39] for protecting a special message which informs the decision maker that $P_{u}$ lies outside the $\kappa_{\alpha }$-neighborhood of $P_{U}$. The output of the channel decoder is processed by an empirical conditional entropy decoder which recovers the quantization codeword with the least conditional entropy with V. Since this decoder depends only on the empirical distributions of the observations, it is universal and useful in the hypothesis testing context, where multiple distributions are involved (as was first noted in [8]). The various factors $E_{1}$ to $E_{4}$ in (7) have natural interpretations in terms of events that could possibly result in a hypothesis testing error. Specifically, $E_{1}$ and $E_{2}$ correspond to the error events arising due to quantization and binning, respectively, while $E_{3}$ and $E_{4}$ correspond to the error events of wrongly decoding an ordinary channel codeword and special message codeword, respectively.

**Remark 1**  (Generalization of Han–Kobayashi inner bound)**.** *In Theorem 1 in [14], Han and Kobayashi obtained an inner bound on R for DHT over a noiseless channel. At a high level, their coding scheme involves type-based quantization of $u\in U^{n}$ sequences, whose type $P_{u}$ lies within a $\kappa_{\alpha }$-neighborhood of $P_{U}$, where $\kappa_{\alpha }$ is the desired type I error-exponent. As a corollary, Theorem 1 recovers the lower bound for $\kappa (\kappa_{\alpha })$ obtained in [14] by (i) setting $E_{\mathrm{ex}}\left(R,P_{SX}\right)$, $E_{m}\left(P_{SX},\theta \right)$ and $E_{m}\left(P_{SX},\theta \right)-\theta$ to ∞, which hold when the channel is noiseless, and (ii) maximizing over the set $\left\{\left(\omega ,R,P_{SX},\theta \right)\in F\times R_{\geq 0}\times P\left(S\times X\right)\times \Theta \left(P_{SX}\right):\zeta \left(\kappa_{\alpha },\omega \right)\leq R<I_{P}\left(X;Y|S\right),P_{SXY}:=P_{SX}P_{Y|X}\right\}\subseteq L\left(\kappa_{\alpha }\right)$ in (7). Then, note that the terms $E_{2}\left(\kappa_{\alpha },\omega ,R\right)$, $E_{3}\left(\kappa_{\alpha },\omega ,R,P_{SX}\right)$ and $E_{4}\left(\kappa_{\alpha },\omega ,R,P_{SX},\theta \right)$ all equal ∞, and thus the inner bound in Theorem 1 reduces to that given in Theorem 1 in [14].*

**Remark 2**  (Improvement via time-sharing)**.** *Since the lower bound on $\kappa (\kappa_{\alpha })$ in Theorem 1 is not necessarily concave, a tighter bound can be obtained using the technique of time-sharing similar to Theorem 3 in [14]. We omit its description, as it is cumbersome, although straightforward.*

Theorem 1 also recovers the lower bound for the optimal type II error-exponent for a fixed type I error probability constraint established in Theorem 2 in [10] by letting $\kappa_{\alpha }\to 0$. The details are provided in Appendix A. Further, specializing the lower bound in Theorem 1 to the case of TAI, i.e., when $Q_{UV}=P_{U}P_{V}$, we obtain the following corollary which characterizes the optimal type II error-exponent for TAI established in Proposition 7 in [10] as a special case.

**Corollary 1**  (Inner bound for TAI)**.** *Let $P_{UV}\in P\left(U\times V\right)$ be an arbitrary distribution and $Q_{UV}=P_{U}P_{V}$. Then,*

(8) $$\begin{array}{c} \kappa \left(\kappa_{\alpha }\right)\geq \kappa_{s}^{★}\left(\kappa_{\alpha }\right)\geq \kappa_{i}^{★}\left(\kappa_{\alpha }\right), \end{array}$$

*where*

(9) $$\begin{array}{cc} \; & \kappa_{i}^{★}\left(\kappa_{\alpha }\right):=\max_{\begin{array}{c} \left(\omega ,P_{SX},\theta \right)\in L^{★}\left(\kappa_{\alpha }\right) \end{array}}\min\left\{E_{1}^{i}\left(\kappa_{\alpha },\omega \right),E_{2}^{i}\left(\kappa_{\alpha },\omega ,P_{SX}\right),E_{3}^{i}\left(\kappa_{\alpha },\omega ,P_{SX},\theta \right)\right\},\\ \; & L^{★}\left(\kappa_{\alpha }\right):=\left\{\begin{array}{cc} \; & \left(\omega ,P_{SX},\theta \right)\in F\times P\left(S\times X\right)\times \Theta \left(P_{SX}\right):\zeta \left(\kappa_{\alpha },\omega \right)<I_{P}\left(X;Y|S\right),\\ \; & P_{SXY}:=P_{SX}P_{Y|X},\;\min\left\{E_{\mathrm{sp}}\left(P_{SX},\theta \right),E_{\mathrm{ex}}\left(\zeta \left(\kappa_{\alpha },\omega \right),P_{SX}\right)\right\}\geq \kappa_{\alpha } \end{array}\right\},\\ \; & E_{1}^{i}\left(\kappa_{\alpha },\omega \right):=\min_{\begin{array}{c} P_{\hat{V}\hat{W}}:\exists P_{\hat{U}\hat{V}\hat{W}}\in \hat{L}\left(\kappa_{\alpha },\omega \right) \end{array}}I_{P}\left(\hat{V};\hat{W}\right)+D(P_{\hat{V}}\left|\right|P_{V}),\\ \; & E_{2}^{i}\left(\kappa_{\alpha },\omega ,P_{SX}\right):=\rho \left(\kappa_{\alpha },\omega \right)+E_{\mathrm{ex}}\left(\zeta \left(\kappa_{\alpha },\omega \right),P_{SX}\right),\\ \; & E_{3}^{i}\left(\kappa_{\alpha },\omega ,P_{SX},\theta \right):=\rho \left(\kappa_{\alpha },\omega \right)+E_{\mathrm{sp}}\left(P_{SX},\theta \right)-\theta , \end{array}$$

*and $\hat{L}\left(\kappa_{\alpha },\omega \right)$, $\zeta (\kappa_{\alpha },\omega )$ and $\rho (\kappa_{\alpha },\omega )$ are defined in (6a), (6b) and (6c), respectively. In particular,*

(10) $$\begin{array}{c} \lim_{\kappa_{\alpha }\to 0}\kappa \left(\kappa_{\alpha }\right)=\kappa_{s}^{★}\left(0\right)=\kappa_{i}^{★}\left(0\right)=\max_{\begin{array}{c} P_{W|U}:I_{P}\left(U;W\right)\leq C\left(P_{Y|X}\right),\\ P_{UVW}=P_{UV}P_{W|U} \end{array}}I_{P}\left(V;W\right), \end{array}$$

*where |W|≤|U|+1 and $C(P_{Y|X})$ denotes the capacity of the channel $P_{Y|X}$.*

The proof of Corollary 1 is given in Section 4.2. Its achievability follows from a special case of the SHTCC scheme without binning at the encoder.

Next, we consider *testing against dependence* (TAD) for which $Q_{UV}$ is an arbitrary joint distribution and $P_{UV}=Q_{U}Q_{V}$. Theorem 1 specialized to TAD gives the following corollary.

**Corollary 2**  (Inner bound for TAD)**.** *Let $Q_{UV}\in P\left(U\times V\right)$ be an arbitrary distribution and $P_{UV}=Q_{U}Q_{V}$. Then,*

(11) $$\begin{array}{c} \kappa \left(\kappa_{\alpha }\right)\geq \kappa_{s}^{★}\left(\kappa_{\alpha }\right)=\kappa_{d}^{★}\left(\kappa_{\alpha }\right):=\max_{\begin{array}{c} \left(\omega ,P_{SX},\theta \right)\\ \in L^{★}\left(\kappa_{\alpha }\right) \end{array}}\min\left\{E_{1}^{d}\left(\kappa_{\alpha },\omega \right),E_{2}^{d}\left(\kappa_{\alpha },\omega ,P_{SX}\right),E_{3}^{d}\left(P_{SX},\theta \right)\right\}, \end{array}$$

*where*

$$\begin{array}{cc} \; & E_{1}^{d}\left(\kappa_{\alpha },\omega \right):=\min_{\begin{array}{c} \left(P_{\tilde{U}\tilde{V}\tilde{W}},Q_{\tilde{U}\tilde{V}\tilde{W}}\right)\\ \in T_{1}\left(\kappa_{\alpha },\omega \right) \end{array}}D(P_{\tilde{U}\tilde{V}\tilde{W}}\left|\right|Q_{\tilde{U}\tilde{V}\tilde{W}})\geq \min_{\begin{array}{c} \left(P_{\hat{V}\hat{W}},Q_{V\hat{W}}\right):\;P_{\hat{U}\hat{V}\hat{W}}\in \hat{L}\left(\kappa_{\alpha },\omega \right),\\ Q_{UV\hat{W}}=Q_{UV}P_{\hat{W}|\hat{U}} \end{array}}D(P_{\hat{V}\hat{W}}\left|\right|Q_{V\hat{W}}),\\ \; & E_{2}^{d}\left(\kappa_{\alpha },\omega ,P_{SX}\right):=E_{\mathrm{ex}}\left(\zeta \left(\kappa_{\alpha },\omega \right),P_{SX}\right),\\ \; & E_{3}^{d}\left(P_{SX},\theta \right):=E_{\mathrm{sp}}\left(P_{SX},\theta \right)-\theta , \end{array}$$

*and $\hat{L}\left(\kappa_{\alpha },\omega \right)$, $T_{1}\left(\kappa_{\alpha },\omega \right)$ and $L^{★}\left(\kappa_{\alpha }\right)$ are given in (6a), (6d) and (9), respectively. In particular,*

(12) $$\begin{array}{c} \lim_{\kappa_{\alpha }\to 0}\kappa \left(\kappa_{\alpha }\right)\geq \kappa_{s}^{★}\left(0\right)=\kappa_{d}^{★}\left(0\right)\geq \kappa_{\mathrm{TAD}}^{★}, \end{array}$$

*where*

$$\begin{array}{c} \kappa_{\mathrm{TAD}}^{★}=\max_{\begin{array}{c} (P_{W|U},P_{SX}):\\ I_{Q}\left(W;U\right)\leq I_{P}\left(X;Y|S\right),\\ Q_{UVW}=Q_{UV}P_{W|U},\\ P_{SXY}=P_{SX}P_{Y|X} \end{array}}\min\left\{D(Q_{V}Q_{W}\left|\right|Q_{VW}),E_{\mathrm{ex}}\left(I_{Q}\left(U;W\right),P_{SX}\right),\theta_{l}\left(P_{SX}\right)\right\}, \end{array}$$

*and |W|≤|U|+1.*

The proof of Corollary 2 is given in Section 4.3. Note that the expression for $\kappa_{s}^{★}\left(\kappa_{\alpha }\right)$ given in (11) is relatively simpler to compute compared to that in Theorem 1. This will be handy in showing that the JHTCC scheme strictly outperforms the SHTCC scheme, which we highlight via an example in Section 3.3 below.

### 3.2. Inner Bound via JHTCC Scheme

It is well known that joint source-channel coding schemes offer advantages over separation-based coding schemes in several information theoretic problems, such as the transmission of correlated sources over a multiple-access channel [40,46] and the error-exponent in the lossless or lossy transmission of a source over a noisy channel [42,47]. Recently, it was shown via an example in [10] that joint schemes also achieve a strictly larger type II error-exponent in DHT problems compared to a separation-based scheme in some scenarios. Motivated by this, we present an inner bound on R using a generalization of the JHTCC scheme in [10].

Let W and S be arbitrary finite sets, and $F^{\prime }$ denote the set of all continuous mappings from P(U×S) to P(W|U×S), where P(W|U×S) is the set of all conditional distributions $P_{W|US}$. Let $\left(P_{S},\omega^{\prime }\left(\cdot ,P_{S}\right),P_{X|USW},P_{X^{\prime }|US}\right)$ denote an arbitrary element of $P\left(S\right)\times F^{\prime }\times P\left(X|U\times S\times W\right)\times P\left(X|U\times S\right)$, and define

$$\begin{array}{ccc} & L_{h}\left(\kappa_{\alpha }\right):=\left\{\left(P_{S},\omega^{\prime }\left(\cdot ,P_{S}\right),P_{X|USW},P_{X^{\prime }|US}\right):E_{b}^{\prime }\left(\kappa_{\alpha },\omega^{\prime },P_{S},P_{X|USW}\right)\geq \kappa_{\alpha }\right\},\\ & {\hat{L}}_{h}\left(\kappa_{\alpha },\omega^{\prime },P_{S},P_{X|USW}\right)\\ & :=\left\{\begin{array}{cc} (P_{\hat{U}\hat{V}\hat{W}\hat{Y}S}: & \;D(P_{\hat{U}\hat{V}\hat{W}\hat{Y}|S}\left|\right|P_{UV\hat{W}Y|S}\left|P_{S}\right)\leq \kappa_{\alpha },\;P_{SUV\hat{W}XY}:=P_{S}P_{UV}P_{\hat{W}|\hat{U}S}P_{X|USW}P_{Y|X},\\ & P_{\hat{W}|\hat{U}S}=\omega^{\prime }\left(P_{\hat{U}},P_{S}\right) \end{array}\right\},\\ & E_{b}^{\prime }\left(\kappa_{\alpha },\omega^{\prime },P_{S},P_{X|USW}\right):=\rho^{\prime }\left(\kappa_{\alpha },\omega^{\prime },P_{S},P_{X|USW}\right)-\zeta_{q}^{\prime }\left(\kappa_{\alpha },\omega^{\prime },P_{S}\right),\\ & \zeta^{\prime }\left(\kappa_{\alpha },\omega^{\prime },P_{S}\right):=\max_{\begin{array}{c} P_{\hat{U}\hat{W}S}:\;\exists \;P_{\hat{V}\hat{Y}}s.t.\\ P_{\hat{U}\hat{V}\hat{W}\hat{Y}S}\;\in {\hat{L}}_{h}\left(\kappa_{\alpha },\omega^{\prime },P_{S},P_{X|USW}\right) \end{array}}I_{P}\left(\hat{U};\hat{W}|S\right),\\ & \rho^{\prime }\left(\kappa_{\alpha },\omega^{\prime },P_{S},P_{X|USW}\right):=\min_{\begin{array}{c} P_{\hat{V}\hat{W}\hat{Y}S}:\;\exists \;P_{\hat{U}}s.t.\\ P_{\hat{U}\hat{V}\hat{W}\hat{Y}S}\;\in {\hat{L}}_{h}\left(\kappa_{\alpha },\omega^{\prime },P_{S},P_{X|USW}\right) \end{array}}I_{P}\left(\hat{Y},\hat{V};\hat{W}|S\right),\\ & E_{1}^{\prime }\left(\kappa_{\alpha },\omega^{\prime }\right):=\min_{\begin{array}{c} \left(P_{\tilde{U}\tilde{V}\tilde{W}\tilde{Y}S},Q_{\tilde{U}\tilde{V}\tilde{W}\tilde{Y}S}\right)\in T_{1}^{\prime }\left(\kappa_{\alpha },\omega^{\prime }\right) \end{array}}D(P_{\tilde{U}\tilde{V}\tilde{W}\tilde{Y}|S}\left|\right|Q_{\tilde{U}\tilde{V}\tilde{W}\tilde{Y}|S}\left|P_{S}\right),\\ & E_{2}^{\prime }\left(\kappa_{\alpha },\omega^{\prime },P_{S},P_{X|USW}\right):=\min_{\begin{array}{c} \left(P_{\tilde{U}\tilde{V}\tilde{W}\tilde{Y}S},Q_{\tilde{U}\tilde{V}\tilde{W}\tilde{Y}S}\right)\in T_{2}^{\prime }\left(\kappa_{\alpha },\omega^{\prime },P_{S},P_{X|USW}\right) \end{array}}D(P_{\tilde{U}\tilde{V}\tilde{W}\tilde{Y}|S}\left|\right|Q_{\tilde{U}\tilde{V}\tilde{W}\tilde{Y}|S}\left|P_{S}\right)\\ & \;\;\;\;\;\;\;\;\;\;\;\;\;+E_{b}^{\prime }\left(\kappa_{\alpha },\omega^{\prime },P_{S},P_{X|USW}\right),\\ & E_{3}^{\prime }\left(\kappa_{\alpha },\omega^{\prime },P_{S},P_{X|USW},P_{X^{\prime }|US}\right):=\min_{\begin{array}{c} P_{\hat{V}\hat{Y}S}:P_{\hat{U}\hat{V}\hat{W}\hat{Y}S}\in {\hat{L}}_{h}\left(\kappa_{\alpha },\omega^{\prime },P_{S},P_{X|USW}\right) \end{array}}D(P_{\hat{V}\hat{Y}|S}\left|\right|Q_{VY^{\prime }|S}\left|P_{S}\right)\\ & \;\;\;\;\;\;\;\;\;\;\;\;\;+E_{b}^{\prime }\left(\kappa_{\alpha },\omega^{\prime },P_{S},P_{X|USW}\right),\\ & Q_{SUVX^{\prime }Y^{\prime }}:=P_{S}Q_{UV}P_{X^{\prime }|US}P_{Y^{\prime }|X^{\prime }},\;P_{Y^{\prime }|X^{\prime }}:=P_{Y|X},\\ & T_{1}^{\prime }\left(\kappa_{\alpha },\omega^{\prime },P_{S},P_{X|USW}\right):=\left\{\begin{array}{cc} (P_{\tilde{U}\tilde{V}\tilde{W}\tilde{Y}S}, & P_{\tilde{U}\tilde{W}S}=P_{\hat{U}\hat{W}S},\;P_{\tilde{V}\tilde{W}\tilde{Y}S}=P_{\hat{V}\hat{W}\hat{Y}S},\\ \;\;\;Q_{\tilde{U}\tilde{V}\tilde{W}\tilde{Y}S}): & Q_{S\tilde{U}\tilde{V}\tilde{W}\tilde{X}\tilde{Y}}:=P_{S}Q_{UV}P_{\tilde{W}|\tilde{U}S}\;P_{X|USW}P_{Y|X}\\ & \mathrm{for}\;\mathrm{some}P_{\hat{U}\hat{V}\hat{W}\hat{Y}S}\in {\hat{L}}_{h}\left(\kappa_{\alpha },\omega^{\prime },P_{S},P_{X|USW}\right) \end{array}\right\},\\ & T_{2}^{\prime }\left(\kappa_{\alpha },\omega^{\prime },P_{S},P_{X|USW}\right):=\left\{\begin{array}{cc} (P_{\tilde{U}\tilde{V}\tilde{W}\tilde{Y}S}, & P_{\tilde{U}\tilde{W}S}=P_{\hat{U}\hat{W}S},\;P_{\tilde{V}\tilde{Y}S}=P_{\hat{V}\hat{Y}S},\\ \;\;\;\;Q_{\tilde{U}\tilde{V}\tilde{W}\tilde{Y}S}): & H_{P}\left(\tilde{W}|\tilde{V},\tilde{Y},S\right)\geq H_{P}\left(\hat{W}|\hat{V},\hat{Y},S\right),\\ & Q_{S\tilde{U}\tilde{V}\tilde{W}\tilde{X}\tilde{Y}}:=P_{S}Q_{UV}P_{\tilde{W}|\tilde{U}S}\;P_{X|USW}P_{Y|X}\\ & \mathrm{for}\;\mathrm{some}P_{\hat{U}\hat{V}\hat{W}\hat{Y}S}\in {\hat{L}}_{h}\left(\kappa_{\alpha },\omega^{\prime },P_{S},P_{X|USW}\right) \end{array}\right\}. & \end{array}$$

Then, we have the following result.

**Theorem 2**  (Inner bound via JHTCC scheme)**.**

(13) $$\begin{array}{cc} & \kappa \left(\kappa_{\alpha }\right)\geq \max\left\{\kappa_{h}^{★}\left(\kappa_{\alpha }\right),\kappa_{u}^{★}\left(\kappa_{\alpha }\right)\right\}, \end{array}$$

*where*

$$\begin{array}{cc} & \kappa_{h}^{★}\left(\kappa_{\alpha }\right):=\max_{\begin{array}{c} \left(P_{S},\omega^{\prime },P_{X|USW},P_{X^{\prime }|US}\right)\in \;L_{h}\left(\kappa_{\alpha }\right) \end{array}}\min\{E_{1}^{\prime }\left(\kappa_{\alpha },\omega^{\prime }\right),\;E_{2}^{\prime }\left(\kappa_{\alpha },\omega^{\prime },P_{S},P_{X|USW}\right), \end{array}$$

$$\begin{array}{cc} & \;\;\;\;\;\;\;\;\;E_{3}^{\prime }\left(\kappa_{\alpha },\omega^{\prime },P_{S},P_{X|USW},P_{X^{\prime }|US}\right)\},\\ & \kappa_{u}^{★}\left(\kappa_{\alpha }\right):=\max_{\begin{array}{c} \left(P_{S},P_{X|US}\right)\in P\left(S\right)\times P\left(X|S\times U\right) \end{array}}\kappa_{u}\left(\kappa_{\alpha },P_{S},P_{X|US}\right),\\ & \kappa_{u}\left(\kappa_{\alpha },P_{S},P_{X|US}\right):=\min_{\begin{array}{c} P_{S}P_{\hat{V}\hat{Y}}:D\left(P_{\hat{V}\hat{Y}|S}\left|\right|P_{VY|S}|P_{S}\right)\leq \kappa_{\alpha } \end{array}}D\left(P_{\hat{V}\hat{Y}|S}\left|\right|Q_{VY|S}|P_{S}\right),\\ & P_{SUVXY}=P_{S}P_{UV}P_{X|US}P_{Y|X}\;and\;Q_{SUVXY}=P_{S}Q_{UV}P_{X|US}P_{Y|X}. \end{array}$$

The proof of Theorem 2 is given in Section 4.4, and utilizes a generalization of hybrid coding scheme [40] to achieve the stated inner bound. Specifically, the error-exponent pair $\left(\kappa_{\alpha },\kappa_{h}^{★}\left(\kappa_{\alpha }\right)\right)$ is achieved using type-based hybrid coding, while $\left(\kappa_{\alpha },\kappa_{u}^{★}\left(\kappa_{\alpha }\right)\right)$ is realized by uncoded transmission, in which the channel input X is generated as the output of a DMC $P_{X|U}$ with input U (along with time sharing). In standard hybrid coding, the source sequence is first quantized via joint typicality and the channel input is then chosen as a function of both the original source sequence and its quantization. At the decoder, the quantized codeword is first recovered using the channel output and side information via joint typicality decoding, and an estimate of the source sequence is output as a function of the channel output and recovered codeword. The quantization part forms the *digital* part of the scheme, while the use of the source sequence for encoding and channel output for decoding comprises the *analog* part. The scheme derives its name from these joint hybrid digital-analog operations. In the HT context considered here, the aforementioned source quantization is replaced by a type-based quantization at the encoder, and the joint typicality decoder is replaced by a universal empirical conditional entropy decoder. We note that Theorem 2 recovers the lower bound on the optimal type II error-exponent proved in Theorem 5 in [10]. The details are provided in Appendix B.

Next, we provide a comparison between the SHTCC and JHTCC bounds via an example as mentioned earlier.

### 3.3. Comparison of Inner Bounds

We compare the inner bounds established in Theorem 1 and Theorem 2 for a simple setting of TAD over a BSC. For this purpose, we will use the inner bound $\kappa_{d}^{★}\left(\kappa_{\alpha }\right)$ stated in Corollary 2 and $\kappa_{u}^{★}\left(\kappa_{\alpha }\right)$ that is achieved by uncoded transmission. Our objective is to illustrate that the JHTCC scheme achieves a strictly tighter bound on R compared to the SHTCC scheme, at least for some points of the trade-off.

**Example 1.**  *Let p,q∈[0,0.5], U=V=X=Y=S={0,1},*

$$Q_{UV}=\left[\begin{array}{cc} q & 0.5-q\\ 0.5-q & q \end{array}\right],\;\;\;\;P_{Y|X}=\left[\begin{array}{cc} 1-p & p\\ p & 1-p \end{array}\right],and\;P_{UV}=Q_{U}Q_{V}.$$

**Figure 2:**
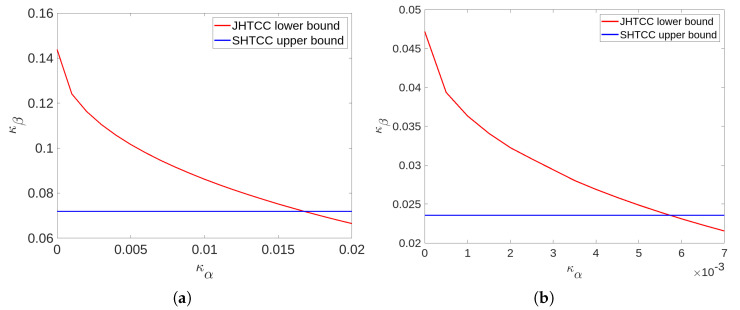
Comparison of the error-exponents trade-off achieved by the SHTCC and JHTCC schemes for TAD over a BSC in Example 1 with parameters p=0.25,q=0 for (**a**) and p=0.35,q=0 for (**b**). The red curve shows $\left(\kappa_{\alpha },\kappa_{u}^{★}\left(\kappa_{\alpha }\right)\right)$ pairs achieved by uncoded transmission while the blue line plots $\left(\kappa_{\alpha },E_{\mathrm{ex}}\left(0\right)\right)$. The joint scheme clearly achieves a better error-exponent trade-off for values of $\kappa_{\alpha }$ below a threshold which depends on the transition kernel of the channel. In particular, a more uniform channel results in a lesser threshold.

**Figure 3:**
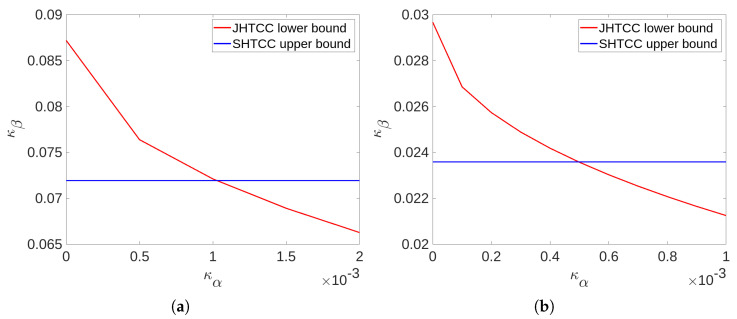
Comparison of the error-exponents trade-off achieved by the SHTCC and JHTCC schemes for Example 1 with parameters p=0.25,q=0.05 for (**a**) and p=0.35,q=0.05 for (**b**). The JHTCC scheme improves over the separation based scheme for small values of $\kappa_{\alpha }$; however, the region of improvement is reduced compared to Figure 2 as the source is more uniformly distributed.

A comparison of the inner bounds achieved by the SHTCC and JHTCC schemes for the above example are shown in Figure 2 and Figure 3, where we plot the error-exponents trade-off achieved by uncoded transmission (a lower bound for the JHTCC scheme), and the expurgated exponent at a zero rate:

$$\begin{array}{c} E_{\mathrm{ex}}\left(0\right):=\max_{P_{SX}\in P\left(S\times X\right)}E_{\mathrm{ex}}\left(P_{SX},0\right)=-0.25\log\left(4p\left(1-p\right)\right), \end{array}$$

which is an upper bound on $\kappa_{d}^{★}\left(\kappa_{\alpha }\right)$ for any $\kappa_{\alpha }\geq 0$. To compute $E_{\mathrm{ex}}\left(0\right)$, we used the closed-form expression for $E_{\mathrm{ex}}\left(\cdot \right)$ given in Problem 10.26(c) in [38]. Clearly, it can be seen that the JHTCC scheme outperforms SHTCC scheme for $\kappa_{\alpha }$ below a threshold, which depends on the source and channel distributions. In particular, the threshold below which improvement is seen is reduced when the channel or the source becomes more uniform. The former behavior can be seen directly by comparing the subplots in Figure 2 and Figure 3, while the latter can be noted by comparing Figure 2a with Figure 3a, or Figure 2b with Figure 3b.

## 4. Proofs

### 4.1. Proof of Theorem 1

We will show the achievability of the error-exponent pair $\left(\kappa_{\alpha },\kappa_{s}^{★}\left(\kappa_{\alpha }\right)\right)$ by constructing a suitable ensemble of HT codes, and showing that the expected type I and type II error probabilities (over this ensemble) satisfy (5) for the pair $\left(\kappa_{\alpha },\kappa_{s}^{★}\left(\kappa_{\alpha }\right)\right)$. Then, an expurgation argument [44] will be used to show the existence of a HT code that satisfies (5) for the same error-exponent pair, thus showing that $(\kappa_{\alpha },\kappa_{s}^{★}\left(\kappa_{\alpha }\right))\in R$ as desired.

Let n∈N, |W|<∞, $\kappa_{\alpha }>0$, $\left(\omega ,R,P_{SX},\theta \right)\in L\left(\kappa_{\alpha }\right)$, $R^{\prime }:=\zeta \left(\kappa_{\alpha },\omega \right)$, and η>0 be a small number. Additionally, suppose that R≥0 satisfies

(14) $$\begin{array}{cc} \; & \zeta \left(\kappa_{\alpha },\omega \right)-\rho \left(\kappa_{\alpha },\omega \right)\leq R<I_{P}\left(X;Y|S\right), \end{array}$$

where $\zeta (\kappa_{\alpha },\omega )$ and $\rho (\kappa_{\alpha },\omega )$ are defined in (6b) and (6c), respectively. The SHTCC scheme is as follows:

**Encoding:** The observer’s encoder is composed of two stages, a *source encoder* followed by a *channel encoder*.
**Source encoder:** The source encoding comprises a quantization scheme followed by binning to reduce the rate if necessary.
**Quantization codebook:** Let

(15) $$\begin{array}{c} D_{n}\left(P_{U},\eta \right):=\left\{P_{\hat{U}}\in T\left(U^{n}\right):D(P_{\hat{U}}\left|\right|P_{U})\leq \kappa_{\alpha }+\eta \right\}. \end{array}$$

Consider some ordering on the types in $D_{n}\left(P_{U},\eta \right)$ and denote the elements as $P_{{\hat{U}}_{i}}$ for $i\in \left[|D_{n}\left(P_{U},\eta \right)|\right]$. For each type $P_{{\hat{U}}_{i}}\in D_{n}\left(P_{U},\eta \right)$, $i\in \left[|D_{n}\left(P_{U},\eta \right)|\right]$, choose a joint type variable $P_{{\hat{U}}_{i}{\hat{W}}_{i}}\in T\left(U^{n}\times W^{n}\right)$ such that

(16a) $$\begin{array}{cc} \; & D\left(P_{{\hat{W}}_{i}|{\hat{U}}_{i}}\left|\right|P_{W_{i}|U}|P_{{\hat{U}}_{i}}\right)\leq \frac{\eta }{3}, \end{array}$$

(16b) $$\begin{array}{cc} \; & I_{P}\left({\hat{U}}_{i};{\hat{W}}_{i}\right)\leq R^{\prime }+\frac{\eta }{3}, \end{array}$$

where $P_{W_{i}|U}=\omega \left(P_{{\hat{U}}_{i}}\right)$. Note that this is always possible for *n* sufficiently large by the definition of $R^{\prime }$.

Let

(17a) $$\begin{array}{cc} D_{n}\left(P_{UW},\eta \right) & :=\left\{P_{{\hat{U}}_{i}{\hat{W}}_{i}}:i\in \left[|D_{n}\left(P_{U},\eta \right)|\right]\right\}, \end{array}$$

(17b) $$\begin{array}{cc} \;\;\;\;\;\;\;R_{i}^{\prime } & :=I_{P}\left({\hat{U}}_{i};{\hat{W}}_{i}\right)+\left(\eta /3\right),i\in \left[|D_{n}\left(P_{U},\eta \right)|\right], \end{array}$$

(17c) $$\begin{array}{cc} \;\;\;M_{i}^{\prime } & :=\left[1+\sum_{k=1}^{i-1}e^{nR_{k}^{\prime }}:\sum_{k=1}^{i}e^{nR_{k}^{\prime }}\right], \end{array}$$

and $B_{W,n}=\left\{W\left(j\right),1\leq j\leq \sum_{i=1}^{|D_{n}\left(P_{U},\eta \right)|}\left|M_{i}^{\prime }\right|\right\}$ denote a random quantization codebook such that the codeword $W\left(j\right)∼\mathrm{Unif}\left[T_{n}\left(P_{{\hat{W}}_{i}}\right)\right]$, if $j\in M_{i}^{\prime }$ for some $i\in \left[|D_{n}\left(P_{U},\eta \right)|\right]$. Denote a realization of $B_{W,n}$ by $B_{W,n}=\left\{w\left(j\right)\in W^{n},1\leq j\leq \sum_{i=1}^{|D_{n}\left(P_{U},\eta \right)|}\left|M_{i}^{\prime }\right|\right\}$.

**Quantization scheme:** For a given codebook $B_{W,n}$ and $u\in T_{n}\left(P_{{\hat{U}}_{i}}\right)$ such that $P_{{\hat{U}}_{i}}\in D_{n}\left(P_{U},\eta \right)$ for some $i\in \left[|D_{n}\left(P_{U},\eta \right)|\right]$, let

$$\tilde{M}\left(u,B_{W,n}\right):=\left\{j\in M_{i}^{\prime }:\;w\left(j\right)\in B_{W,n},\left(u,w\left(j\right)\right)\in T_{n}\left(P_{{\hat{U}}_{i}{\hat{W}}_{i}}\right),\;P_{{\hat{U}}_{i}{\hat{W}}_{i}}\in D_{n}\left(P_{UW},\eta \right)\right\}.$$

If $|\tilde{M}\left(u,B_{W,n}\right)|\geq 1$, let $M^{\prime }\left(u,B_{W,n}\right)$ denote an index selected uniformly at random from the set $\tilde{M}\left(u,B_{W,n}\right)$, otherwise, set $M^{\prime }\left(u,B_{W,n}\right)=0$. Denoting the support of $M^{\prime }\left(u,B_{W,n}\right)$ by $M^{\prime }$, we have for sufficiently large *n* that

(18) $$\begin{array}{cc} |M^{\prime }|\leq 1+\sum_{i=1}^{|D_{n}\left(P_{U},\eta \right)|}e^{nR_{i}^{\prime }} & \leq 1+|D_{n}\left(P_{U},\eta \right)|e^{{\overset{\max}{P_{\hat{U}\hat{W}}\in D_{n}\left(P_{UW},\eta \right)}}^{nI\left(\hat{U};\hat{W}\right)+\left(n\eta /3\right)}}\leq e^{n(R^{\prime }+\eta )}, \end{array}$$

where the last inequality uses (16b) and $|D_{n}\left(P_{U},\eta \right)|\leq {\left(n+1\right)}^{\left|U\right|}$. **Binning:** If $|M^{\prime }\left|>|M\right|$, then the source encoder performs binning as described below. Let $R_{n}:=\log\left(e^{nR}/\left|D_{n}\left(P_{U},\eta \right)\right|\right)$, $M_{i}:=\left[1+\left(i-1\right)R_{n}:iR_{n}\right],\;i\in \left[|D_{n}\left(P_{U},\eta \right)|\right]$, and $M:=\left\{0\right\}⋃\left\{\cup_{i\in [|D_{n}\left(P_{U},\eta \right)|]}M_{i}\right\}.$ Note that

(19) $$\begin{array}{c} e^{nR_{n}}\geq e^{nR-|U|\log(n+1)}. \end{array}$$

Let $f_{B}$ denote the random binning function such that for each $j\in M_{i}^{\prime }$, $f_{B}\left(j\right)∼\mathrm{Unif}\;[|M_{i}\left|\right]$ for $i\in \left[|D_{n}\left(P_{U},\eta \right)|\right]$, and $f_{B}\left(0\right)=0$ with probability one. Denote a realization of $f_{B}\left(j\right)$ by $f_{b}$, where $f_{b}:M^{\prime }\to M$. Given a codebook $B_{W,n}$ and binning function $f_{b}$, the source encoder outputs $M=f_{b}\left(M^{\prime }\left(u,B_{W,n}\right)\right)$ for $u\in U^{n}$. If $|M^{\prime }\left|\leq |M\right|$, then $f_{b}$ is taken to be the identity map (no binning), and in this case, $M=M^{\prime }\left(u,B_{W,n}\right)$.

**Channel codebook:** Let $B_{X,n}:=\left\{X\left(m\right)\in X^{n},m\in M\right\}$ denote a random channel codebook generated as follows. Without loss of generality, denote the elements of the set S=X as 1,…,|X|. The codeword length *n* is divided into |S|=|X| blocks, where the length of the first block is $\left\lceil P_{S}\left(1\right)n\right\rceil$, the second block is $\left\lceil P_{S}\left(2\right)n\right\rceil$, so on and so forth, and the length of the last block is chosen such that the total length is *n*. For i∈[|X|], let $k_{i}:=\sum_{l=1}^{i-1}\left\lceil P_{S}\left(l\right)n\right\rceil +1$ and ${\bar{k}}_{i}:=\sum_{l=1}^{i}\left\lceil P_{S}\left(l\right)n\right\rceil$, where the empty sum is defined to be zero. Let $s\in X^{n}$ be such that $s_{k_{i}}^{{\bar{k}}_{i}}=i$, i.e., the elements of s equal *i* in the $i^{th}$ block for i∈[|X|]. Let X(0)=s with probability one, and the remaining codewords X(m),m∈M∖{0} be constant composition codewords [38] selected such that $X_{k_{i}}^{{\bar{k}}_{i}}\left(m\right)∼\mathrm{Unif}\left[T_{\left\lceil P_{S}\left(i\right)n\right\rceil }\left({\hat{P}}_{X|S}\left(\cdot |i\right)\right)\right]$, where ${\hat{P}}_{X|S}$ is such that $T_{\left\lceil P_{S}\left(i\right)n\right\rceil }\left({\hat{P}}_{X|S}\left(\cdot |i\right)\right)$ is non-empty and $D({\hat{P}}_{X|S}\left|\right|P_{X|S}\left|P_{S}\right)\leq \frac{\eta }{3}$. Denote a realization of $B_{X,n}$ by $B_{X,n}:=\left\{x\left(m\right)\in X^{n},m\in M\right\}$. Note that for m∈M∖{0} and large *n*, the codeword pair (x(0),x(m)) has joint type (approx) $P_{x(0)x(j)}={\hat{P}}_{SX}:=P_{S}{\hat{P}}_{X|S}$.

**Channel encoder:** For a given $B_{X,n}$, the channel encoder outputs x=x(m) for output *m* from the source encoder. Denote this map by $f_{B_{X,n}}:M\to X^{n}$.

**Encoder:** Denote by $f_{n}:U^{n}\to P\left(X^{n}\right)$ the encoder induced by all the above operations, i.e., $f_{n}\left(\cdot |u\right)=f_{B_{X,n}}\circ f_{b}\left(M^{\prime }\left(u,B_{W,n}\right)\right)$.

**Decision function:** The decision function consists of three parts, a channel decoder, a source decoder and a tester.

**Channel decoder:** The channel decoder first performs a Neyman–Pearson test on the channel output y according to ${\tilde{\Pi }}_{\theta }:Y^{n}\to \left\{0,1\right\}$, where

(20) $$\begin{array}{cc} {\tilde{\Pi }}_{\theta }\left(y\right) & :=𝟙\left(\sum_{i=1}^{n}\log\left(\frac{P_{Y|X}\left(y_{i}|s_{i}\right)}{P_{Y|S}\left(y_{i}|s_{i}\right)}\right)\geq n\theta \right). \end{array}$$

If ${\tilde{\Pi }}_{\theta }\left(y\right)=1$, then $\hat{M}=0$. Else, for a given $B_{X,n}$, maximum likelihood (ML) decoding is done on the remaining set of codewords {x(m),m∈M∖{0}}, and $\hat{M}$ is set equal to the ML estimate. Denote the channel decoder induced by the above operations by $g_{B_{X,n}}$, where $g_{B_{X,n}}:Y^{n}\to M$.

For a given codebook $B_{X,n}$, the channel encoder–decoder pair described above induces a distribution

$$\begin{array}{c} P_{XY\hat{M}|M}^{\left(B_{X,n}\right)}\left(m,x,y,\hat{m}|m\right):={𝟙}_{\left\{f_{B_{X,n}}\left(m\right)=x\right\}}\;P_{Y|X}^{⊗n}\left(y|x\right){𝟙}_{\left\{\hat{m}=g_{B_{X,n}}\right\}}. \end{array}$$

Note that $P_{x(0)x(m)}={\hat{P}}_{SX}$, $Y∼\prod_{i=1}^{\left|X\right|}P_{Y|X}^{⊗\lceil P_{S}\left(i\right)n\rceil }\left(\cdot |i\right)$ for M=0 and $Y∼\prod_{i=1}^{\left|X\right|}P_{Y|S}^{⊗\lceil P_{S}\left(i\right)n\rceil }\left(\cdot |i\right)$ for M=m≠0. Then, it follows by an application of Proposition A1 proved in Appendix C that for any $B_{X,n}$ and *n* sufficiently large, the Neyman–Pearson test in (20) yields

(21a) $$\begin{array}{cc} \;\;\;\;P_{P^{\left(B_{X,n}\right)}}\left(\hat{M}=0|M=m\right) & \leq e^{-n\left(E_{\mathrm{sp}}\left(P_{SX},\theta \right)-\eta \right)},\;m\in M\setminus \left\{0\right\}, \end{array}$$

(21b) $$\begin{array}{cc} P_{P^{\left(B_{X,n}\right)}}\left(\hat{M}\neq 0|M=0\right) & \leq e^{-n\left(E_{\mathrm{sp}}\left(P_{SX},\theta \right)-\theta -\eta \right)}. \end{array}$$

Moreover, given $\hat{M}\neq 0$, a random coding argument over the ensemble of $B_{X}^{n}$ (see Exercise 10.18, 10.24 in [38,44]) shows that there exists a deterministic codebook $B_{X,n}$ such that (21a) and (21b) holds, and the ML decoding described above asymptotically achieves

(22) $$\begin{array}{cc} P_{P^{\left(B_{X,n}\right)}}\left(\hat{M}\neq m|M=m\neq 0,\hat{M}\neq 0\right) & \leq e^{-n\left(E_{\mathrm{ex}}\left(R,P_{SX}\right)-\eta \right)}. \end{array}$$

This deterministic codebook $B_{X,n}$ is used for channel coding.

**Source decoder:** For a given codebook $B_{W,n}$ and inputs $\hat{M}=\hat{m}$ and V=v, the source decoder first decodes for the quantization codeword $w({\hat{m}}^{\prime })$ (if required) using the empirical conditional entropy decoder, and then declares the output $\hat{H}$ of the hypothesis test based on $w({\hat{m}}^{\prime })$ and v. More specifically, if binning is not performed, i.e., if $\left|M|\geq \right|M^{\prime }|$, ${\hat{M}}^{\prime }=\hat{m}$. Otherwise, ${\hat{M}}^{\prime }={\hat{m}}^{\prime }$, where ${\hat{m}}^{\prime }=0$ if $\hat{m}=0$ and ${\hat{m}}^{\prime }={arg min}_{j:f_{b}\left(j\right)=\hat{m}}\;H_{e}\left(w\left(j\right)|v\right)$ otherwise. Denote the source decoder induced by the above operations by $g_{B_{W,n}}:M\times V^{n}\to M^{\prime }$.

**Testing and Acceptance region:** If ${\hat{m}}^{\prime }=0$, $\hat{H}=1$ is declared. Otherwise, $\hat{H}=0$ or $\hat{H}=1$ is declared depending on whether $\left({\hat{m}}^{\prime },v\right)\in A_{n}$ or $\left({\hat{m}}^{\prime },v\right)\notin A_{n}$, respectively, where $A_{n}$ denotes the acceptance region for $H_{0}$ as specified next. For a given codebook $B_{W,n}$, let $O_{m^{\prime }}$ denote the set of u such that the source encoder outputs $m^{\prime }$, $m^{\prime }\in M^{\prime }\setminus \left\{0\right\}$. For each $m^{\prime }\in M^{\prime }\setminus \left\{0\right\}$ and $u\in O_{m^{\prime }}$, let

$$\begin{array}{c} Z_{m^{\prime }}\left(u\right)=\left\{v\in V^{n}:\left(w\left(m^{\prime }\right),u,v\right)\in J_{n}\left(\kappa_{\alpha }+\eta ,P_{W_{m^{\prime }}UV}\right)\right\}, \end{array}$$

where $J_{n}\left(r,P_{X}\right):=\left\{x\in X^{n}:D\left(P_{x}\left|\right|P_{X}\right)\leq r\right\}$,

(23) $$\begin{array}{c} P_{UVW_{m^{\prime }}}:=P_{UV}P_{W_{m^{\prime }}|U}\;\mathrm{and}\;P_{W_{m^{\prime }}|U}=\omega \left(P_{u}\right). \end{array}$$

For $m^{\prime }\in M^{\prime }\setminus \left\{0\right\}$, set $Z_{m^{\prime }}:=\left\{v:v\in Z_{m^{\prime }}\left(u\right)\mathrm{for}\;\mathrm{some}u\in O_{m^{\prime }}\right\}$, and define the acceptance region for $H_{0}$ at the decision maker as $A_{n}:=\cup_{m^{\prime }\in M^{\prime }\setminus 0}\;m^{\prime }\times Z_{m^{\prime }}$ or equivalently as $A_{n}^{e}:=\cup_{m^{\prime }\in M^{\prime }\setminus 0}O_{m^{\prime }}\times Z_{m^{\prime }}$. Note that $A_{n}$ is the same as the acceptance region for $H_{0}$ in Theorem 1 in [14]. Denote the decision function induced by $g_{B_{X,n}}$, $g_{B_{W,n}}$ and $A_{n}$ by $g_{n}:Y^{n}\times V^{n}\to \hat{H}$.

**Induced probability distribution:** The PMFs induced by a code $c_{n}=\left(f_{n},g_{n}\right)$ with respect to codebook $B_{n}:=\left(B_{W,n},f_{b},B_{X,n}\right)$ under $H_{0}$ and $H_{1}$ are

$$\begin{array}{cc} \; & P_{\mathrm{UV}M^{\prime }M\mathrm{XY}\hat{M}{\hat{M}}^{\prime }\hat{H}}^{\left(B_{n},c_{n}\right)}\left(u,v,m^{\prime },m,x,y,\hat{m},{\hat{m}}^{\prime },\hat{h}\right)\\ \; & :=P_{UV}^{⊗n}\left(u,v\right)\;{𝟙}_{\left\{M^{\prime }\left(u,B_{W,n}\right)=m^{\prime },\;f_{b}\left(m^{\prime }\right)=m\right\}}P_{XY\hat{M}|M}^{\left(B_{X,n}\right)}\left(x,y,\hat{m}|m\right)\;{𝟙}_{\left\{g_{B_{W,n}}\left(m,v\right)={\hat{m}}^{\prime },\hat{h}={𝟙}_{\left\{\left({\hat{m}}^{\prime },v\right)\in A_{n}^{c}\right\}}\right\}},\\ & Q_{\mathrm{UV}M^{\prime }M\mathrm{XY}\hat{M}{\hat{M}}^{\prime }\hat{H}}^{\left(B_{n},c_{n}\right)}\left(u,v,m^{\prime },m,x,y,\hat{m},{\hat{m}}^{\prime },\hat{h}\right)\\ \; & :=Q_{UV}^{⊗n}\left(u,v\right)\;{𝟙}_{\left\{M^{\prime }\left(u,B_{W,n}\right)=m^{\prime },f_{b}\left(m^{\prime }\right)=m\right\}}P_{XY\hat{M}|M}^{\left(B_{X,n}\right)}\left(x,y,\hat{m}|m\right)\;{𝟙}_{\left\{g_{B_{W,n}}\left(m,v\right)={\hat{m}}^{\prime },\hat{h}={𝟙}_{\left\{\left({\hat{m}}^{\prime },v\right)\in A_{n}^{c}\right\}}\right\}}, \end{array}$$

respectively. For simplicity, we will denote the above distributions by $P^{\left(B_{n}\right)}$ and $Q^{\left(B_{n}\right)}$. Let $B_{n}:=\left(B_{W,n},f_{B},B_{X,n}\right)$, $B_{n}$, and $\mu_{n}$ denote the random codebook, its support, and the probability measure induced by its random construction, respectively. Additionally, define ${\bar{P}}_{P^{\left(B_{n}\right)}}:=E_{\mu_{n}}\left[P_{P^{\left(B_{n}\right)}}\right]$ and ${\bar{P}}_{Q^{\left(B_{n}\right)}}:=E_{\mu_{n}}\left[P_{Q^{\left(B_{n}\right)}}\right]$.

**Analysis of the type I and type II error probabilities:** We analyze the type I and type II error probabilities averaged over the random ensemble of quantization and binning codebooks $\left(B_{W},f_{B}\right)$. Then, an expurgation technique [44] guarantees the existence of a sequence of deterministic codebooks ${\left\{B_{n}\right\}}_{n\in N}$ and a code ${\left\{c_{n}=\left(f_{n},g_{n}\right)\right\}}_{n\in N}$ that achieves the lower bound given in Theorem 1.

**Type I error probability:** In the following, random sets where the randomness is induced due to $B_{n}$ will be written using blackboard bold letters, e.g., $A_{n}$ for the random acceptance region for $H_{0}$. Note that a type I error can occur only under the following events:

(i) $E_{\mathrm{EE}}:=\overset{⋃}{P_{\hat{U}}\in D_{n}\left(P_{U},\eta \right)}\;\overset{⋃}{u\in T_{n}\left(P_{\hat{U}}\right)}\;E_{\mathrm{EE}}\left(u\right),\mathrm{where}$
  $\begin{array}{ccc} E_{\mathrm{EE}}\left(u\right) & :=\{∄\;j\in M^{\prime }\setminus \left\{0\right\}s.t.\left(u,W\left(j\right)\right)\in T_{n}\left(P_{{\hat{U}}_{i}{\hat{W}}_{i}}\right),P_{{\hat{U}}_{i}}=P_{u},\\ & \;\;\;\;\;\;P_{{\hat{U}}_{i}{\hat{W}}_{i}}\in D_{n}\left(P_{UW},\eta \right)\}, & \end{array}$
(ii) $E_{\mathrm{NE}}:=\left\{{\hat{M}}^{\prime }=M^{\prime }\;\mathrm{and}\;\left({\hat{M}}^{\prime },V\right)\notin A_{n}\right\},$
(iii) $E_{\mathrm{OCE}}:=\left\{M^{\prime }\neq 0,\hat{M}\neq M\;\mathrm{and}\;\left({\hat{M}}^{\prime },V\right)\notin A_{n}\right\},$
(iv) $E_{\mathrm{SCE}}:=\left\{M^{\prime }=M=0,\hat{M}\neq M\;\mathrm{and}\;\left({\hat{M}}^{\prime },V\right)\notin A_{n}\right\},$
(v) $E_{\mathrm{BE}}:=\left\{M^{\prime }\neq 0,\;\hat{M}=M,{\hat{M}}^{\prime }\neq M^{\prime }\;\mathrm{and}\;\left({\hat{M}}^{\prime },V\right)\notin A_{n}\right\}$.

Here, $E_{\mathrm{EE}}$ corresponds to the event that there does not exist a quantization codeword corresponding to atleast one sequence u of type $P_{u}\in D_{n}\left(P_{U},\eta \right)$; $E_{\mathrm{NE}}$ corresponds to the event, in which, there is neither an error at the channel decoder nor at the empirical conditional entropy decoder; $E_{\mathrm{OCE}}$ and $E_{\mathrm{SCE}}$ corresponds to the case, in which there is an error at the channel decoder (hence also at the empirical conditional entropy decoder); and $E_{\mathrm{BE}}$ corresponds to the case that there is an error (due to binning) only at the empirical conditional entropy decoder. For the event $E_{\mathrm{EE}}$, it follows from a slight generalization of the type-covering lemma (Lemma 9.1 in [38]) that

(24) $$\begin{array}{c} {\bar{P}}_{P^{\left(B_{n}\right)}}\left(E_{\mathrm{EE}}\right)\leq e^{-e^{n\Omega (\eta )}}. \end{array}$$

Since $e^{n\Omega (\eta )}/n\overset{\left(n\right)}{\to }\infty$ for η>0, the event $E_{\mathrm{EE}}$ may be safely ignored from the analysis of the error-exponents. Given that $E_{\mathrm{EE}}^{c}$ holds for some $B_{W,n}$, it follows from Equation 4.22 in [14] that

(25) $$\begin{array}{c} {\bar{P}}_{P^{\left(B_{n}\right)}}\left(E_{\mathrm{NE}}|E_{\mathrm{EE}}^{c}\right)\leq e^{-n\kappa_{\alpha }}, \end{array}$$

for sufficiently large *n* since the acceptance region is the same as that in Theorem 1 in [14].

Next, consider the event $E_{\mathrm{OCE}}$. We have for sufficiently large *n* that

(26) $$\begin{array}{ccc} {\bar{P}}_{P^{\left(B_{n}\right)}}\left(E_{\mathrm{OCE}}\right) & \leq {\bar{P}}_{P^{\left(B_{n}\right)}}\left(M^{\prime }\neq 0\right){\bar{P}}_{P^{\left(B_{n}\right)}}\left(\hat{M}\neq M|M^{\prime }\neq 0\right)\\ & \overset{\left(a\right)}{\leq }{\bar{P}}_{P^{\left(B_{n}\right)}}\left(\hat{M}\neq M|M\neq 0\right)\\ & \leq {\bar{P}}_{P^{\left(B_{n}\right)}}\left(\hat{M}=0|M\neq 0\right)+{\bar{P}}_{P^{\left(B_{n}\right)}}\left(\hat{M}\neq M|M\neq 0,\hat{M}\neq 0\right)\\ & \overset{\left(b\right)}{\leq }e^{-n\left(E_{m}\left(P_{SX},\theta \right)-\eta \right)}+e^{-n\left(E_{\mathrm{ex}}\left(R,P_{SX}\right)-\eta \right)}\\ & =e^{-n\left(\min\left\{E_{m}\left(P_{SX},\theta \right),E_{\mathrm{ex}}\left(R,P_{SX}\right)\right\}-\eta \right)}, & \end{array}$$

where

(a) holds since the event $\left\{M^{\prime }\neq 0\right\}$ is equivalent to {M≠0};
(b) holds due to (21a) and (22), which holds for $B_{X,n}$.

Additionally, the probability of $E_{\mathrm{SCE}}$ can be upper bounded as

(27) $$\begin{array}{ccc} {\bar{P}}_{P^{\left(B_{n}\right)}}\left(E_{\mathrm{SCE}}\right) & \leq {\bar{P}}_{P^{\left(B_{n}\right)}}\left(M^{\prime }=0\right)\\ & \leq {\bar{P}}_{P^{\left(B_{n}\right)}}\left(M^{\prime }=0|U\in D_{n}\left(P_{U},\eta \right)\right)+{\bar{P}}_{P^{\left(B_{n}\right)}}\left(U\notin D_{n}\left(P_{U},\eta \right)\right)\\ & ={\bar{P}}_{P^{\left(B_{n}\right)}}\left(E_{\mathrm{EE}}\right)+{\bar{P}}_{P^{\left(B_{n}\right)}}\left(U\notin D_{n}\left(P_{U},\eta \right)\right)\\ & \leq e^{-n\kappa_{\alpha }}, & \end{array}$$

where (27) is due to (24), the definition of $D_{n}\left(P_{U},\eta \right)$ in (15) and Lemma 2.2 and Lemma 2.6 in [38].

Finally, consider the event $E_{\mathrm{BE}}$. Note that this event occurs only when $\left|M|\leq \right|M^{\prime }|$. Additionally, M=0 iff $M^{\prime }=0$, and hence $M^{\prime }\neq 0$ and $\hat{M}=M$ implies that $\hat{M}\neq 0$. Let

$$\begin{array}{cc} D_{n}\left(P_{VW},\eta \right)\; & :=\left\{P_{\hat{V}\hat{W}}:\begin{array}{cc} & \exists \;\left(w,u,v\right)\in \underset{m^{\prime }\in M^{\prime }\setminus \left\{0\right\}}{\cup }J_{n}\left(\kappa_{\alpha }+\eta ,P_{W_{m^{\prime }}UV}\right),P_{W_{m^{\prime }}UV}\mathrm{satisfies}\\ \; & \left(23\right)\;\mathrm{and}\;P_{wuv}=P_{\hat{W}\hat{U}\hat{V}} \end{array}\right\}. \end{array}$$

We have

(28) $$\begin{array}{cc} {\bar{P}}_{P^{\left(B_{n}\right)}}\left(E_{\mathrm{BE}}\right) & ={\bar{P}}_{P^{\left(B_{n}\right)}}\left(E_{\mathrm{BE}},\left(M^{\prime },V\right)\in A_{n}\right)+{\bar{P}}_{P^{\left(B_{n}\right)}}\left(E_{\mathrm{BE}},\left(M^{\prime },V\right)\notin A_{n}\right). \end{array}$$

The second term in (28) can be upper bounded as

(29) $$\begin{array}{ccc} {\bar{P}}_{P^{\left(B_{n}\right)}}\left(E_{\mathrm{BE}},\left(M^{\prime },V\right)\notin A_{n}\right) & \leq {\bar{P}}_{P^{\left(B_{n}\right)}}\left(\left(M^{\prime },V\right)\notin A_{n},E_{\mathrm{EE}}\right)+{\bar{P}}_{P^{\left(B_{n}\right)}}\left(\left(M^{\prime },V\right)\notin A_{n},E_{\mathrm{EE}}^{c}\right)\\ & \leq e^{-e^{n\Omega (\eta )}}+{\bar{P}}_{P^{\left(B_{n}\right)}}\left(\left(M^{\prime },V\right)\notin A_{n}|E_{\mathrm{EE}}^{c}\right)\\ & \leq e^{-e^{n\Omega (\eta )}}+{\bar{P}}_{P^{\left(B_{n}\right)}}\left(\left(U,V\right)\notin A_{n}^{e}\right)\\ & \leq e^{-e^{n\Omega (\eta )}}+e^{-n\kappa_{\alpha }}, & \end{array}$$

where the inequality in (29) follows from Equation (4.22) in [14] for sufficiently large *n* since the acceptance region $A_{n}^{e}$ is the same as that in [14]. To bound the first term in (28), define $D_{n}\left(P_{V},\eta \right):=\left\{P_{\hat{V}}:\exists \;P_{\hat{V}\hat{W}}\in D_{n}\left(P_{VW},\eta \right)\right\}$, and observe that since $\left(M^{\prime },V\right)\in A_{n}$ implies $M^{\prime }\neq 0$, we have

(30) $$\begin{array}{cc} & {\bar{P}}_{P^{\left(B_{n}\right)}}\left(E_{\mathrm{BE}},\left(M^{\prime },V\right)\in A_{n}\right)\\ & =\sum_{\left(m^{\prime },m\right)\in M^{\prime }\times M}{\bar{P}}_{P^{\left(B_{n}\right)}}\left(E_{\mathrm{BE}},\left(M^{\prime },V\right)\in A_{n},M=m,M^{\prime }=m^{\prime }\right)\\ & =\sum_{\left(m^{\prime },m\right)\in M^{\prime }\times M}{\bar{P}}_{P^{\left(B_{n}\right)}}\left(M=m,M^{\prime }=m^{\prime },\hat{M}=M\right)\;\\ & \;\;\;\;{\bar{P}}_{P^{\left(B_{n}\right)}}\left({\hat{M}}^{\prime }\neq M^{\prime },\left({\hat{M}}^{\prime },V\right)\notin A_{n},\left(M^{\prime },V\right)\in A_{n}|M^{\prime }=m^{\prime },M=m,\hat{M}=M\right)\\ & \leq \sum_{\left(m^{\prime },m\right)\in M^{\prime }\times M}{\bar{P}}_{P^{\left(B_{n}\right)}}\left(M=m,M^{\prime }=m^{\prime },\hat{M}=M\right)\;\\ & \;\;\;\;{\bar{P}}_{P^{\left(B_{n}\right)}}\left({\hat{M}}^{\prime }\neq M^{\prime },\left(M^{\prime },V\right)\in A_{n}|M^{\prime }=m^{\prime },M=m,\hat{M}=M\right)\\ & \overset{\left(a\right)}{=}{\bar{P}}_{P^{\left(B_{n}\right)}}\left({\hat{M}}^{\prime }\neq M^{\prime },\left(M^{\prime },V\right)\in A_{n}|M^{\prime }=1,M=1,\hat{M}=M\right)\\ & \overset{\left(b\right)}{\leq }\sum_{\begin{array}{c} P_{v}\in D_{n}\left(P_{V},\eta \right) \end{array}}\sum_{v\in P_{v}}{\bar{P}}_{P^{\left(B_{n}\right)}}\left(V=v|M^{\prime }=1\right) \end{array}$$

(31) $$\begin{array}{ccc} & \;\;{\bar{P}}_{P^{\left(B_{n}\right)}}\left(\exists \;j\in f_{B}^{-1}\left(1\right),\;j\neq 1,H_{e}\left(W\left(j\right)|v\right)\leq H_{e}\left(W\left(1\right)|v\right)|M^{\prime }=1,V=v\right), & \end{array}$$

where (a) follows since by the symmetry of the source encoder, binning function and random codebook construction, the term in (30) is independent of $\left(m,m^{\prime }\right)$; (b) holds since $\left(M^{\prime },V\right)\in A_{n}$ implies that $P_{v}\in D_{n}\left(P_{V},\eta \right)$ and $\left(V,B_{W}\right)-M^{\prime }-\left(M,\hat{M}\right)$ form a Markov chain. Defining $P_{\hat{V}}=P_{v}$, and the event $E_{1}^{\prime }:=\left\{M^{\prime }=1,V=v\right\}$, we obtain

(32) $$\begin{array}{cc} & {\bar{P}}_{P^{\left(B_{n}\right)}}\left(\exists \;j\in f_{B}^{-1}\left(1\right),\;j\neq 1,H_{e}\left(W\left(j\right)|v\right)\leq H_{e}\left(W\left(1\right)|v\right)\;|\;E_{1}^{\prime }\right)\\ & =\sum_{j\in M^{\prime }\setminus \left\{0,1\right\}}{\bar{P}}_{P^{\left(B_{n}\right)}}\left(f_{B}\left(j\right)=1,H_{e}\left(W\left(j\right)|v\right)\leq H_{e}\left(W\left(1\right)|v\right)\;|\;E_{1}^{\prime }\right)\\ & \overset{\left(a\right)}{\leq }\frac{1}{e^{nR_{n}}}\sum_{j\in M^{\prime }\setminus \left\{0,1\right\}}{\bar{P}}_{P^{\left(B_{n}\right)}}\left(H_{e}\left(W\left(j\right)|v\right)\leq H_{e}\left(W\left(1\right)|v\right)\;|\;E_{1}^{\prime }\right)\\ & \overset{\left(b\right)}{\leq }\frac{1}{e^{nR_{n}}}\sum_{j\in M^{\prime }\setminus \left\{0,1\right\}}\;\sum_{\begin{array}{c} P_{\hat{W}}:P_{\hat{V}\hat{W}}\in D_{n}\left(P_{VW},\eta \right) \end{array}}\sum_{\begin{array}{c} w:\left(v,w\right)\in T_{n}\left(P_{\hat{V}\hat{W}}\right) \end{array}}{\bar{P}}_{P^{\left(B_{n}\right)}}\left(W\left(1\right)=w\;|\;E_{1}^{\prime }\right)\;\\ & \;\;\;\;\;\sum_{\begin{array}{c} \tilde{w}\in T_{n}\left(P_{\hat{W}}\right):H_{e}\left(\tilde{w}|v\right)\leq H\left(\hat{W}|\hat{V}\right) \end{array}}{\bar{P}}_{P^{\left(B_{n}\right)}}\left(W\left(j\right)=\tilde{w}\;|\;E_{1}^{\prime }\cup \left\{W\left(1\right)=w\right\}\right)\\ & \overset{\left(c\right)}{\leq }\frac{1}{e^{nR_{n}}}\sum_{j\in M^{\prime }\setminus \left\{0,1\right\}}\;\sum_{\begin{array}{c} P_{\hat{W}}:P_{\hat{V}\hat{W}}\in D_{n}\left(P_{VW},\eta \right) \end{array}}\sum_{\begin{array}{c} w:\left(v,w\right)\in T_{n}\left(P_{\hat{V}\hat{W}}\right) \end{array}}{\bar{P}}_{P^{\left(B_{n}\right)}}\left(W\left(1\right)=w\;|\;E_{1}^{\prime }\right)\\ & \;\;\;\;\;\;\;\;\sum_{\begin{array}{c} \tilde{w}\in T_{n}\left(P_{\hat{W}}\right):H_{e}\left(\tilde{w}|v\right)\leq H\left(\hat{W}|\hat{V}\right) \end{array}}2\;{\bar{P}}_{P^{\left(B_{n}\right)}}\left(W\left(j\right)=\tilde{w}\right), \end{array}$$

where

(a) follows since $f_{B}\left(\cdot \right)$ is the uniform binning function independent of $B_{W,n}$;
(b) holds due to the fact that if $P_{v}\in D_{n}\left(P_{V},\eta \right)$, then $M^{\prime }=1$ implies that $\left(W\left(1\right),v\right)\in T_{n}\left(P_{\hat{V}\hat{W}}\right)$ with probability one for some $P_{\hat{V}\hat{W}}\in D_{n}\left(P_{VW},\eta \right)$;
(c) holds since ${\bar{P}}_{P^{\left(B_{n}\right)}}\left(W\left(j\right)=\tilde{w}\;|\;E_{1}^{\prime }\cup \left\{W\left(1\right)=w\right\}\right)\leq 2\;{\bar{P}}_{P^{\left(B_{n}\right)}}\left(W\left(j\right)=\tilde{w}\right)$, which follows similarly to Equation (101) in [10].

Continuing, we can write for sufficiently large *n*,

(33) $$\begin{array}{cc} & {\bar{P}}_{P^{\left(B_{n}\right)}}\left(\exists \;j\in f_{B}^{-1}\left(1\right),\;j\neq 1,H_{e}\left(W\left(j\right)|v\right)\leq H_{e}\left(W\left(1\right)|v\right)\;|\;E_{1}^{\prime }\right)\\ & \overset{\left(a\right)}{\leq }\frac{1}{e^{nR_{n}}}\sum_{j\in M^{\prime }\setminus \left\{0,1\right\}}\;\sum_{\begin{array}{c} P_{\hat{W}}:P_{\hat{V}\hat{W}}\in D_{n}\left(P_{VW},\eta \right) \end{array}}\sum_{\begin{array}{c} w:\left(v,w\right)\in T_{n}\left(P_{\hat{V}\hat{W}}\right) \end{array}}{\bar{P}}_{P^{\left(B_{n}\right)}}\left(W\left(1\right)=w\;|\;E_{1}^{\prime }\right)\\ & \;\;\;\;\;\;\sum_{\begin{array}{c} \tilde{w}\in T_{n}\left(P_{\hat{W}}\right):H_{e}\left(\tilde{w}|v\right)\leq H\left(\hat{W}|\hat{V}\right) \end{array}}2\;e^{-n(H\left(\hat{W}\right)-\eta )}\\ & \overset{\left(b\right)}{\leq }\frac{1}{e^{nR_{n}}}\sum_{j\in M^{\prime }\setminus \left\{0,1\right\}}\;\sum_{\begin{array}{c} P_{\hat{W}}:P_{\hat{V}\hat{W}}\in D_{n}\left(P_{VW},\eta \right) \end{array}}\sum_{\begin{array}{c} w:\left(v,w\right)\in T_{n}\left(P_{\hat{V}\hat{W}}\right) \end{array}}{\bar{P}}_{P^{\left(B_{n}\right)}}\left(W\left(1\right)=w\;|\;E_{1}^{\prime }\right)\;\\ & \;\;\;\;\;\;\;\;{\left(n+1\right)}^{\left|V||W\right|}e^{nH(\hat{W}|\hat{V})}2\;e^{-n(H\left(\hat{W}\right)-\eta )}\\ & \leq \frac{1}{e^{nR_{n}}}\sum_{j\in M^{\prime }\setminus \left\{0,1\right\}}\;\sum_{\begin{array}{c} P_{\hat{W}}:P_{\hat{V}\hat{W}}\in D_{n}\left(P_{VW},\eta \right) \end{array}}2\;{\left(n+1\right)}^{\left|V||W\right|}\;e^{-n\left(I(\hat{W};\hat{V})-\eta \right)}\\ & \overset{\left(c\right)}{\leq }\frac{1}{e^{nR_{n}}}\sum_{j\in M^{\prime }\setminus \left\{0,1\right\}}2\;{\left(n+1\right)}^{\left|W\right|}\;{\left(n+1\right)}^{\left|V||W\right|}\;e^{-n\left(\min_{P_{\hat{V}\hat{W}}\in D_{n}\left(P_{VW},\eta \right)}I\left(\hat{W};\hat{V}\right)-\eta \right)}\\ & \overset{\left(d\right)}{\leq }e^{-n(R-R^{\prime }+\rho_{n}-\eta_{n}^{\prime })}, \end{array}$$

where $\rho_{n}:=\min_{P_{\hat{V}\hat{W}}\in D_{n}\left(P_{VW},\eta \right)}I\left(\hat{V};\hat{W}\right)$ and $\eta_{n}^{\prime }:=3\eta +o\left(1\right)$. In the above,

*(a)*  used Lemma 2.3 in [38] and the fact that the codewords are chosen uniformly at random from $T_{n}\left(P_{\hat{W}}\right)$;
*(b)*  follows since the total number of sequences $\tilde{w}\in T_{n}\left(P_{\hat{W}}\right)$ such that $P_{\tilde{w}v}=P_{\tilde{W}\tilde{V}}$ and $H\left(\tilde{W}|\tilde{V}\right)\leq H\left(\hat{W}|\hat{V}\right)$ is upper bounded by $e^{nH(\hat{W}|\hat{V})}$, and $|T\left(W^{n}\times V^{n}\right)|\leq {\left(n+1\right)}^{\left|V||W\right|}$;
*(c)*  holds due to Lemma 2.2 in [38];
*(d)*  follows from $R^{\prime }:=\zeta (\kappa_{\alpha }$, (14), (18) and (19).

Thus, since $\rho_{n}\to \rho \left(\kappa_{\alpha },\omega \right)+O\left(\eta \right)$, we have from (28), (29), (31), (33) for large enough *n* that

(34) $$\begin{array}{c} {\bar{P}}_{P^{\left(B_{n}\right)}}\left(E_{\mathrm{BE}}\right)\leq e^{-n\left(\min\left\{\kappa_{\alpha },R-\zeta \left(\kappa_{\alpha },\omega \right)+\rho \left(\kappa_{\alpha },\omega \right)-O\left(\eta \right)\right\}\right)}. \end{array}$$

By choice of $\left(\omega ,P_{SX},\theta \right)\in L\left(\kappa_{\alpha }\right)$, it follows from (24), (25), (26), (27) and (34) that the type I error probability is upper bounded by $e^{-n\left(\kappa_{\alpha }-O\left(\eta \right)\right)}$ for large *n*.

**Type II error probability:** We analyze the type II error probability averaged over $B_{n}$. A type II error can occur only under the following events:

(i) $E_{a}:=\left\{\begin{array}{cc} & \hat{M}=M,{\hat{M}}^{\prime }=M^{\prime }\neq 0,\left(U,V,W\left(M^{\prime }\right)\right)\in T_{n}\left(P_{\hat{U}\hat{V}\hat{W}}\right)\\ & s.t.P_{\hat{U}\hat{W}}\in D_{n}\left(P_{UW},\eta \right)\;\mathrm{and}\;P_{\hat{V}\hat{W}}\in D_{n}\left(P_{VW},\eta \right) \end{array}\right\},$
(ii) $E_{b}:=\left\{\begin{array}{cc} & M^{\prime }\neq 0,\hat{M}=M,{\hat{M}}^{\prime }\neq M^{\prime },f_{B}\left({\hat{M}}^{\prime }\right)=f_{B}\left(M^{\prime }\right),(U,V,W\left(M^{\prime }\right),\\ & W\left({\hat{M}}^{\prime }\right))\in T_{n}\left(P_{\hat{U}\hat{V}\hat{W}{\hat{W}}_{d}}\right)s.t.P_{\hat{U}\hat{W}}\in D_{n}\left(P_{UW},\eta \right),\\ & P_{\hat{V}{\hat{W}}_{d}}\in D_{n}\left(P_{VW},\eta \right)\;\mathrm{and}\;H_{e}\left(W({\hat{M}}^{\prime })|V\right)\leq H_{e}\left(W(M^{\prime })|V\right) \end{array}\right\},$
(iii) $E_{c}:=\left\{\begin{array}{cc} & M^{\prime }\neq 0,\hat{M}\neq M\;or\;0,\left(U,V,W\left(M^{\prime }\right),W\left({\hat{M}}^{\prime }\right)\right)\in T_{n}\left(P_{\hat{U}\hat{V}\hat{W}{\hat{W}}_{d}}\right)s.t.\\ & P_{\hat{U}\hat{W}}\in D_{n}\left(P_{UW},\eta \right)\;\mathrm{and}\;P_{\hat{V}{\hat{W}}_{d}}\in D_{n}\left(P_{VW},\eta \right) \end{array}\right\},$
(iv) $E_{d}:=\left\{M=M^{\prime }=0,\hat{M}\neq M,\left(V,W\left({\hat{M}}^{\prime }\right)\right)\in T_{n}\left(P_{\hat{V}{\hat{W}}_{d}}\right)s.t.P_{\hat{V}{\hat{W}}_{d}}\in D_{n}\left(P_{VW},\eta \right)\right\}.$

Similar to (24), it follows that ${\bar{P}}_{Q^{\left(B_{n}\right)}}\left(E_{\mathrm{EE}}\right)\leq e^{-e^{n\Omega (\eta )}}$. Hence, we may assume that $E_{\mathrm{EE}}^{c}$ holds for the type II error-exponent analysis. It then follows from the analysis in Equations (4.23)–(4.27) in [14] that for sufficiently large *n*,

$$\begin{array}{c} {\bar{P}}_{Q^{\left(B_{n}\right)}}\left(E_{a}|E_{\mathrm{EE}}^{c}\right)\leq e^{-n\left(E_{1}\left(\kappa_{\alpha },\omega \right)-O\left(\eta \right)\right)}. \end{array}$$

The analysis of the error events $E_{b}$, $E_{c}$ and $E_{d}$ follows similarly to that in the proof of Theorem 2 in [10], and results in

$$\begin{array}{ccc} & -\frac{1}{n}\log\left({\bar{P}}_{Q^{\left(B_{n}\right)}}\left(E_{b}\right)\right)\\ & ≳\left\{\begin{array}{cc} \min_{\begin{array}{c} \left(P_{\tilde{U}\tilde{V}\tilde{W}},Q_{\tilde{U}\tilde{V}\tilde{W}}\right)\in T_{2}\left(\kappa_{\alpha },\omega \right) \end{array}}D(P_{\tilde{U}\tilde{V}\tilde{W}}\left|\right|Q_{\tilde{U}\tilde{V}\tilde{W}})+E_{b}\left(\kappa_{\alpha },\omega ,R\right)-O\left(\eta \right), & \mathrm{if}\;R<\zeta (\kappa_{\alpha },\omega )+\eta ,\\ \;\;\;\;\;\;\infty , & \mathrm{otherwise}, \end{array}\right.\\ & =E_{2}\left(\kappa_{\alpha },\omega ,R\right)-O\left(\eta \right).\\ & \frac{-1}{n}\log\left({\bar{P}}_{Q^{\left(B_{n}\right)}}\left(E_{c}\right)\right)\\ & ≳\left\{\begin{array}{cc} \min_{\begin{array}{c} \left(P_{\tilde{U}\tilde{V}\tilde{W}},Q_{\tilde{U}\tilde{V}\tilde{W}}\right)\in T_{3}\left(\kappa_{\alpha },\omega \right) \end{array}}D(P_{\tilde{U}\tilde{V}\tilde{W}}\left|\right|Q_{\tilde{U}\tilde{V}\tilde{W}})+E_{b}\left(\kappa_{\alpha },\omega ,R\right) & \mathrm{if}\;R<\zeta (\kappa_{\alpha },\omega )+\eta \\ \;\;\;\;\;\;\;\;+E_{\mathrm{ex}}\left(R,P_{SX}\right)-O\left(\eta \right), & \\ \min_{\begin{array}{c} \left(P_{\tilde{U}\tilde{V}\tilde{W}},Q_{\tilde{U}\tilde{V}\tilde{W}}\right)\in T_{3}\left(\kappa_{\alpha },\omega \right) \end{array}}D(P_{\tilde{U}\tilde{V}\tilde{W}}\left|\right|Q_{\tilde{U}\tilde{V}\tilde{W}})+\rho \left(\kappa_{\alpha },\omega \right) & \mathrm{otherwise},\\ \;\;\;\;\;\;\;\;+E_{\mathrm{ex}}\left(R,P_{SX}\right)-O\left(\eta \right), \end{array}\right.\\ & =E_{3}\left(\kappa_{\alpha },\omega ,R,P_{SX}\right)-O\left(\eta \right).\\ & \frac{-1}{n}\log\left({\bar{P}}_{Q^{\left(B_{n}\right)}}\left(E_{d}\right)\right)\\ & ≳\left\{\begin{array}{cc} \min_{P_{\tilde{V}}:P_{\tilde{V}\tilde{W}}\in D_{n}\left(P_{VW},\eta \right)}D(P_{\tilde{V}}\left|\right|Q_{V})+E_{b}\left(\kappa_{\alpha },\omega ,R\right) & \mathrm{if}\;R<\zeta (\kappa_{\alpha },\omega )+\eta ,\\ \;\;\;\;\;\;\;\;+E_{\mathrm{sp}}\left(P_{SX},\theta \right)-\theta -O\left(\eta \right), & \\ \min_{\begin{array}{c} P_{\tilde{V}}:P_{\tilde{V}\tilde{W}}\in D_{n}\left(P_{VW},\eta \right) \end{array}}D(P_{\tilde{V}}\left|\right|Q_{V})+\rho \left(\kappa_{\alpha },\omega \right) & \mathrm{otherwise},\\ \;\;\;\;\;\;\;\;+E_{\mathrm{sp}}\left(P_{SX},\theta \right)-\theta -O\left(\eta \right), \end{array}\right.\\ & =E_{4}\left(\kappa_{\alpha },\omega ,R,P_{SX},\theta \right)-O\left(\eta \right). & \end{array}$$

Since the exponent of the type II error probability is lower bounded by the minimum of the exponent of the type II error-causing events, we have shown from the above that for a fixed $\left(\omega ,R,P_{SX},\theta \right)\in L\left(\kappa_{\alpha }\right)$ and sufficiently large *n*,

(35a) $$\begin{array}{cc} {\bar{P}}_{P^{\left(B_{n}\right)}}\left(\hat{H}=1\right) & \leq e^{-n(\kappa_{\alpha }-O\left(\eta \right))}, \end{array}$$

(35b) $$\begin{array}{cc} \;\;\;\;\;{\bar{P}}_{Q^{\left(B_{n}\right)}}\left(\hat{H}=0\right) & \leq e^{-n({\bar{\kappa }}_{s}\left(\kappa_{\alpha },\omega ,R,P_{SX},\theta \right)-O\left(\eta \right))}, \end{array}$$

where

$$\begin{array}{c} {\bar{\kappa }}_{s}\left(\kappa_{\alpha },\omega ,R,P_{SX},\theta \right):=\min\left\{E_{1}\left(\kappa_{\alpha },\omega \right),E_{2}\left(\kappa_{\alpha },\omega ,R\right),E_{3}\left(\kappa_{\alpha },\omega ,R,P_{SX}\right),E_{4}\left(\kappa_{\alpha },\omega ,R,P_{SX},\theta \right)\right\}. \end{array}$$

**Expurgation:** To complete the proof, we extract a deterministic codebook $B_{n}^{★}$ that satisfies

$$\begin{array}{cc} P_{P^{\left(B_{n}^{★}\right)}}\left(\hat{H}=1\right) & \leq e^{-n(\kappa_{\alpha }-O\left(\eta \right))},\\ P_{Q^{\left(B_{n}^{★}\right)}}\left(\hat{H}=0\right) & \leq e^{-n({\bar{\kappa }}_{s}\left(\kappa_{\alpha },\omega ,R,P_{SX},\theta \right)-O\left(\eta \right))}. \end{array}$$

For this purpose, remove a set $B_{n}^{\prime }\subset B_{n}$ of highest type I error probability codebooks such that the remaining set $B_{n}\setminus B_{n}^{\prime }$ has a probability of τ∈(0.25,0.5), i.e., $\mu_{n}\left(B_{n}\setminus B_{n}^{\prime }\right)=\tau$. Then, it follows from (35a) and (35b) that for all $B_{n}\in B_{n}\setminus B_{n}^{\prime }$,

$$\begin{array}{cc} P_{P^{\left(B_{n}\right)}}\left(\hat{H}=1\right) & \leq 2e^{-n(\kappa_{\alpha }-O\left(\eta \right))},\\ {\tilde{P}}_{Q^{\left(B_{n}\right)}}\left(\hat{H}=0\right) & \leq 4e^{-n({\bar{\kappa }}_{s}\left(\kappa_{\alpha },\omega ,R,P_{SX},\theta \right)-O\left(\eta \right))}, \end{array}$$

where ${\tilde{P}}_{Q^{\left(B_{n}\right)}}=\frac{1}{\tau }E_{\mu_{n}}\left[P_{Q^{B_{n}}}{𝟙}_{\left\{B_{n}\in B_{n}\setminus B_{n}^{\prime }\right\}}\right]$ is a PMF. Perform one more similar expurgation step to obtain $B_{n}^{★}=\left(B_{W,n}^{★},f_{b}^{★},B_{X,n}^{★}\right)\in B_{n}\setminus B_{n}^{\prime }$ such that for all sufficiently large *n*

$$\begin{array}{cc} P_{P^{\left(B_{n}^{★}\right)}}\left(\hat{H}=1\right) & \leq 2e^{-n(\kappa_{\alpha }-O\left(\eta \right))}\leq e^{-n\left(\kappa_{\alpha }-O\left(\eta \right)-\left(\log2/n\right)\right)},\\ P_{Q^{\left(B_{n}^{★}\right)}}\left(\hat{H}=0\right) & \leq 4e^{-n\left({\bar{\kappa }}_{s}\left(\kappa_{\alpha },\omega ,R,P_{SX},\theta \right)-O\left(\eta \right)\right)}\leq e^{-n\left({\bar{\kappa }}_{s}\left(\kappa_{\alpha },\omega ,R,P_{SX},\theta \right)-O\left(\eta \right)-\left(\log4/n\right)\right)}. \end{array}$$

Maximizing over $\left(\omega ,R,P_{SX},\theta \right)\in L\left(\kappa_{\alpha }\right)$ and noting that η>0 is arbitrary completes the proof.

### 4.2. Proof of Corollary 1

Consider $\left(\omega ,P_{SX},\theta \right)\in L^{★}\left(\kappa_{\alpha }\right)$ and $R=\zeta (\kappa_{\alpha },\omega )$. Then, $\left(\omega ,R,P_{SX},\theta \right)\in L\left(\kappa_{\alpha }\right)$. Additionally, for any $\left(P_{\tilde{U}\tilde{V}\tilde{W}},Q_{\tilde{U}\tilde{V}\tilde{W}}\right)\in T_{1}\left(\kappa_{\alpha },\omega \right)$, we have

(36) $$\begin{array}{ccc} D(P_{\tilde{U}\tilde{V}\tilde{W}}||Q_{\tilde{U}\tilde{V}\tilde{W}}) & =D(P_{\tilde{U}\tilde{W}}\left|\right|Q_{\tilde{U}\tilde{W}})+D\left(P_{\tilde{V}|\tilde{U}\tilde{W}}\left|\right|Q_{\tilde{V}|\tilde{U}\tilde{W}}|P_{\tilde{U}\tilde{W}}\right)\\ & \overset{\left(a\right)}{\geq }D\left(P_{\tilde{V}|\tilde{U}\tilde{W}}\left|\right|P_{V}|P_{\tilde{U}\tilde{W}}\right)\\ & =D\left(P_{\tilde{V}\tilde{U}\tilde{W}}\left|\right|P_{V}P_{\tilde{U}\tilde{W}}\right)\\ & \overset{\left(b\right)}{\geq }D\left(P_{\tilde{V}\tilde{W}}\left|\right|P_{V}P_{\tilde{W}}\right)\\ & \overset{\left(c\right)}{=}D\left(P_{\hat{V}\hat{W}}\left|\right|P_{V}P_{\hat{W}}\right)\\ & =I_{P}\left(\hat{V};\hat{W}\right)+D(P_{\hat{V}}\left|\right|P_{V}), & \end{array}$$

where (a) is due to the non-negativity of KL divergence and since $Q_{\tilde{V}|\tilde{U}\tilde{W}}=P_{V}$; (b) is because of the monotonicity of KL divergence Theorem 2.14 in [43]; (c) follows since for $\left(P_{\tilde{U}\tilde{V}\tilde{W}},Q_{\tilde{U}\tilde{V}\tilde{W}}\right)\in T_{1}\left(\kappa_{\alpha },\omega \right)$, $P_{\tilde{V}\tilde{W}}=P_{\hat{V}\hat{W}}$ for some $P_{\hat{U}\hat{V}\hat{W}}\in \hat{L}\left(\kappa_{\alpha },\omega \right)$. Minimizing over all $P_{\hat{U}\hat{V}\hat{W}}\in \hat{L}\left(\kappa_{\alpha },\omega \right)$ yields that

$$\begin{array}{ccc} E_{1}\left(\kappa_{\alpha },\omega \right) & =\min_{\begin{array}{c} \left(P_{\tilde{U}\tilde{V}\tilde{W}},Q_{\tilde{U}\tilde{V}\tilde{W}}\right)\in T_{1}\left(\kappa_{\alpha },\omega \right) \end{array}}D(P_{\tilde{U}\tilde{V}\tilde{W}}\left|\right|Q_{\tilde{U}\tilde{V}\tilde{W}})\\ & \geq \min_{P_{\hat{U}\hat{V}\hat{W}}\in \hat{L}\left(\kappa_{\alpha },\omega \right)}\left[I_{P}\left(\hat{V};\hat{W}\right)+D(P_{\hat{V}}\left|\right|P_{V})\right]\\ & =\min_{\begin{array}{c} P_{\hat{V}\hat{W}}:P_{\hat{U}\hat{V}\hat{W}}\in \hat{L}\left(\kappa_{\alpha },\omega \right) \end{array}}\left[I_{P}\left(\hat{V};\hat{W}\right)+D(P_{\hat{V}}\left|\right|P_{V})\right]:=E_{1}^{i}\left(\kappa_{\alpha },\omega \right), & \end{array}$$

where the inequality above follows from (36). Next, since $\zeta (\kappa_{\alpha },\omega )=R$, we have that $E_{2}\left(\kappa_{\alpha },\omega ,R\right)=\infty$. Additionally, by the non-negativity of KL divergence

$$\begin{array}{ccc} E_{3}\left(\kappa_{\alpha },\omega ,R,P_{SX}\right) & =\min_{\begin{array}{c} \left(P_{\tilde{U}\tilde{V}\tilde{W}},Q_{\tilde{U}\tilde{V}\tilde{W}}\right)\\ \in T_{3}\left(\kappa_{\alpha },\omega \right) \end{array}}D(P_{\tilde{U}\tilde{V}\tilde{W}}\left|\right|Q_{\tilde{U}\tilde{V}\tilde{W}})+\rho \left(\kappa_{\alpha },\omega \right)+E_{\mathrm{ex}}\left(R,P_{SX}\right)\\ & \geq \rho \left(\kappa_{\alpha },\omega \right)+E_{\mathrm{ex}}\left(\zeta \left(\kappa_{\alpha },\omega \right),P_{SX}\right):=E_{2}^{i}\left(\kappa_{\alpha },\omega ,P_{SX}\right),\\ E_{4}\left(\kappa_{\alpha },\omega ,P_{SX},\theta \right) & =\min_{P_{\hat{V}}:P_{\hat{U}\hat{V}\hat{W}}\in \hat{L}\left(\kappa_{\alpha },\omega \right)}D(P_{\hat{V}}\left|\right|P_{V})+\rho \left(\kappa_{\alpha },\omega \right)+E_{m}\left(P_{SX},\theta \right)-\theta \\ & =\rho \left(\kappa_{\alpha },\omega \right)+E_{m}\left(P_{SX},\theta \right)-\theta :=E_{3}^{i}\left(\kappa_{\alpha },\omega ,P_{SX},\theta \right), & \end{array}$$

where the final equality is since $P_{UV}P_{W|U}\in \hat{L}\left(\kappa_{\alpha },\omega \right)$ for $P_{W|U}:=\omega \left(P_{U}\right)$. The claim in (8) now follows from Theorem 1.

Next, we prove (10). Note that $\hat{L}\left(0,\omega \right)=\left\{P_{UVW}=P_{UV}P_{W|U}:P_{W|U}=\omega \left(P_{U}\right)\right\}$ and $L^{★}\left(0\right)=\left\{\left(\omega ,P_{SX},\theta \right)\in F\times P\left(S\times X\right)\times \Theta \left(P_{SX}\right):\;I_{P}\left(U;W\right)<I_{P}\left(X;Y|S\right),P_{W|U}=\omega \left(P_{U}\right),\;P_{SXY}:=P_{SX}P_{Y|X}\right\}$ since $E_{\mathrm{sp}}\left(P_{SX},\theta \right)\geq 0$ and $E_{\mathrm{ex}}\left(I_{P}\left(U;W\right),P_{SX}\right)\geq 0$. Hence, we have

$$\begin{array}{cc} E_{1}^{i}\left(0,\omega \right) & \geq \min_{P_{\hat{U}\hat{V}\hat{W}}\in \hat{L}\left(0,\omega \right)}I_{P}\left(\hat{V};\hat{W}\right)=I_{P}\left(V;W\right). \end{array}$$

Additionally, $\rho \left(0,\omega \right)=I_{P}\left(V;W\right)$, $E_{2}^{i}\left(0,\omega ,P_{SX}\right)\geq \rho \left(0,\omega \right)$ and $E_{3}^{i}\left(0,\omega ,P_{SX},\theta \right)\geq \rho \left(0,\omega \right)$. By choosing $P_{XS}=P_{X}^{★}P_{S}$ where $P_{X}^{★}$ is the capacity achieving input distribution, we have $I_{P}\left(X;Y|S\right)=C$. Then, it follows from (8) and the continuity of $E_{1}^{i}\left(\kappa_{\alpha },\omega \right)$, $E_{2}^{i}\left(\kappa_{\alpha },\omega ,P_{SX}\right)$ and $E_{3}^{i}\left(\kappa_{\alpha },\omega ,P_{SX},\theta \right)$ in $\kappa_{\alpha }$ that $\lim_{\kappa_{\alpha }\to 0}\kappa \left(\kappa_{\alpha }\right)\geq \kappa_{i}^{★}\left(0\right)$. On the other hand, $\lim_{\kappa_{\alpha }\to 0}\kappa \left(\kappa_{\alpha }\right)\leq \kappa_{i}^{★}\left(0\right)$ follows from the converse proof in Proposition 7 in [10]. The proof of the cardinality bound |W|≤|U|+1 follows from a standard application of the Eggleston–Fenchel–Carathéodory theorem (Theorem18 in [48]), thus completing the proof.

### 4.3. Proof of Corollary 2

Specializing Theorem 1 to TAD, note that $\rho (\kappa_{\alpha },\omega )=0$ since $P_{\hat{U}\hat{V}\hat{W}}=Q_{U}Q_{V}P_{\hat{W}|\hat{U}}\in \hat{L}\left(\kappa_{\alpha },\omega \right)$ and $I_{P}\left(\hat{V};\hat{W}\right)=0$. Additionally, for $R\geq \zeta (\kappa_{\alpha },\omega )$, $E_{b}\left(\kappa_{\alpha },\omega ,R\right)=\infty$. Hence,

$$\begin{array}{cc} \; & L\left(\kappa_{\alpha }\right)-=-\left\{\left(\omega ,R,P_{SX},\theta \right):\begin{array}{cc} \; & \zeta \left(\kappa_{\alpha },\omega \right)\leq R<I_{P}\left(X;Y|S\right),P_{SXY}=P_{SX}P_{Y|X},\\ \; & \min\left\{E_{\mathrm{sp}}\left(P_{SX},\theta \right),E_{\mathrm{ex}}\left(R,P_{SX}\right)\right\}\geq \kappa_{\alpha } \end{array}\right\},\\ \; & \hat{L}\left(\kappa_{\alpha },\omega \right):=\left\{P_{\hat{U}\hat{V}\hat{W}}:\;D\left(P_{\hat{U}\hat{V}\hat{W}}\left|\right|P_{UV\hat{W}}\right)\leq \kappa_{\alpha },P_{\hat{W}|\hat{U}}=\omega \left(P_{\hat{U}}\right),P_{UV\hat{W}}=Q_{U}Q_{V}P_{\hat{W}|\hat{U}}\right\}. \end{array}$$

Then, we have

(37) $$\begin{array}{ccc} E_{1}\left(\kappa_{\alpha },\omega \right):=E_{1}^{d}\left(\kappa_{\alpha },\omega \right) & :=\min_{\begin{array}{c} \left(P_{\tilde{U}\tilde{V}\tilde{W}},Q_{\tilde{U}\tilde{V}\tilde{W}}\right)\in T_{1}\left(\kappa_{\alpha },\omega \right) \end{array}}D(P_{\tilde{U}\tilde{V}\tilde{W}}\left|\right|Q_{\tilde{U}\tilde{V}\tilde{W}})\\ & \overset{\left(a\right)}{\geq }\min_{\begin{array}{c} \left(P_{\tilde{U}\tilde{V}\tilde{W}},Q_{\tilde{U}\tilde{V}\tilde{W}}\right)\in T_{1}\left(\kappa_{\alpha },\omega \right) \end{array}}D(P_{\tilde{V}\tilde{W}}\left|\right|Q_{\tilde{V}\tilde{W}})\\ & \overset{\left(b\right)}{=}\min_{\begin{array}{c} \left(P_{\hat{V}\hat{W}},Q_{V\hat{W}}\right):P_{\hat{U}\hat{V}\hat{W}}\in \hat{L}\left(\kappa_{\alpha },\omega \right),\\ Q_{UV\hat{W}}=Q_{UV}P_{\hat{W}|\hat{U}} \end{array}}D(P_{\hat{V}\hat{W}}\left|\right|Q_{V\hat{W}}), & \end{array}$$

where (a) follows due to the data-processing inequality for KL divergence Theorem 2.15 in [43]; (b) is since $\left(P_{\tilde{U}\tilde{V}\tilde{W}},Q_{\tilde{U}\tilde{V}\tilde{W}}\right)\in T_{1}\left(\kappa_{\alpha },\omega \right)$ implies that $P_{\tilde{V}\tilde{W}}=P_{\hat{V}\hat{W}}$ and $Q_{\tilde{U}\tilde{V}\tilde{W}}=Q_{UV}P_{\hat{W}|\hat{U}}$ for some $P_{\hat{U}\hat{V}\hat{W}}\in \hat{L}\left(\kappa_{\alpha },\omega \right)$. Next, note that since $R\geq \zeta (\kappa_{\alpha },\omega )$, $E_{2}\left(\kappa_{\alpha },\omega ,R\right)=\infty$. Additionally,

(38a) $$\begin{array}{cc} E_{3}\left(\kappa_{\alpha },\omega ,R,P_{SX}\right) & =\min_{\begin{array}{c} \left(P_{\tilde{U}\tilde{V}\tilde{W}},Q_{\tilde{U}\tilde{V}\tilde{W}}\right)\in T_{3}\left(\kappa_{\alpha },\omega \right) \end{array}}D(P_{\tilde{U}\tilde{V}\tilde{W}}\left|\right|Q_{\tilde{U}\tilde{V}\tilde{W}})+E_{\mathrm{ex}}\left(R,P_{SX}\right) \end{array}$$

(38b) $$\begin{array}{cc} & \overset{\left(a\right)}{=}E_{\mathrm{ex}}\left(R,P_{SX}\right), \end{array}$$

$$\begin{array}{cc} E_{4}\left(\kappa_{\alpha },\omega ,P_{SX},\theta \right) & =\min_{P_{\hat{V}}:P_{\hat{U}\hat{V}\hat{W}}\in \hat{L}\left(\kappa_{\alpha },\omega \right)}D(P_{\hat{V}}\left|\right|Q_{V})+E_{m}\left(P_{SX},\theta \right)-\theta \end{array}$$

(38c) $$\begin{array}{ccc} & \overset{\left(b\right)}{=}E_{m}\left(P_{SX},\theta \right)-\theta =:E_{3}^{d}\left(P_{SX},\theta \right), & \end{array}$$

where

(a) is obtained by taking $P_{\hat{U}\hat{V}\hat{W}}=Q_{U}Q_{V}P_{W|U}\in \hat{L}\left(\kappa_{\alpha },\omega \right)$ and $P_{W|U}=\omega \left(Q_{U}\right)$ in the definition of $T_{3}\left(\kappa_{\alpha },\omega \right)$. This implies that $\left(P_{\tilde{U}\tilde{V}\tilde{W}},Q_{\tilde{U}\tilde{V}\tilde{W}}\right)=\left(Q_{UV}P_{W|U},Q_{UV}P_{W|U}\right)\in T_{3}\left(\kappa_{\alpha },\omega \right)$, and hence that the first term in the right hand side (RHS) of (38a) is zero;
(b) is due to $Q_{U}Q_{V}P_{W|U}\in \hat{L}\left(\kappa_{\alpha },\omega \right)$ for $P_{W|U}=\omega \left(Q_{U}\right)$.

Since $E_{\mathrm{ex}}\left(R,P_{SX}\right)$ is a non-increasing function of *R* and $R\geq \zeta (\kappa_{\alpha },\omega )$, selecting $R=\zeta (\kappa_{\alpha },\omega )$ maximizes $E_{3}\left(\kappa_{\alpha },\omega ,R,P_{SX}\right)$. Then, (11) follows from (37), (38b) and (38c).

Next, we prove (12). Note that $\zeta \left(0,\omega \right)=I_{Q}\left(U;W\right)$, where $Q_{UW}=Q_{U}P_{W|U}$, $P_{W|U}=\omega \left(Q_{U}\right)$, and since $E_{\mathrm{sp}}\left(P_{SX},\theta \right)\geq 0$ and $E_{\mathrm{ex}}\left(I_{Q}\left(U;W\right),P_{SX}\right)\geq 0$,

$$\begin{array}{cc} L^{★}\left(0\right) & =\left\{\begin{array}{cc} & \left(\omega ,P_{SX},\theta \right)\in F\times P\left(S\times X\right)\times \Theta \left(P_{SX}\right):\;I_{Q}\left(U;W\right)<I_{P}\left(X;Y|S\right),\\ & Q_{UVW}=Q_{UV}P_{W|U},\;P_{W|U}=\omega \left(Q_{U}\right),P_{SXY}:=P_{SX}P_{Y|X} \end{array}\right\}. \end{array}$$

Additionally, $\hat{L}\left(0,\omega \right)=\left\{Q_{U}Q_{V}P_{W|U}:P_{W|U}=\omega \left(Q_{U}\right)\right\}$. By choosing $\theta =-\theta_{l}\left(P_{SX}\right)$ (defined above (6a)) that maximizes $E_{3}^{d}\left(P_{SX},\theta \right)$, we have

(39a) $$\begin{array}{cc} & E_{1}^{d}\left(0,\omega \right)\geq \min_{\begin{array}{c} \left(P_{\hat{V}\hat{W}},Q_{V\hat{W}}\right):\;P_{\hat{U}\hat{V}\hat{W}}\in \hat{L}\left(0,\omega \right),\\ Q_{UV\hat{W}}=Q_{UV}P_{\hat{W}|\hat{U}} \end{array}}D(P_{\hat{V}\hat{W}}\left|\right|Q_{V\hat{W}})\\ & \;\;\;\;\;=\min_{\begin{array}{c} \left(P_{W|U},P_{SX}\right):\;I_{Q}\left(U;W\right)\leq I_{P}\left(X;Y|S\right),\\ Q_{UVW}=Q_{UV}P_{W|U},P_{SXY}=P_{SX}P_{Y|X} \end{array}}D(Q_{V}Q_{W}\left|\right|Q_{VW}), \end{array}$$

(39b) $$\begin{array}{cc} & E_{2}^{d}\left(0,\omega ,P_{SX}\right)=E_{\mathrm{ex}}\left(I_{Q}\left(U;W\right),P_{SX}\right), \end{array}$$

(39c) $$\begin{array}{ccc} & E_{3}^{d}\left(P_{SX},-\theta_{l}\left(P_{SX}\right)\right)=E_{m}\left(P_{SX},-\theta_{l}\left(P_{SX}\right)\right)+\theta_{l}\left(P_{SX}\right)=\theta_{l}\left(P_{SX}\right), & \end{array}$$

where (39c) is due to $E_{m}\left(P_{SX},-\theta_{l}\left(P_{SX}\right)\right)=0$. The latter in turn follows similar to (A10) and (A11) from the definition of $E_{m}\left(\cdot ,\cdot \right)$. From (11), (39a,39b,39c), and the continuity of $E_{1}^{d}\left(\kappa_{\alpha },\omega \right)$, $E_{2}^{d}\left(\kappa_{\alpha },\omega ,P_{SX}\right)$ in $\kappa_{\alpha }$, (12) follows. The proof of the cardinality bound |W|≤|U|+1 in the RHS of (39a) follows via a standard application of the Eggleston–Fenchel–Carathéodory Theorem (Theorem 18 in [48]). To see this, note that it is sufficient to preserve $\left\{Q_{U}\left(u\right),u\in U\right\}$, $D(Q_{V}Q_{W}||Q_{VW})$ and $H_{Q}\left(U|W\right)$, all of which can be written as a linear combination of functionals of $Q_{U|W}\left(\cdot |w\right)$ with weights $Q_{W}\left(w\right)$. Thus, it requires |U|−1 points to preserve $\left\{Q_{U}\left(u\right),u\in U\right\}$ and one each for $D(Q_{V}Q_{W}||Q_{VW})$ and $H_{Q}\left(U|W\right)$. This completes the proof.

### 4.4. Proof of Theorem 2

We will show that the error-exponent pairs $\left(\kappa_{\alpha },\kappa_{h}^{★}\left(\kappa_{\alpha }\right)\right)$ and $\left(\kappa_{\alpha },\kappa_{u}^{★}\left(\kappa_{\alpha }\right)\right)$ are achieved by a hybrid coding scheme and uncoded transmission scheme, respectively. First, we describe the hybrid coding scheme.

Let n∈N, |W|<∞, $\kappa_{\alpha }>0$, and $(P_{S},\omega^{\prime }\left(\cdot ,P_{S}\right),P_{X|USW},$$P_{X^{\prime }|US})\in L_{h}\left(\kappa_{\alpha }\right)$. Further, let η>0 be a small number, and choose a sequence $s\in T_{n}\left(P_{\hat{S}}\right)$, where $P_{\hat{S}}$ satisfies $D\left(P_{\hat{S}}\left|\right|P_{S}\right)\leq \eta$. Set $R^{\prime }:=\zeta^{\prime }\left(\kappa_{\alpha },\omega^{\prime },P_{\hat{S}}\right)$.

**Encoding:** The encoder performs type-based quantization followed by hybrid coding [40]. The details are as follows:

**Quantization codebook:** Let $D_{n}\left(P_{U},\eta \right)$ be as defined in (15). Consider some ordering on the types in $D_{n}\left(P_{U},\eta \right)$ and denote the elements as $P_{{\hat{U}}_{i}}$, $i\in \left[|D_{n}\left(P_{U},\eta \right)|\right]$. For each joint type $P_{\hat{S}{\hat{U}}_{i}}$ such that $P_{{\hat{U}}_{i}}\in D_{n}\left(P_{U},\eta \right)$ and $\hat{S}⫫{\hat{U}}_{i}$, choose a joint type variable $P_{\hat{S}{\hat{U}}_{i}{\hat{W}}_{i}}$, $P_{{\hat{W}}_{i}}\in T\left(W^{n}\right)$, such that $D\left(P_{{\hat{W}}_{i}|{\hat{U}}_{i}\hat{S}}\left|\right|P_{W_{i}|U\hat{S}}|P_{{\hat{U}}_{i}\hat{S}}\right)\leq \eta /3$ and $I\left(\hat{S},{\hat{U}}_{i};{\hat{W}}_{i}\right)\leq R^{\prime }+\left(\eta /3\right)$, where $P_{W_{i}|U,S}=\omega^{\prime }\left(P_{{\hat{U}}_{i}},P_{\hat{S}}\right)$. Define $D_{n}\left(P_{SUW},\eta \right):=\left\{P_{\hat{S}{\hat{U}}_{i}{\hat{W}}_{i}}:i\in \left[|D_{n}\left(P_{U},\eta \right)|\right]\right\}$, $R_{i}^{\prime }:=I_{P}\left(\hat{S},{\hat{U}}_{i};{\hat{W}}_{i}\right)+\left(\eta /3\right)$ for $i\in \left[|D_{n}\left(P_{U},\eta \right)|\right]$ and $M_{i}^{\prime }:=\left[1+\sum_{m=1}^{i-1}e^{nR_{m}^{\prime }}:\sum_{m=1}^{i}e^{nR_{m}^{\prime }}\right],\;i\in \left[|D_{n}\left(P_{U},\eta \right)|\right]$. Let $B_{W,n}=\left\{W\left(j\right)\in W^{n},1\leq j\leq \sum_{i=1}^{|D_{n}\left(P_{U},\eta \right)|}e^{nR_{i}^{\prime }}\right\}$ denote a random quantization codebook such that for $i\in \left[|D_{n}\left(P_{U},\eta \right)|\right]$, each codeword W(j), $j\in M_{i}^{\prime }$, is independently selected from $T_{n}\left(P_{{\hat{W}}_{i}}\right)$ according to uniform distribution, i.e., $W\left(j\right)∼\;\mathrm{Unif}\left[T_{n}\left(P_{{\hat{W}}_{i}}\right)\right]$. Let $B_{W,n}$ denote a realization of $B_{W,n}$.

**Type-based hybrid coding:** For $u\in T_{n}\left(P_{{\hat{U}}_{i}}\right)$ such that $P_{{\hat{U}}_{i}}\in D_{n}\left(P_{U},\eta \right)$ for some $i\in \left[|D_{n}\left(P_{U},\eta \right)|\right]$, let

$$\bar{M}\left(u,B_{W,n}\right):=\left\{j\in M_{i}^{\prime }:\;w\left(j\right)\in B_{W,n},\;\left(s,u,w\left(j\right)\right)\in T_{n}\left(P_{\hat{S}{\hat{U}}_{i}{\hat{W}}_{i}}\right),\;P_{\hat{S}{\hat{U}}_{i}{\hat{W}}_{i}}\in D_{n}\left(P_{SUW},\eta \right)\right\}.$$

If $|\bar{M}\left(u,B_{W,n}\right)|\geq 1$, let $M^{\prime }\left(u,B_{W,n}\right)$ denote an index selected uniformly at random from the set $\bar{M}\left(u,B_{W,n}\right)$; otherwise, set $M^{\prime }\left(u,B_{W,n}\right)=0$. Given $B_{W,n}$ and $u\in U^{n}$, the quantizer outputs $M^{\prime }=M^{\prime }\left(u,B_{W,n}\right)$, where the support of $M^{\prime }$ is $M^{\prime }:=\left\{0\right\}{⋃}_{i=1}^{|D_{n}\left(P_{U},\eta \right)|}M_{i}^{\prime }$. Note that for sufficiently large *n*, it follows similarly to (18) that $|M^{\prime }|\leq e^{n(R^{\prime }+\eta )}$. For a given $B_{W,n}$ and $u\in U^{n}$, the encoder transmits $X∼P_{X|USW}^{⊗n}\left(\cdot |u,s,w\left(m^{\prime }\right)\right)$ if $M^{\prime }=m^{\prime }\neq 0$, and $X^{\prime }∼P_{X^{\prime }|US}^{⊗n}\left(\cdot |u,s\right)$ if $M^{\prime }=0$.

**Acceptance region:** For a given codebook $B_{W,n}$ and $m^{\prime }\in M^{\prime }\setminus \left\{0\right\}$, let $O_{m^{\prime }}$ denote the set of u such that $M^{\prime }\left(u,B_{W,n}\right)=m^{\prime }$. For each $m^{\prime }\in M^{\prime }\setminus \left\{0\right\}$ and $u\in O_{m^{\prime }}$, set

$$\begin{array}{c} Z_{m^{\prime }}^{\prime }\left(u\right)=\left\{\left(v,y\right)\in V^{n}\times Y^{n}:\left(s,u,{\bar{w}}_{m^{\prime }},v,y\right)\in J_{n}\left(\kappa_{\alpha }+\eta ,P_{\hat{S}UW_{m^{\prime }}VY}\right)\right\}, \end{array}$$

where recall that $J_{n}\left(r,P_{X}\right):=\left\{x\in X^{n}:D\left(P_{x}\left|\right|P_{X}\right)\leq r\right\}$, and

(40a) $$P_{\hat{S}UW_{m^{\prime }}VXY}=P_{\hat{S}}P_{UV}P_{W_{m^{\prime }}|U\hat{S}}P_{X|U\hat{S}W_{m^{\prime }}}P_{Y|X},\;$$

(40b) $$P_{W_{m^{\prime }}|U\hat{S}}=\omega^{\prime }\left(P_{u},P_{\hat{S}}\right)\;\mathrm{and}\;P_{X|U\hat{S}W_{m^{\prime }}}=P_{X|USW}.$$

For $m^{\prime }\in M^{\prime }\setminus \left\{0\right\}$, define $Z_{m^{\prime }}^{\prime }:=\left\{\left(v,y\right):\left(v,y\right)\in Z_{m^{\prime }}^{\prime }\left(u\right)\mathrm{for}\;\mathrm{some}u\in O_{m^{\prime }}\right\}.$ The acceptance region for $H_{0}$ is given by $A_{n}:=\cup_{m^{\prime }\in M^{\prime }\setminus 0}\;s\times m^{\prime }\times Z_{m^{\prime }}^{\prime }$ or equivalently as $A_{n}^{e}:=\cup_{m^{\prime }\in M^{\prime }\setminus 0}\;s\times O_{m^{\prime }}\times Z_{m^{\prime }}^{\prime }.$

**Decoding:** Given codebook $B_{W,n}$, Y=y, and V=v, if $\left(v,y\right)\in {⋃}_{m^{\prime }\in M^{\prime }\setminus \left\{0\right\}}Z_{m^{\prime }}^{\prime }$, then ${\hat{M}}^{\prime }={\hat{m}}^{\prime }$, where ${\hat{m}}^{\prime }:={arg min}_{j\in M^{\prime }\setminus 0}H_{e}\left(w\left(j\right)|v,y,s\right)$. Otherwise, ${\hat{M}}^{\prime }=0$. Denote the decoder induced by the above operations by $g_{B_{W,n}}:S^{n}\times V^{n}\times Y^{n}\to M^{\prime }$.

**Testing:** If ${\hat{M}}^{\prime }=0$, $\hat{H}=1$ is declared. Otherwise, $\hat{H}=0$ or $\hat{H}=1$ is declared depending on whether $\left(s,{\hat{m}}^{\prime },v,y\right)\in A_{n}$ or $\left(s,{\hat{m}}^{\prime },v,y\right)\notin A_{n}$, respectively. Denote the decision function induced by $g_{B_{W,n}}$ and $A_{n}$ by $g_{n}:S^{n}\times V^{n}\times Y^{n}\to \hat{H}$.

**Induced probability distribution:** The PMFs induced by a code $c_{n}=\left(f_{n},g_{n}\right)$ with respect to codebook $B_{W,n}$ under $H_{0}$ and $H_{1}$ are

$$\begin{array}{ccc} & P_{\mathrm{UV}M^{\prime }\mathrm{XY}{\hat{M}}^{\prime }\hat{H}}^{\left(B_{W,n},c_{n}\right)}\left(u,v,m^{\prime },x,y,{\hat{m}}^{\prime },\hat{h}\right)\\ & :=\left\{\begin{array}{cc} P_{UV}^{⊗n}\left(u,v\right)\;{𝟙}_{\left\{M^{\prime }\left(u,B_{W,n}\right)=m^{\prime }\right\}}\;P_{X|USW}^{⊗n}\left(x|s,u,w\left(m^{\prime }\right)\right)\;P_{Y|X}^{⊗n}\left(y|x\right)\;\\ \;\;\;\;{𝟙}_{\left\{g_{B_{W,n}}\left(v,y,s\right)={\hat{m}}^{\prime }\right\}}\;{𝟙}_{\left\{\hat{h}={𝟙}_{\left\{\left(s,{\hat{m}}^{\prime },v,y\right)\in A_{n}^{c}\right\}}\right\}}, & \mathrm{if}\;m^{\prime }\neq 0,\\ P_{UV}^{⊗n}\left(u,v\right)\;{𝟙}_{\left\{M^{\prime }\left(u,B_{W,n}\right)=m^{\prime }\right\}}\;P_{X^{\prime }|US}^{⊗n}\left(x|s,u\right)\;P_{Y|X}^{⊗n}\left(y|x\right)\;\\ \;\;\;\;{𝟙}_{\left\{g_{B_{W,n}}\left(v,y,s\right)={\hat{m}}^{\prime }\right\}}\;{𝟙}_{\left\{\hat{h}={𝟙}_{\left\{\left(s,{\hat{m}}^{\prime },v,y\right)\in A_{n}^{c}\right\}}\right\}}, & \mathrm{otherwise}, \end{array}\right. & \end{array}$$

and

$$\begin{array}{ccc} & Q_{\mathrm{UV}M^{\prime }\mathrm{XY}{\hat{M}}^{\prime }\hat{H}}^{\left(B_{W,n},c_{n}\right)}\left(u,v,m^{\prime },x,y,{\hat{m}}^{\prime },\hat{h}\right)\\ & :=\left\{\begin{array}{cc} Q_{UV}^{⊗n}\left(u,v\right)\;{𝟙}_{\left\{M^{\prime }\left(u,B_{W,n}\right)=m^{\prime }\right\}}\;P_{X|USW}^{⊗n}\left(x|s,u,w\left(m^{\prime }\right)\right)\;P_{Y|X}^{⊗n}\left(y|x\right)\;\\ \;\;\;\;{𝟙}_{\left\{g_{B_{W,n}}\left(v,y,s\right)={\hat{m}}^{\prime }\right\}}\;{𝟙}_{\left\{\hat{h}={𝟙}_{\left\{\left(s,{\hat{m}}^{\prime },v,y\right)\in A_{n}^{c}\right\}}\right\}}, & \mathrm{if}\;m^{\prime }\neq 0,\\ Q_{UV}^{⊗n}\left(u,v\right)\;{𝟙}_{\left\{M^{\prime }\left(u,B_{W,n}\right)=m^{\prime }\right\}}\;P_{X^{\prime }|US}^{⊗n}\left(x|s,u\right)\;P_{Y|X}^{⊗n}\left(y|x\right)\;\\ \;\;\;\;{𝟙}_{\left\{g_{B_{W,n}}\left(v,y,s\right)={\hat{m}}^{\prime }\right\}}\;{𝟙}_{\left\{\hat{h}={𝟙}_{\left\{\left(s,{\hat{m}}^{\prime },v,y\right)\in A_{n}^{c}\right\}}\right\}}, & \mathrm{otherwise}, \end{array}\right. & \end{array}$$

respectively. For brevity, we will denote $B_{W,n}$ by $B_{n}$, $B_{W,n}$ by $B_{n}$, and the above probability distributions by $P^{\left(B_{n}\right)}$ and $Q^{\left(B_{n}\right)}$. Let $B_{n}$ and $\mu_{n}$ stand for the support and probability measure of $B_{n}$, respectively, and set ${\bar{P}}_{P^{\left(B_{n}\right)}}:=E_{\mu_{n}}\left[P_{P^{\left(B_{n}\right)}}\right]$, ${\bar{P}}_{Q^{\left(B_{n}\right)}}:=E_{\mu_{n}}\left[P_{Q^{\left(B_{n}\right)}}\right]$

**Analysis of the type I and type II error probabilities:** We analyze the expected type I and type II error probabilities, where the expectation is with respect to the randomness of $B_{n}$, followed by the expurgation technique to extract a sequence of deterministic codebooks ${\left\{B_{n}\right\}}_{n\in N}$ and a code ${\left\{c_{n}=\left(f_{n},g_{n}\right)\right\}}_{n\in N}$ that achieves the lower bound in Theorem 2.

**Type I error probability:** Denoting by $A_{n}$ the random acceptance region for $H_{0}$, note that a type I error can occur only under the following events:

(i) $E_{\mathrm{EE}}^{\prime }:=\underset{P_{\hat{U}}\in D_{n}\left(P_{U},\eta \right)}{⋃}\underset{u\in T_{n}\left(P_{\hat{U}}\right)}{⋃}E_{\mathrm{EE}}^{\prime }\left(u\right)$, where
  $$\begin{array}{ccc} E_{\mathrm{EE}}^{\prime }\left(u\right):=\left\{\begin{array}{cc} & ∄\;j\in M^{\prime }\setminus \left\{0\right\}s.t.\left(s,u,W\left(j\right)\right)\in T_{n}\left(P_{\hat{S}{\hat{U}}_{i}{\hat{W}}_{i}}\right),\;P_{\hat{S}{\hat{U}}_{i}}=P_{su},\\ & \;P_{\hat{S}{\hat{U}}_{i}{\hat{W}}_{i}}\in D_{n}\left(P_{SUW},\eta \right) \end{array}\right\}, & & \end{array}$$
(ii) $E_{\mathrm{NE}}^{\prime }:=\{{\hat{M}}^{\prime }=M^{\prime }$ and $\left(s,{\hat{M}}^{\prime },V,Y\right)\notin A_{n}\}$,
(iii) $E_{\mathrm{ODE}}^{\prime }:=\{M^{\prime }\neq 0$, ${\hat{M}}^{\prime }\neq M^{\prime }$ and $\left(s,{\hat{M}}^{\prime },V,Y\right)\notin A_{n}\}$,
(iv) $E_{\mathrm{SDE}}^{\prime }:=\{M^{\prime }=0$, ${\hat{M}}^{\prime }\neq M^{\prime }$ and $\left(s,{\hat{M}}^{\prime },V,Y\right)\notin A_{n}\}$.

By definition of $R_{i}^{\prime }$, we have, similar to (24), the following:

(41) $$\begin{array}{c} {\bar{P}}_{P^{B_{n}}}\left(E_{\mathrm{EE}}^{\prime }\right)\leq e^{-e^{n\Omega (\eta )}}. \end{array}$$

Next, the event $E_{\mathrm{NE}}^{\prime }$ can be upper bounded as

(42) $$\begin{array}{cc} {\bar{P}}_{P^{B_{n}}}\left(E_{\mathrm{NE}}^{\prime }|E_{\mathrm{EE}}^{\prime c}\right) & \leq {\bar{P}}_{P^{B_{n}}}\left(\left(s,{\hat{M}}^{\prime },V,Y\right)\notin A_{n}|{\hat{M}}^{\prime }=M^{\prime },E_{EE}^{\prime c}\right)\\ & =1-{\bar{P}}_{P^{B_{n}}}\left(\left(s,U,V,Y\right)\in A_{n}^{e}|E_{EE}^{\prime c}\right). \end{array}$$

For $u\in O_{m^{\prime }}$, note that, similar to Equation 4.17 in [14], we have

$$\begin{array}{c} {\bar{P}}_{P^{B_{n}}}\left(\left(V,Y\right)\in Z_{m^{\prime }}^{\prime }\left(u\right)|U=u,W\left(m^{\prime }\right)={\bar{w}}_{m^{\prime }},E_{EE}^{\prime c}\right)\geq 1-e^{-n\left(\kappa_{\alpha }+\frac{\eta }{3}-D(P_{u}\left|\right|P_{U})\right)}. \end{array}$$

From this and (15), we obtain, similar to Equation (4.22) in [14] that

(43) $$\begin{array}{c} {\bar{P}}_{P^{B_{n}}}\left(\left(s,U,V,Y\right)\in A_{e}^{n}|E_{EE}^{\prime c}\right)\geq 1-e^{-n\kappa_{\alpha }}. \end{array}$$

Substituting (43) in (42) yields

(44) $$\begin{array}{cc} \; & {\bar{P}}_{P^{B_{n}}}\left(E_{\mathrm{NE}}^{\prime }|E_{\mathrm{EE}}^{\prime c}\right)\leq e^{-n\kappa_{\alpha }}. \end{array}$$

Next, we bound the probability of the event $E_{\mathrm{ODE}}^{\prime }$ as follows:

$$\begin{array}{cc} & {\bar{P}}_{P^{B_{n}}}\left(E_{\mathrm{ODE}}^{\prime }\right)\\ & ={\bar{P}}_{P^{B_{n}}}\left(M^{\prime }\neq 0,{\hat{M}}^{\prime }\neq M^{\prime },\left(s,M^{\prime },V,Y\right)\in A_{n},\left(s,{\hat{M}}^{\prime },V,Y\right)\notin A_{n}\right)\\ & \;\;\;\;+{\bar{P}}_{P^{B_{n}}}\left(M^{\prime }\neq 0,{\hat{M}}^{\prime }\neq M^{\prime },\left(s,M^{\prime },V,Y\right)\notin A_{n},\left(s,{\hat{M}}^{\prime },V,Y\right)\notin A_{n}\right)\\ & \leq {\bar{P}}_{P^{B_{n}}}\left(M^{\prime }\neq 0,{\hat{M}}^{\prime }\neq M^{\prime },\left(s,M^{\prime },V,Y\right)\in A_{n},\left(s,{\hat{M}}^{\prime },V,Y\right)\notin A_{n}\right)\\ & \;\;\;\;+{\bar{P}}_{P^{B_{n}}}\left(M^{\prime }\neq 0,{\hat{M}}^{\prime }\neq M^{\prime },\left(s,M^{\prime },V,Y\right)\notin A_{n}\right) \end{array}$$

(45) $$\begin{array}{cc} & \overset{\left(a\right)}{\leq }{\bar{P}}_{P^{B_{n}}}\left(M^{\prime }\neq 0,{\hat{M}}^{\prime }\neq M^{\prime },\left(s,M^{\prime },V,Y\right)\in A_{n},\left(s,{\hat{M}}^{\prime },V,Y\right)\notin A_{n}\right)+e^{-e^{n\Omega (\eta )}}+e^{-n\kappa_{\alpha }} \end{array}$$

$$\begin{array}{cc} & \leq {\bar{P}}_{P^{B_{n}}}\left({\hat{M}}^{\prime }\neq M^{\prime }|M^{\prime }\neq 0,\left(s,M^{\prime },V,Y\right)\in A_{n}\right)+e^{-e^{n\Omega (\eta )}}+e^{-n\kappa_{\alpha }}, \end{array}$$

(46) $$\begin{array}{cc} & \overset{\left(b\right)}{=}{\bar{P}}_{P^{B_{n}}}\left({\hat{M}}^{\prime }\neq M^{\prime }|M^{\prime }\neq 0,{\hat{M}}^{\prime }\neq 0,\left(s,M^{\prime },V,Y\right)\in A_{n}\right) \end{array}$$

(47) $$\begin{array}{cc} & \overset{\left(c\right)}{\leq }e^{-n\left(\rho^{\prime }\left(\kappa_{\alpha },\omega^{\prime },P_{S},P_{X|USW}\right)-\zeta^{\prime }\left(\kappa_{\alpha },\omega^{\prime },P_{\hat{S}}\right)-O\left(\eta \right)\right)}, \end{array}$$

where (a) follows similar to (29) using (41) and (43); (b) is since $\left(s,M^{\prime },V,Y\right)\in A_{n}$ implies that ${\hat{M}}^{\prime }\neq 0$; and (c) follows similar to (33). Further,

(48) $$\begin{array}{cccc} {\bar{P}}_{P^{B_{n}}}\left(E_{\mathrm{SDE}}^{\prime }\right) & \leq {\bar{P}}_{P^{B_{n}}}\left(M^{\prime }=0\right)\\ & \leq {\bar{P}}_{P^{B_{n}}}\left(M^{\prime }=0|E_{EE}^{\prime c}\right)+{\bar{P}}_{P^{B_{n}}}\left(E_{EE}^{\prime }\right)\\ & =\sum_{\begin{array}{c} u:P_{u}\notin D_{n}\left(P_{U},\eta \right) \end{array}}P_{U}^{⊗n}\left(u\right)+{\bar{P}}_{P^{B_{n}}}\left(E_{EE}^{\prime }\right)\\ & \leq e^{-n\kappa_{\alpha }}+e^{-e^{n\Omega (\eta )}}, & & \end{array}$$

where the penultimate equality is since given $E_{EE}^{\prime c}$, $M^{\prime }=0$ occurs only for U=u such that $P_{u}\notin D_{n}\left(P_{U},\eta \right)$, and the final inequality follows from (41), the definition of $D_{n}\left(P_{U},\eta \right)$ and Lemma 1.6 in [38]. From (41), (44), (47) and (48), the expected type I error probability satisfies $e^{-n(\kappa_{\alpha }-O\left(\eta \right))}$ for sufficiently large *n* via the union bound.

**Type II error probability:** Next, we analyze the expected type II error probability over $B_{n}$. Let

$$\begin{array}{cc} \; & D_{n}\left(P_{SVWY},\eta \right):=\left\{\begin{array}{cc} P_{\hat{S}\hat{V}\hat{W}\hat{Y}}: & \;\exists \;\left(s,u,v,\bar{w},y\right)\in \underset{m^{\prime }\in M^{\prime }\setminus \left\{0\right\}}{\cup }J_{n}\left(\kappa_{\alpha }+\eta ,P_{\hat{S}UVW_{m^{\prime }}Y}\right),\\ & P_{\hat{S}UVW_{m^{\prime }}Y}\mathrm{satisfies}\left(40\right)\;\mathrm{and}\;P_{suv\bar{w}y}=P_{\hat{S}\hat{U}\hat{V}\hat{W}\hat{Y}} \end{array}\right\},\\ \; & F_{1,n}^{\prime }\left(\eta \right):=\left\{\begin{array}{cc} P_{\hat{S}\tilde{U}\tilde{V}\tilde{W}\tilde{Y}}\in T\left(S^{n}\times U^{n}\times V^{n}\times W^{n}\times Y^{n}\right) & :P_{\hat{S}\tilde{U}\tilde{W}}\in D_{n}\left(P_{SUW},\eta \right),\\ \; & P_{\hat{S}\tilde{V}\tilde{W}\tilde{Y}}\in D_{n}\left(P_{SVWY},\eta \right) \end{array}\right\}. \end{array}$$

A type II error can occur only under the following events:

*(a)*  $E_{a}^{\prime }:=\left\{\begin{array}{cc} & {\hat{M}}^{\prime }=M^{\prime }\neq 0,\left(s,U,V,W\left(M^{\prime }\right),Y\right)\in T_{n}\left(P_{\hat{S}\hat{U}\hat{V}\hat{W}\hat{Y}}\right)\\ & s.t.P_{\hat{U}\hat{W}}\in D_{n}\left(P_{SUW},\eta \right)\;\mathrm{and}\;P_{\hat{S}\hat{V}\hat{W}\hat{Y}}\in D_{n}\left(P_{SVWY},\eta \right) \end{array}\right\}$,
*(b)*  $E_{b}^{\prime }:=\left\{\begin{array}{cc} & M^{\prime }\neq 0,{\hat{M}}^{\prime }\neq M^{\prime },\left(s,U,V,W\left(M^{\prime }\right),Y,W\left({\hat{M}}^{\prime }\right)\right)\in T_{n}\left(P_{\hat{S}\hat{U}\hat{V}\hat{W}\hat{Y}{\hat{W}}_{d}}\right)\\ & s.t.P_{\hat{S}\hat{U}\hat{W}}\in D_{n}\left(P_{SUW},\eta \right),P_{\hat{S}\hat{V}{\hat{W}}_{d}\hat{Y}}\in D_{n}\left(P_{SVWY},\eta \right),\\ & \;\mathrm{and}\;H_{e}\left(W({\hat{M}}^{\prime })|s,V,Y\right)\leq H_{e}\left(W(M^{\prime })|s,V,Y\right) \end{array}\right\}$,
*(c)*  $E_{c}^{\prime }:=\left\{\begin{array}{cc} & M^{\prime }=0,{\hat{M}}^{\prime }\neq M^{\prime },\left(s,V,Y,W\left({\hat{M}}^{\prime }\right)\right)\in T_{n}\left(P_{\hat{S}\hat{V}\hat{Y}{\hat{W}}_{d}}\right)\\ & s.t.P_{\hat{S}\hat{V}{\hat{W}}_{d}\hat{Y}}\in D_{n}\left(P_{SVWY},\eta \right) \end{array}\right\}.$

Considering the event $E_{a}^{\prime }$, we have

(49) $$\begin{array}{ccc} & {\bar{P}}_{Q^{B_{n}}}\left(E_{a}^{\prime }\right)\\ & \leq \sum_{\begin{array}{c} P_{\hat{S}\tilde{U}\tilde{V}\tilde{W}\tilde{Y}}\\ \in F_{1,n}^{\prime }\left(\eta \right) \end{array}}\sum_{\begin{array}{c} (u,v,\bar{w},y):\\ \left(s,u,v,\bar{w},y\right)\\ \in T_{n}\left(P_{\hat{S}\tilde{U}\tilde{V}\tilde{W}\tilde{Y}}\right) \end{array}}\sum_{\begin{array}{c} m^{\prime }\in M^{\prime }\setminus \left\{0\right\} \end{array}}{\bar{P}}_{Q^{B_{n}}}\left(U=u,V=v,M^{\prime }=m^{\prime },W\left(m^{\prime }\right)=\bar{w},Y=y|S=s\right)\\ & \leq \sum_{\begin{array}{c} P_{\hat{S}\tilde{U}\tilde{V}\tilde{W}\tilde{Y}}\in F_{1,n}^{\prime }\left(\eta \right) \end{array}}\sum_{\begin{array}{c} (u,v,\bar{w},y):\\ \left(s,u,v,\bar{w},y\right)\in T_{n}\left(P_{\hat{S}\tilde{U}\tilde{V}\tilde{W}\tilde{Y}}\right) \end{array}}\sum_{m^{\prime }\in M^{\prime }\setminus \left\{0\right\}}{\bar{P}}_{Q^{B_{n}}}\left(U=u,V=v,M^{\prime }=m^{\prime }|S=s\right)\\ & \;\;\;\;\;\;\;{\bar{P}}_{Q^{B_{n}}}\left(W\left(m^{\prime }\right)=\bar{w}|U=u,V=v,M^{\prime }=m^{\prime },S=s\right)\\ & \;\;\;\;\;\;\;{\bar{P}}_{Q^{B_{n}}}\left(Y=y|U=u,V=v,M^{\prime }=m^{\prime },W\left(m^{\prime }\right)=\bar{w},S=s\right)\\ & \overset{\left(a\right)}{\leq }\sum_{\begin{array}{c} P_{\hat{S}\tilde{U}\tilde{V}\tilde{W}\tilde{Y}}\in F_{1,n}^{\prime }\left(\eta \right) \end{array}}\sum_{\begin{array}{c} (u,v,\bar{w},y):\\ \left(s,u,v,\bar{w},y\right)\in T_{n}\left(P_{\hat{S}\tilde{U}\tilde{V}\tilde{W}\tilde{Y}}\right) \end{array}}e^{-n\left(H\left(\tilde{U},\tilde{V}\right)+D\left(P_{\tilde{U}\tilde{V}}\left|\right|Q_{UV}\right)\right)}\;e^{-n\left(H(\tilde{W}|\hat{S},\tilde{U})-\eta \right)}\\ & \;\;\;\;\;\;\;\;e^{-n\left(H\left(\tilde{Y}|\tilde{U},\hat{S},\tilde{W}\right)+D\left(P_{\tilde{Y}|\tilde{U}\hat{S}\tilde{W}}\left|\right|P_{Y|USW}|P_{\tilde{U}\hat{S}\tilde{W}}\right)\right)}\\ & \leq \sum_{\begin{array}{c} P_{\hat{S}\tilde{U}\tilde{V}\tilde{W}\tilde{Y}}\in F_{1,n}^{\prime }\left(\eta \right) \end{array}}e^{nH(\tilde{U},\tilde{V},\tilde{W},\tilde{Y}|\hat{S})}e^{-n\left(H\left(\tilde{U},\tilde{V}\right)+D\left(P_{\tilde{U}\tilde{V}}\left|\right|Q_{UV}\right)\right)}\;e^{-n\left(H(\tilde{W}|\hat{S},\tilde{U})-\eta \right)}\\ & \;\;\;\;\;\;\;e^{-n\left(H\left(\tilde{Y}|\tilde{U},\hat{S},\tilde{W}\right)+D\left(P_{\tilde{Y}|\tilde{U}\hat{S}\tilde{W}}\left|\right|P_{Y|USW}|P_{\tilde{U}\hat{S}\tilde{W}}\right)\right)}\\ & \leq e^{-nE_{1,n}^{\prime }}, & \end{array}$$

where

$$\begin{array}{cc} E_{1,n}^{\prime } & :=\min_{P_{\hat{S}\tilde{U}\tilde{V}\tilde{W}\tilde{Y}}\in F_{1,n}^{\prime }\left(\eta \right)}H\left(\tilde{U},\tilde{V}\right)+D\left(P_{\tilde{U}\tilde{V}}\left|\right|Q_{UV}\right)+H\left(\tilde{W}|\hat{S},\tilde{U}\right)-\eta +H\left(\tilde{Y}|\tilde{U},\hat{S},\tilde{W}\right)\\ \; & \;+D\left(P_{\tilde{Y}|\tilde{U}\hat{S}\tilde{W}}\left|\right|P_{Y|USW}|P_{\tilde{U}\hat{S}\tilde{W}}\right)-H\left(\tilde{U},\tilde{V},\tilde{W},\tilde{Y}|\hat{S}\right)-\frac{1}{n}||U||V||W||Y|\log\left(n+1\right)\\ \; & ≳\min_{\begin{array}{c} \left(P_{\tilde{U}\tilde{V}\tilde{W}\tilde{Y}S},Q_{\tilde{U}\tilde{V}\tilde{W}\tilde{Y}S}\right)\\ \in T_{1}^{\prime }\left(\kappa_{\alpha },\omega^{\prime },P_{S},P_{X|USW}\right) \end{array}}D(P_{\tilde{U}\tilde{V}\tilde{W}\tilde{Y}|S}\left|\right|Q_{UVWY|S}\left|P_{S}\right)-O\left(\eta \right)\\ \; & =E_{1}^{\prime }\left(\kappa_{\alpha },\omega^{\prime }\right)-O\left(\eta \right). \end{array}$$

For the inequality in (a) above, we used $\sum_{}{\bar{P}}_{Q^{B_{n}}}\left(M^{\prime }=m^{\prime }|U=u,V=v,S=s\right)\leq 1$ and

$$\begin{array}{c} {\bar{P}}_{Q^{B_{n}}}\left(W\left(m^{\prime }\right)=\bar{w}|U=u,V=v,S=s,M^{\prime }=m^{\prime }\right)\leq \left\{\begin{array}{cc} e^{-n\left(H(\tilde{W}|\hat{S},\tilde{U})-\eta \right)}, & \mathrm{if}\;\bar{w}\in T_{n}\left(\tilde{W}\right),\\ 0, & \mathrm{otherwise}, \end{array}\right. \end{array}$$

which in turn follows from the fact that given $M^{\prime }=m^{\prime }$ and U=u, $W(m^{\prime })$ is uniformly distributed in the set $T_{n}\left(P_{\tilde{W}|\hat{S}\tilde{U}},s,u\right)$ and that for sufficiently large *n*$|T_{n}\left(P_{\tilde{W}|\hat{S}\tilde{U}},s,u\right)|\geq e^{n\left(H(\tilde{W}|\hat{S},\tilde{U})-\eta \right)}$.

Next, we analyze the probability of the event $E_{b}^{\prime }$. Let

$$\begin{array}{ccc} F_{2,n}^{\prime }\left(\eta \right):=\left\{\begin{array}{cc} P_{\hat{S}\tilde{U}\tilde{V}\tilde{W}\tilde{Y}{\tilde{W}}_{d}}: & P_{\hat{S}\tilde{U}\tilde{W}}\in D_{n}\left(P_{SUW},\eta \right),P_{\hat{S}\tilde{V}{\tilde{W}}_{d}\tilde{Y}}\in D_{n}\left(P_{SVWY},\eta \right)\\ & H\left({\tilde{W}}_{d}|\hat{S},\tilde{V},\tilde{Y}\right)\leq H\left(\tilde{W}|\hat{S},\tilde{V},\tilde{Y}\right) \end{array}\right\}. & \; & \end{array}$$

Then,

(50) $$\begin{array}{ccc} & {\bar{P}}_{Q^{B_{n}}}\left(E_{b}^{\prime }\right)\\ & \leq \sum_{\begin{array}{c} P_{\hat{S}\tilde{U}\tilde{V}\tilde{W}\tilde{Y}{\tilde{W}}_{d}}\\ \in F_{2,n}^{\prime }\left(\eta \right) \end{array}}\sum_{\begin{array}{c} (u,v,\bar{w},y,w^{\prime }):\\ \left(s,u,v,\bar{w},y,w^{\prime }\right)\\ \in T_{n}\left(P_{\hat{S}\tilde{U}\tilde{V}\tilde{W}\tilde{Y}{\tilde{W}}_{d}}\right) \end{array}}\sum_{\begin{array}{c} m^{\prime }\in \\ M^{\prime }\setminus \left\{0\right\} \end{array}}{\bar{P}}_{Q^{B_{n}}}\left(U=u,V=v,M^{\prime }=m^{\prime },W\left(m^{\prime }\right)=\bar{w},Y=y|S=s\right)\\ & \;\;\;\sum_{\begin{array}{c} {\hat{m}}^{\prime }\in M^{\prime }\setminus \left\{0,m^{\prime }\right\} \end{array}}{\bar{P}}_{Q^{B_{n}}}\left(\bar{W}\left({\hat{m}}^{\prime }\right)=w^{\prime }|U=u,M^{\prime }=m^{\prime },W\left(m^{\prime }\right)=\bar{w},S=s\right)\\ & \leq \sum_{\begin{array}{c} P_{\hat{S}\tilde{U}\tilde{V}\tilde{W}\tilde{Y}{\tilde{W}}_{d}}\\ \in F_{2,n}^{\prime }\left(\eta \right) \end{array}}\sum_{\begin{array}{c} (u,v,\bar{w},y):\\ \left(s,u,v,\bar{w},y\right)\in T_{n}\left(P_{\hat{S}\tilde{U}\tilde{V}\tilde{W}\tilde{Y}}\right) \end{array}}e^{-n\left(H\left(\tilde{U},\tilde{V}\right)+D\left(P_{\tilde{U}\tilde{V}}\left|\right|Q_{UV}\right)\right)}\;e^{-n\left(H(\tilde{W}|\hat{S},\tilde{U})-\eta \right)}\;\\ & \;\;e^{-n\left(H\left(\tilde{Y}|\tilde{U},\hat{S},\tilde{W}\right)+D\left(P_{\tilde{Y}|\tilde{U}\hat{S}\tilde{W}}\left|\right|P_{Y|USW}|P_{\tilde{U}\hat{S}\tilde{W}}\right)\right)}\;e^{n\left(\zeta^{\prime }\left(\kappa_{\alpha },\omega^{\prime },P_{\hat{S}}\right)+\eta \right)}\frac{2e^{nH({\tilde{W}}_{d}|\hat{S},\tilde{V},\tilde{Y})}}{e^{n\left(H({\tilde{W}}_{d})-\eta \right)}}\\ & \leq e^{-nE_{2,n}^{\prime }}, & \end{array}$$

where

$$\begin{array}{ccc} E_{2,n}^{\prime } & ≳\min_{\begin{array}{c} \left(P_{\tilde{U}\tilde{V}\tilde{W}\tilde{Y}S},Q_{\tilde{U}\tilde{V}\tilde{W}\tilde{Y}S}\right)\in T_{2}^{\prime }\left(\kappa_{\alpha },\omega^{\prime },P_{S},P_{X|USW}\right) \end{array}}D(P_{\tilde{U}\tilde{V}\tilde{W}\tilde{Y}|S}\left|\right|Q_{UVWY|S}\left|P_{S}\right)+\rho^{\prime }\left(\kappa_{\alpha },\omega^{\prime },P_{S},P_{X|USW}\right)\\ & \;\;\;\;\;\;\;\;\;\;-\zeta^{\prime }\left(\kappa_{\alpha },\omega^{\prime },P_{S}\right)-O\left(\eta \right)\\ & =E_{2}^{\prime }\left(\kappa_{\alpha },\omega^{\prime },P_{S},P_{X|USW}\right)-O\left(\eta \right). & \end{array}$$

Finally, considering the event $E_{c}^{\prime }$, we have

$$\begin{array}{cc} & {\bar{P}}_{Q^{B_{n}}}\left(E_{c}^{\prime }\right)=\sum_{\begin{array}{c} u\in T_{n}\left(P_{\tilde{U}}\right):\\ P_{\tilde{U}}\in D_{n}\left(P_{U},\eta \right) \end{array}}{\bar{P}}_{Q^{B_{n}}}\left(U=u,E_{\mathrm{EE}}^{\prime },E_{c}^{\prime }|S=s\right)+\sum_{\begin{array}{c} u\in T_{n}\left(P_{\tilde{U}}\right):\\ P_{\tilde{U}}\notin D_{n}\left(P_{U},\eta \right) \end{array}}{\bar{P}}_{Q^{B_{n}}}\left(U=u,E_{c}^{\prime }|S=s\right). \end{array}$$

The first term in the RHS decays double exponentially as $e^{-e^{n\Omega (\eta )}}$, while the second term can be handled as follows:

(51) $$\begin{array}{ccc} & \sum_{\begin{array}{c} u\in T_{n}\left(P_{\tilde{U}}\right):P_{\tilde{U}}\notin D_{n}\left(P_{U},\eta \right) \end{array}}{\bar{P}}_{Q^{B_{n}}}\left(U=u,E_{c}^{\prime }|S=s\right)\\ & \leq \sum_{\begin{array}{c} u\in T_{n}\left(P_{\tilde{U}}\right):\\ P_{\tilde{U}}\notin D_{n}\left(P_{U},\eta \right) \end{array}}\sum_{\begin{array}{c} (v,y,w^{\prime }):\\ \left(s,v,y,w^{\prime }\right)\in T_{n}\left(P_{\hat{S}\tilde{V}\tilde{Y}{\tilde{W}}_{d}}\right),\\ P_{\hat{S}\tilde{V}{\tilde{W}}_{d}\tilde{Y}}\in D_{n}\left(P_{SVWY},\eta \right) \end{array}}\;\sum_{\hat{m}\in M\setminus \left\{0\right\}}{\bar{P}}_{Q^{B_{n}}}\left(U=u,V=v,M^{\prime }=0,Y=y|S=s\right)\\ & \;\;\;\;\;\;\;\;\;\;\;\;\sum_{{\hat{m}}^{\prime }\in M^{\prime }\setminus \left\{0\right\}}{\bar{P}}_{Q^{B_{n}}}\left(W\left({\hat{m}}^{\prime }\right)=\bar{w}\right)\\ & \leq \sum_{\begin{array}{c} P_{\tilde{U}\hat{S}\tilde{V}{\tilde{W}}_{d}\tilde{Y}}\in D_{n}{\left(P_{U},\eta \right)}^{c}\\ \times D_{n}\left(P_{SVWY},\eta \right) \end{array}}e^{nH(\tilde{U},\tilde{V},\tilde{Y}|\hat{S})}e^{-n\left(H\left(\tilde{U},\tilde{V},\tilde{Y}|\hat{S}\right)+D\left(P_{\tilde{U}\tilde{V}\tilde{Y}|\hat{S}}\left|\right|Q_{UVY^{\prime }|\hat{S}}|P_{\hat{S}}\right)\right)}\;\frac{e^{nH({\tilde{W}}_{d}|\hat{S},\tilde{V},\tilde{Y})}e^{n(R^{\prime }+\eta )}}{e^{n\left(H({\tilde{W}}_{d})-\eta \right)}}\\ & \leq e^{-nE_{3,n}^{\prime }}, & \end{array}$$

where

$$\begin{array}{cc} E_{3,n}^{\prime }\; & ≳\min_{\begin{array}{c} P_{\hat{V}\hat{Y}S}:P_{\hat{U}\hat{V}\hat{W}\hat{Y}S}\;\in \\ {\hat{L}}_{h}\left(\kappa_{\alpha },\omega^{\prime },P_{S},P_{X|USW}\right) \end{array}}-D\left(P_{\hat{V}\hat{Y}|S}\left|\right|Q_{VY^{\prime }|S}|P_{S}\right)+\rho^{\prime }\left(\kappa_{\alpha },\omega^{\prime },P_{S},P_{X|USW}\right)-\zeta^{\prime }\left(\kappa_{\alpha },\omega^{\prime },P_{S}\right)-O\left(\eta \right)\\ \; & =E_{3}^{\prime }\left(\kappa_{\alpha },\omega^{\prime },P_{S},P_{X|USW},P_{X^{\prime }|US}\right)-O\left(\eta \right). \end{array}$$

Since the exponent of the type II error probability is lower bounded by the minimum of the exponent of the type II error-causing events, it follows from (49), (50) and (51) that for a fixed $\left(P_{S},\omega^{\prime }\left(\cdot ,P_{S}\right),P_{X|USW},P_{X^{\prime }|US}\right)\in L_{h}\left(\kappa_{\alpha }\right)$

(52a) $$\begin{array}{cc} {\bar{P}}_{P^{\left(B_{n}\right)}}\left(\hat{H}=1\right) & \leq e^{-n(\kappa_{\alpha }-O\left(\eta \right))}, \end{array}$$

(52b) $$\begin{array}{cc} {\bar{P}}_{Q^{\left(B_{n}\right)}}\left(\hat{H}=0\right) & \leq e^{-n\left({\bar{\kappa }}_{h}\left(\kappa_{\alpha },\omega^{\prime },P_{S},P_{X|USW},P_{X^{\prime }|US}\right)-O\left(\eta \right)\right)}, \end{array}$$

where ${\bar{\kappa }}_{h}=\min\left\{E_{1}^{\prime }\left(\kappa_{\alpha },\omega^{\prime }\right),E_{2}^{\prime }\left(\kappa_{\alpha },\omega^{\prime },P_{S},P_{X|USW}\right),E_{3}^{\prime }\left(\kappa_{\alpha },\omega^{\prime },P_{S},P_{X|USW},P_{X^{\prime }|US}\right)\right\}$. Performing expurgation as in the proof of Theorem 1 to obtain a deterministic codebook $B_{n}$ satisfying (52a, 52b), maximizing over $\left(P_{S},\omega^{\prime }\left(\cdot ,P_{S}\right),P_{X|USW},P_{X^{\prime }|US}\right)\in L_{h}\left(\kappa_{\alpha }\right)$ and noting that η>0 is arbitrary yields $\kappa \left(\kappa_{\alpha }\right)\geq \kappa_{h}^{★}\left(\kappa_{\alpha }\right)$.

Finally, we show that $\kappa \left(\kappa_{\alpha }\right)\geq \kappa_{u}^{★}\left(\kappa_{\alpha }\right)$, which will complete the proof. Fix $P_{X|US}$ and let $P_{UVXY}:=P_{UV}P_{X|US}P_{Y|X}$ and $Q_{UVXY}:=Q_{UV}P_{X|US}P_{Y|X}$. Consider an uncoded transmission scheme in which the channel input $X∼f_{n}\left(\cdot |u\right)=P_{X|US}^{⊗n}\left(\cdot |u,s\right)$. Let the decision rule $g_{n}$ be specified by the acceptance region $A_{n}=\left\{\left(s,v,y\right):D\left(P_{vy|s}\left|\right|P_{VY|S}|P_{s}\right)\leq \kappa_{\alpha }+\eta \right\}$ for some small η>0. Then, it follows from Lemma 2.6 in [42] that for sufficiently large *n*,

$$\begin{array}{cc} \alpha_{n}\left(f_{n},g_{n}\right) & =P_{VY|S}^{⊗n}\left(A_{n}^{c}|s\right)\leq e^{-n\kappa_{\alpha }},\\ \beta_{n}\left(f_{n},g_{n}\right) & =Q_{VY|S}^{⊗n}\left(A_{n}|s\right)\leq e^{-n(\kappa_{u}^{★}\left(\kappa_{\alpha }\right)-O\left(\eta \right))}. \end{array}$$

The proof is complete by noting that η>0 is arbitrary.

## 5. Conclusions

This work explored the trade-off between the type I and type II error-exponents for distributed hypothesis testing over a noisy channel. We proposed a separate hypothesis testing and channel coding scheme as well as a joint scheme utilizing hybrid coding, and analyzed their performance resulting in two inner bounds on the error-exponents trade-off. The separate scheme recovers some of the existing bounds in the literature as special cases. We also showed via an example of testing against dependence that the joint scheme strictly outperforms the separate scheme at some points of the error-exponents trade-off. An interesting avenue for future research is the exploration of novel outer bounds that could shed light on the scenarios where the separate or joint schemes are tight.

## Data Availability

Not applicable.

## Abbreviations

The following abbreviations are used in this manuscript:

HT	Hypothesis testing
DHT	Distributed hypothesis testing
TAD	Testing against dependence
TAI	Testing against independence
SHTCC	Separate hypothesis testing and channel coding
JHTCC	Joint hypothesis testing and channel coding

## Appendix A. Proof that Theorem 1 Recovers Theorem 2 in [10]

We prove that $\lim_{\kappa_{\alpha }\to 0}\kappa_{s}^{★}\left(\kappa_{\alpha }\right)=\kappa_{s}$, where $\kappa_{s}$ is the lower bound on the type II error-exponent for a fixed type I error probability constraint and unit bandwidth ratio established in Theorem 2 in [10]. Note that $\hat{L}\left(0,\omega \right)=\left\{P_{UVW}=P_{UV}P_{W|U},P_{W|U}=\omega \left(P_{U}\right)\right\}$, $\zeta \left(0,\omega \right)=I_{P}\left(U;W\right)$, and $\rho \left(0,\omega \right)=I_{P}\left(V;W\right)$. The result then follows from Theorem 1 by noting that $\hat{L}\left(\kappa_{\alpha },\omega \right)$, $\zeta (\kappa_{\alpha },\omega )$ and $\rho (\kappa_{\alpha },\omega )$ are continuous in $\kappa_{\alpha }$ and the fact that $E_{\mathrm{sp}}\left(P_{SX},\theta \right),E_{\mathrm{ex}}\left(R,P_{SX}\right)$ and $E_{b}\left(\kappa_{\alpha },\omega ,R\right)$ are all greater than or equal to zero.

## Appendix B. Proof that Theorem 2 Recovers Theorem 5 in [10]

We show that $\lim_{\kappa_{\alpha }\to 0}\kappa_{h}^{★}\left(\kappa_{\alpha }\right)=\kappa_{h}$, where $\kappa_{h}$ is as defined in Theorem 5 in [10]. Note that ${\hat{L}}_{h}\left(0,\omega^{\prime },P_{S},P_{X|USW}\right):=\left\{P_{UV\hat{W}YS}:\;P_{SUVWXY}:=P_{S}P_{UV}P_{W|US}P_{X|USW}P_{Y|X},\;P_{W|US}=\omega^{\prime }\left(P_{U},P_{S}\right)\right\}$, $\zeta^{\prime }\left(0,\omega^{\prime },P_{S}\right):=I_{P}\left(U;W|S\right)$, $\rho \left(0,\omega^{\prime },P_{S},P_{X|USW}\right)=I_{P}\left(Y,V;W|S\right)$,

$$\begin{array}{c} L_{h}\left(0\right):=\left\{\left(P_{S},\omega^{\prime }\left(\cdot ,P_{S}\right),P_{X|USW},P_{X^{\prime }|US}\right):I_{P}\left(U;W|S\right)<I_{P}\left(Y,V;W|S\right)\right\}, \end{array}$$

and $E_{b}^{\prime }\left(0,\omega^{\prime },P_{S},P_{X|USW}\right)=I_{P}\left(Y,V;W|S\right)-I_{P}\left(U;W|S\right)$. The result then follows from Theorem 2 via the continuity of ${\hat{L}}_{h}\left(\kappa_{\alpha },\cdot ,\cdot ,\cdot \right)$, $\zeta^{\prime }\left(\kappa_{\alpha },\cdot ,\cdot \right)$, $\rho (\kappa_{\alpha },\cdot ,\cdot ,\cdot )$, $L_{h}\left(\kappa_{\alpha }\right)$ and $E_{b}^{\prime }\left(\kappa_{\alpha },\cdot ,\cdot ,\cdot \right)$ in $\kappa_{\alpha }$.

## Appendix C. An Auxiliary Result

Here, we prove a result which was used in the proof of Theorem 1, namely Proposition A1 given below. For this purpose, we require a few properties of log-moment generating function, which we briefly review next.

**Lemma A1**.
*(Theorem 15.3, Theorem 15.6 in [43])*

(i) *$\psi_{P_{Z},f}\left(0\right)=0$ and $\psi_{P_{Z},f}^{\prime }\left(0\right)=E_{P_{Z}}\left[f\left(Z\right)\right]$, where $\psi_{P_{Z},f}^{\prime }\left(\lambda \right)$ denotes the derivative of $\psi_{P_{Z},f}\left(\lambda \right)$ with respect to λ.*
(ii) *$\psi_{P_{Z},f}\left(\lambda \right)$ is a strictly convex function in λ.*
(iii) *$\psi_{P_{Z},f}^{*}\left(\theta \right)$ is strictly convex and strictly positive in θ except $\psi_{P_{Z},f}^{*}\left(E_{P_{Z}}\left[Z\right]\right)=0$.*

Proposition A1 is basically a characterization of the error-exponent region of a hypothesis testing problem, which we introduce next. Let $P_{X_{0}X_{1}}\in P\left(X^{2}\right)$ be an arbitrary joint PMF, and consider a sequence of pairs of *n*-length sequences $\left(\tilde{x},x^{\prime }\right)$ such that

(A1) $$\begin{array}{c} P_{\tilde{x}x^{\prime }}\left(\tilde{x},x^{\prime }\right)\overset{\left(n\right)}{\to }P_{X_{0}X_{1}}\left(\tilde{x},x^{\prime }\right),\;\forall \;\left(\tilde{x},x^{\prime }\right)\in X^{2}. \end{array}$$

Consider the following HT:

(A2a) $$H_{0}:Y∼P_{Y|X}^{⊗n}\left(\cdot |\tilde{x}\right),$$

(A2b) $$H_{1}:Y∼P_{Y|X}^{⊗n}\left(\cdot |x^{\prime }\right).$$

With the achievability of an error-exponent pair $\left(\kappa_{\alpha },\kappa_{\beta }\right)$ defined similar to Definition 1, consider the error-exponent region of interest

$$\begin{array}{cc} \; & R^{\prime }\left(P_{X_{0}X_{1}}\right):=\left\{\left(\kappa_{\alpha },\kappa^{\prime }\left(\kappa_{\alpha },P_{X_{0}X_{1}}\right)\right):\kappa_{\alpha }\in \left(0,\kappa_{\alpha }^{\prime ★}\right)\right\}, \end{array}$$

where $\kappa^{\prime }\left(\kappa_{\alpha },P_{X_{0}X_{1}}\right):=\sup\left\{\kappa_{\beta }:\left(\kappa_{\alpha },\kappa_{\beta }\right)\mathrm{is}\;\mathrm{achievable}\;\mathrm{for}\;\mathrm{HT in}\;\mathrm{in}\;(\mathrm{A2})\right\}$ and $\kappa_{\alpha }^{\prime ★}:=\inf\left\{\kappa_{\alpha }:\kappa^{\prime }\left(\kappa_{\alpha },P_{X_{0}X_{1}}\right)=0\right\}$. We mention at this point that the notation $R^{\prime }\left(P_{X_{0}X_{1}}\right)$ is justified as the error-exponent region for the above hypothesis test depends on $\left(\tilde{x},x^{\prime }\right)$ only through its limiting joint type $P_{X_{0}X_{1}}$, as will be evident later. Given this, the following proposition provides a single-letter characterization of $R^{\prime }\left(P_{X_{0}X_{1}}\right)$.

**Proposition A1**.

$$\begin{array}{cc} \; & R^{\prime }\left(P_{X_{0}X_{1}}\right)=\underset{\theta \in I(P_{X_{0}X_{1}})}{\cup }\left(E_{P_{X_{0}X_{1}}}\left[\psi_{P_{Y|X}\left(\cdot |X_{0}\right),\Pi_{X_{0},X_{1}}}^{*}\left(\theta \right)\right],\;E_{P_{X_{0}X_{1}}}\left[\psi_{P_{Y|X}\left(\cdot |X_{0}\right),\Pi_{X_{0},X_{1}}}^{*}\left(\theta \right)\right]-\theta \right) \end{array}$$

*where $\Pi_{\tilde{x},x^{\prime }}\left(y\right):=\log\left(P_{Y|X}\left(y|x^{\prime }\right)/P_{Y|X}\left(y|\tilde{x}\right)\right)$ for $\left(\tilde{x},x^{\prime }\right)\in X^{2}$,*

$$\begin{array}{cc} I(P_{X_{0}X_{1}}) & :=\left(-d_{\min}\left(P_{X_{0}X_{1}}\right),d_{\max}\left(P_{X_{0}X_{1}}\right)\right),\\ d_{\min}\left(P_{X_{0}X_{1}}\right) & :=E_{P_{X_{0}X_{1}}}\left[D\left(P_{Y|X}\left(\cdot |X_{0}\right)\left|\right|P_{Y|X}\left(\cdot |X_{1}\right)\right)\right]\\ d_{\max}\left(P_{X_{0}X_{1}}\right) & :=E_{P_{X_{0}X_{1}}}\left[D\left(P_{Y|X}\left(\cdot |X_{1}\right)\left|\right|P_{Y|X}\left(\cdot |X_{0}\right)\right)\right]. \end{array}$$

**Proof**. Let $\left(\tilde{x},x^{\prime }\right)\in X^{n}\times X^{n}$ be sequences that satisfy (A1). For simplicity, we will denote $d_{\max}\left(P_{X_{0}X_{1}}\right)$ and $d_{\min}\left(P_{X_{0}X_{1}}\right)$ by $d_{\max}$ and $d_{\min}$, respectively.

**Achievability:** We will show that for $-d_{\min}<\theta <d_{\max}$,

$$\begin{array}{c} \kappa^{\prime }\left(E_{P_{X_{0}X_{1}}}\left[\psi_{P_{Y|X}\left(\cdot |X_{0}\right),\Pi_{X_{0},X_{1}}}^{*}\left(\theta \right)\right],P_{X_{0}X_{1}}\right)\geq E_{P_{X_{0}X_{1}}}\left[\psi_{P_{Y|X}\left(\cdot |X_{0}\right),\Pi_{X_{0},X_{1}}}^{*}\left(\theta \right)\right]-\theta . \end{array}$$

Consider the Neyman–Pearson test given by $g_{n}\left(y\right)={𝟙}_{\left\{\Pi_{\tilde{x},x^{\prime }}^{\left(n\right)}\left(y\right)\geq n\theta \right\}}$, where $\Pi_{\tilde{x},x^{\prime }}^{\left(n\right)}\left(y\right):=\sum_{i=1}^{n}\Pi_{{\tilde{x}}_{i},x_{i}^{\prime },P_{Y|X}}\left(y_{i}\right)$. Observe that the type I error probability can be upper bounded for $\theta >-d_{\min}$ and sufficiently large *n* as follows:

(A3) $$\begin{array}{ccc} \alpha_{n}\left(g_{n}\right) & =P_{P_{Y|X}^{⊗n}\left(\cdot |\tilde{x}\right)}\left(\Pi_{\tilde{x},x^{\prime }}^{\left(n\right)}\left(Y\right)\geq n\theta \right)\\ & \overset{\left(a\right)}{\leq }e^{-\underset{\lambda \geq 0}{\sup}\;n\theta \lambda -\psi_{P_{Y|X}^{⊗n}\left(\cdot |\tilde{x}\right),\Pi_{\tilde{x},x^{\prime }}^{\left(n\right)}}\left(\lambda \right)}\\ & \overset{\left(b\right)}{=}e^{-\underset{\lambda \in R}{\sup}\;n\left(\theta \lambda -\frac{1}{n}\psi_{P_{Y|X}^{⊗n}\left(\cdot |\tilde{x}\right),\Pi_{\tilde{x},x^{\prime }}^{\left(n\right)}}\left(\lambda \right)\right)}, & \end{array}$$

where (a) follows from the Chernoff bound, and (b) holds because for $\theta >-d_{\min}$ and sufficiently large *n*, the supremum in (A3) always occurs at λ≥0. To see this, note that the term $l_{n}\left(\lambda \right):=\theta \lambda -n^{-1}\psi_{P_{Y|X}^{⊗n}\left(\cdot |\tilde{x}\right),\Pi_{\tilde{x},x^{\prime }}^{\left(n\right)}}\left(\lambda \right)$ is a concave function of λ by Lemma A1 (i). Additionally, denoting its derivative with respect to λ by $l_{n}^{\prime }\left(\lambda \right)$, we have

(A4) $$\begin{array}{cc} l_{n}^{\prime }\left(0\right) & =\theta -\frac{1}{n}\;E_{P_{Y|X}^{⊗n}\left(\cdot |\tilde{x}\right)}\left[\Pi_{\tilde{x},x^{\prime }}^{\left(n\right)}\right]\\ & =\theta -\frac{1}{n}\sum_{i=1}^{n}E_{P_{Y|X}\left(\cdot |{\tilde{x}}_{i}\right)}\left[\log\left(P_{Y|X}\left(Y_{i}|x_{i}^{\prime }\right)/P_{Y|X}\left(Y_{i}|{\tilde{x}}_{i}\right)\right)\right] \end{array}$$

(A5) $$\begin{array}{ccc} & \overset{\left(n\right)}{\to }\theta +d_{\min}>0, & \end{array}$$

where (A4) follows from Lemma A1 (iii), and (A5) is due to the absolute continuity assumption, $P_{Y|X}\left(\cdot |x\right)\ll P_{Y|X}\left(\cdot |x^{\prime }\right)$, $\forall \;\left(x,x^{\prime }\right)\in X^{2}$ on the channel, and (A1). Thus, by the concavity of $l_{n}\left(\lambda \right)$, its supremum has to occur at λ≥0. Simplifying the term within the exponent in (A3), we obtain

(A6) $$\begin{array}{cc} \frac{1}{n}\psi_{P_{Y|X}^{⊗n}\left(\cdot |x\right),\Pi_{\tilde{x},x^{\prime }}^{\left(n\right)}}\left(\lambda \right) & =\sum_{\tilde{x},x^{\prime }}P_{\tilde{x}x^{\prime }}\left(\tilde{x},x^{\prime }\right)\log\left(E_{P_{Y|X}\left(\cdot |\tilde{x}\right)}\left[{\left(P_{Y|X}\left(Y|x^{\prime }\right)/P_{Y|X}\left(Y|\tilde{x}\right)\right)}^{\lambda }\right]\right) \end{array}$$

(A7) $$\begin{array}{ccc} & \overset{\left(n\right)}{\to }E_{P_{X_{0}X_{1}}}\left[\log\left(E_{P_{Y|X}\left(\cdot |X_{0}\right)}\left[e^{\lambda \Pi_{X_{0},X_{1}}\left(Y\right)}\right]\right)\right], & \end{array}$$

where (A7) follows from (A1) and the absolute continuity assumption on $P_{Y|X}$. Substituting (A7) in (A3) and from (1), we obtain for arbitrarily small (but fixed) δ>0 and sufficiently large *n*, that

(A8) $$\begin{array}{ccc} \alpha_{n}\left(g_{n}\right) & \leq e^{-\underset{\lambda \in R}{\sup}\left(n\left(\theta \lambda -E_{P_{X_{0}X_{1}}}\left[\log\left(E_{P_{Y|X}\left(\cdot |X_{0}\right)}\left[e^{\lambda \Pi_{X_{0},X_{1}}\left(Y\right)}\right]\right)\right]-\delta \right)\right)}\\ & =e^{-n\left(E_{P_{X_{0}X_{1}}}\left[\underset{\lambda \in R}{\sup}\left(\theta \lambda -E_{P_{Y|X}\left(\cdot |X_{0}\right)}\left(e^{\lambda \Pi_{X_{0},X_{1}}\left(Y\right)}\right)\right)\right]-\delta \right)}\\ & =e^{-n\left(E_{P_{X_{0}X_{1}}}\left[\psi_{P_{Y|X}\left(\cdot |X_{0}\right),\Pi_{X_{0},X_{1}}}^{*}\left(\theta \right)\right]-\delta \right)}. & \end{array}$$

Similarly, it can be shown that for $\theta <d_{\max}$,

(A9) $$\begin{array}{cc} \beta_{n}\left(g_{n}\right) & \leq e^{-n\left(E_{P_{X_{0}X_{1}}}\left[\psi_{P_{Y|X}\left(\cdot |X_{1}\right),\Pi_{X_{0},X_{1}}}^{*}\left(\theta \right)\right]-\delta \right)}. \end{array}$$

Moreover, for $\left(\tilde{x},x^{\prime }\right)\in X^{2}$, we have

$$\begin{array}{cc} e^{\psi_{P_{Y|X}\left(\cdot |x^{\prime }\right),\Pi_{\tilde{x},x^{\prime }}}\left(\lambda \right)} & =\sum_{y\in Y}P_{Y|X}^{\lambda +1}\left(\cdot |x^{\prime }\right)/P_{Y|X}^{\lambda }\left(\cdot |\tilde{x}\right)=e^{\psi_{P_{Y|X}\left(\cdot |\tilde{x}\right),\Pi_{\tilde{x},x^{\prime }}}\left(\lambda +1\right)}. \end{array}$$

It follows that

$$\begin{array}{ccc} \psi_{P_{Y|X}\left(\cdot |x^{\prime }\right),\Pi_{\tilde{x},x^{\prime }}}^{*}\left(\theta \right) & :=\underset{\lambda \in R}{\sup}\;\lambda \theta -\psi_{P_{Y|X}\left(\cdot |x^{\prime }\right),\Pi_{\tilde{x},x^{\prime }}}\left(\lambda \right)\\ & =\underset{\lambda \in R}{\sup}\;\lambda \theta -\psi_{P_{Y|X}\left(\cdot |\tilde{x}\right),\Pi_{\tilde{x},x^{\prime }}}\left(\lambda +1\right)\\ & =\psi_{P_{Y|X}\left(\cdot |\tilde{x}\right),\Pi_{\tilde{x},x^{\prime }}}^{*}\left(\theta \right)-\theta . & \end{array}$$

Hence,

$$\begin{array}{c} E_{P_{X_{0}X_{1}}}\left[\psi_{P_{Y|X}\left(\cdot |X_{1}\right),\Pi_{X_{0},X_{1}}}^{*}\left(\theta \right)\right]=E_{P_{X_{0}X_{1}}}\left[\psi_{P_{Y|X}\left(\cdot |X_{0}\right),\Pi_{X_{0},X_{1}}}^{*}\left(\theta \right)\right]-\theta . \end{array}$$

From this, (A8) and (A9), we obtain for $-d_{\min}<\theta <d_{\max}$ that

$$\begin{array}{c} \kappa^{\prime }\left(E_{P_{X_{0}X_{1}}}\left[\psi_{P_{Y|X}\left(\cdot |X_{0}\right),\Pi_{X_{0},X_{1}}}^{*}\left(\theta \right)\right]-\delta ,P_{X_{0}X_{1}}\right)\geq E_{P_{X_{0}X_{1}}}\left[\psi_{P_{Y|X}\left(\cdot |X_{0}\right),\Pi_{X_{0},X_{1}}}^{*}\left(\theta \right)\right]-\theta -\delta . \end{array}$$

Then, the proof of achievability is completed by noting that δ>0 is arbitrary and $\kappa^{\prime }\left(\kappa_{\alpha },P_{X_{0}X_{1}}\right)$ is a continuous function of $\kappa_{\alpha }$ for a fixed $P_{X_{0}X_{1}}$.

**Converse:** Let $I_{n}\left(\tilde{x},x^{\prime }\right):=\left\{i\in \left[n\right]:{\tilde{x}}_{i}=\tilde{x}\;\mathrm{and}\;x_{i}^{\prime }=x^{\prime }\right\}$. For any θ∈R and decision function $g_{n}$, we have from Theorem 14.9 in [43] that
  $$\begin{array}{cc} \alpha_{n}\left(g_{n}\right)+e^{-n\theta }\beta_{n}\left(g_{n}\right) & \geq P_{P_{Y|X}^{⊗n}\left(\cdot |\tilde{x}\right)}\left(\log\left(P_{Y|X}^{⊗n}\left(Y|x^{\prime }\right)/P_{Y|X}^{⊗n}\left(Y|\tilde{x}\right)\right)\geq n\theta \right). \end{array}$$

Simplifying the RHS above, we obtain

$$\begin{array}{ccc} & P_{P_{Y|X}^{⊗n}\left(\cdot |\tilde{x}\right)}\left(\log\left(P_{Y|X}^{⊗n}\left(Y|x^{\prime }\right)/P_{Y|X}^{⊗n}\left(Y|\tilde{x}\right)\right)\geq n\theta \right)\\ & =P_{P_{Y|X}^{⊗n}\left(\cdot |\tilde{x}\right)}\left(\sum_{\tilde{x},x^{\prime }}\sum_{i\in I_{n}\left(\tilde{x},x^{\prime }\right)}\log\left(P_{Y|X}\left(Y_{i}|x_{i}^{\prime }\right)/P_{Y|X}\left(Y_{i}|{\tilde{x}}_{i}\right)\right)\geq n\theta \right)\\ & =P_{P_{Y|X}^{⊗n}\left(\cdot |\tilde{x}\right)}\left(\sum_{\tilde{x},x^{\prime }}\sum_{i\in I_{n}\left(\tilde{x},x^{\prime }\right)}\log\left(P_{Y|X}\left(Y_{i}|x_{i}^{\prime }\right)/P_{Y|X}\left(Y_{i}|{\tilde{x}}_{i}\right)\right)\geq \sum_{\left(\tilde{x},x^{\prime }\right)\in X^{2}}nP_{\tilde{x}x^{\prime }}\left(\tilde{x},x^{\prime }\right)\theta \right)\\ & \overset{\left(a\right)}{\geq }P_{P_{Y|X}^{⊗n}\left(\cdot |\tilde{x}\right)}\left(\underset{\tilde{x},x^{\prime }}{⋂}\left(\sum_{i\in I_{n}\left(\tilde{x},x^{\prime }\right)}\log\left(P_{Y|X}\left(Y_{i}|x_{i}^{\prime }\right)/P_{Y|X}\left(Y_{i}|{\tilde{x}}_{i}\right)\right)\geq nP_{\tilde{x}x^{\prime }}\left(\tilde{x},x^{\prime }\right)\theta \right)\right)\\ & \overset{\left(b\right)}{=}\prod_{\left(\tilde{x},x^{\prime }\right)\in X^{2}}P_{P_{Y|X}^{⊗n}\left(\cdot |\tilde{x}\right)}\left(\sum_{i\in I_{n}\left(\tilde{x},x^{\prime }\right)}\log\left(P_{Y|X}\left(Y_{i}|x_{i}^{\prime }\right)/P_{Y|X}\left(Y_{i}|{\tilde{x}}_{i}\right)\right)\geq nP_{\tilde{x}x^{\prime }}\left(\tilde{x},x^{\prime }\right)\theta \right), & \end{array}$$

where

(a) follows since
  $$\begin{array}{cc} \; & \underset{\tilde{x},x^{\prime }}{⋂}\left\{\sum_{i\in I_{n}\left(\tilde{x},x^{\prime }\right)}\log\left(P_{Y|X}\left(Y_{i}|x_{i}^{\prime }\right)/P_{Y|X}\left(Y_{i}|{\tilde{x}}_{i}\right)\right)\geq nP_{\tilde{x}x^{\prime }}\left(\tilde{x},x^{\prime }\right)\theta \right\}\\ \; & \;\subseteq \left\{\sum_{\tilde{x},x^{\prime }}\sum_{i\in I_{n}\left(\tilde{x},x^{\prime }\right)}\log\left(P_{Y|X}\left(Y_{i}|x_{i}^{\prime }\right)/P_{Y|X}\left(Y_{i}|{\tilde{x}}_{i}\right)\right)\geq \sum_{\left(\tilde{x},x^{\prime }\right)\in X^{2}}nP_{\tilde{x}x^{\prime }}\left(\tilde{x},x^{\prime }\right)\theta \right\}; \end{array}$$
(b) is due to the independence of the events $\left\{\sum_{i\in I_{n}\left(\tilde{x},x^{\prime }\right)}\log\left(P_{Y|X}\left(Y_{i}|x_{i}^{\prime }\right)/P_{Y|X}\left(Y_{i}|{\tilde{x}}_{i}\right)\right)\geq nP_{\tilde{x}x^{\prime }}\left(\tilde{x},x^{\prime }\right)\theta \right\}$ for different $\left(\tilde{x},x^{\prime }\right)\in X^{2}$.

Define $b_{\tilde{x},x^{\prime }}\left(\theta \right):=\min_{\begin{array}{c} {\tilde{Q}}_{\tilde{x}}\in P\left(Y\right):E_{{\tilde{Q}}_{\tilde{x}}}\left[\log\left(P_{Y|X}\left(Y|x^{\prime }\right)/P_{Y|X}\left(Y|\tilde{x}\right)\right)\right]\geq \theta \end{array}}\;D\left({\tilde{Q}}_{x}\left|\right|P_{Y|X}\left(\cdot |\tilde{x}\right)\right)$. Then, for arbitrary δ>0, $\delta^{\prime }>\delta$ and sufficiently large *n*, we can write

$$\begin{array}{ccc} \alpha_{n}+e^{-n\theta }\beta_{n} & \overset{\left(a\right)}{\geq }\prod_{\left(\tilde{x},x^{\prime }\right)\in X^{2}}e^{-nP_{\tilde{x}x^{\prime }}\left(\tilde{x},x^{\prime }\right)\;\left(b_{\tilde{x},x^{\prime }}\left(\theta \right)+\delta \right)}\\ & \overset{\left(b\right)}{\geq }\prod_{\left(\tilde{x},x^{\prime }\right)\in X^{2}}e^{-nP_{\tilde{x}x^{\prime }}\left(\tilde{x},x^{\prime }\right)\left(\psi_{P_{Y|X}\left(\cdot |\tilde{x}\right),\Pi_{\tilde{x},x^{\prime }}}^{*}\left(\theta \right)+\delta \right)}\\ & \overset{\left(c\right)}{=}e^{-n\left(E_{P_{X_{0}X_{1}}}\left[\psi_{P_{Y|X}\left(\cdot |X_{0}\right),\Pi_{X_{0},X_{1}}}^{*}\left(\theta \right)\right]+\delta^{\prime }\right)}, & \end{array}$$

where (a) follows from Theorem 15.9 in [43]; (b) follows since $b_{\tilde{x},x^{\prime }}\left(\theta \right)=\psi_{P_{Y|X}\left(\cdot |\tilde{x}\right),\Pi_{\tilde{x},x^{\prime }}}^{*}\left(\theta \right)$ from Theorem 15.6 in [43] and Theorem 15.11 in [43]; and (c) is due to (A1). The equation above implies that

$$\begin{array}{c} \underset{n\to \infty }{lim sup}\min\left\{-\frac{1}{n}\log\alpha_{n},-\frac{1}{n}\log\beta_{n}+\theta \right\}\leq E_{P_{X_{0}X_{1}}}\left[\psi_{P_{Y|X}\left(\cdot |X_{0}\right),\Pi_{X_{0},X_{1}}}^{*}\left(\theta \right)\right]+\delta^{\prime }. \end{array}$$

Hence, if $-\log\left(\alpha_{n}\right)/n>E_{P_{X_{0}X_{1}}}\left[\psi_{P_{Y|X}\left(\cdot |X_{0}\right),\Pi_{X_{0},X_{1}}}^{*}\left(\theta \right)\right]+\delta^{\prime }$ for all sufficiently large *n*, then

$$\begin{array}{c} \underset{n\to \infty }{lim sup}-\frac{1}{n}\log\beta_{n}\leq E_{P_{X_{0}X_{1}}}\left[\psi_{P_{Y|X}\left(\cdot |X_{0}\right),\Pi_{X_{0},X_{1}}}^{*}\left(\theta \right)\right]-\theta +\delta^{\prime }. \end{array}$$

Since δ (and $\delta^{\prime }$) is arbitrary, this implies via the continuity of $\kappa^{\prime }\left(\kappa_{\alpha },P_{X_{0}X_{1}}\right)$ in $\kappa_{\alpha }$ that

$$\begin{array}{c} \kappa^{\prime }\left(E_{P_{X_{0}X_{1}}}\left[\psi_{P_{Y|X}\left(\cdot |X_{0}\right),\Pi_{X_{0},X_{1}}}^{*}\left(\theta \right)\right],P_{X_{0}X_{1}}\right)\leq E_{P_{X_{0}X_{1}}}\left[\psi_{P_{Y|X}\left(\cdot |X_{0}\right),\Pi_{X_{0},X_{1}}}^{*}\left(\theta \right)\right]-\theta . \end{array}$$

To complete the proof, we need to show that θ can be restricted to lie in $I(P_{X_{0}X_{1}})$. Toward this, it suffices to show the following:

(i) $E_{P_{X_{0}X_{1}}}\left[\psi_{P_{Y|X}\left(\cdot |X_{0}\right),\Pi_{X_{0},X_{1}}}^{*}\left(-d_{\min}\right)\right]=0$,
(ii) $E_{P_{X_{0}X_{1}}}\left[\psi_{P_{Y|X}\left(\cdot |X_{0}\right),\Pi_{X_{0},X_{1}}}^{*}\left(d_{\max}\right)\right]=d_{\max}$, and
(iii) $E_{P_{X_{0}X_{1}}}\left[\psi_{P_{Y|X}\left(\cdot |X_{0}\right),\Pi_{X_{0},X_{1}}}^{*}\left(\theta \right)\right]$ and $E_{P_{X_{0}X_{1}}}\left[\psi_{P_{Y|X}\left(\cdot |X_{0}\right),\Pi_{X_{0},X_{1}}}^{*}\left(\theta \right)\right]-\theta$ are convex functions of θ.

We have

(A10) $$\begin{array}{cc} & E_{P_{X_{0}X_{1}}}\left[\psi_{P_{Y|X}\left(\cdot |X_{0}\right),\Pi_{X_{0},X_{1}}}^{*}\left(-d_{\min}\right)\right]\\ & :=\underset{\lambda \in R}{\sup}-\lambda \;E_{P_{X_{0}X_{1}}}\left[D\left(P_{Y|X}\left(\cdot |X_{0}\right)\left|\right|P_{Y|X}\left(\cdot |X_{1}\right)\right)\right]-E_{P_{X_{0}X_{1}}}\left[\psi_{P_{Y|X}\left(\cdot |X_{0}\right),\Pi_{X_{0},X_{1}}}\left(\lambda \right)\right]\\ & \leq \sum_{\tilde{x},x^{\prime }}P_{X_{0}X_{1}}\left(\tilde{x},x^{\prime }\right)\;\left[\underset{\lambda_{\tilde{x},x^{\prime }}\in R}{\sup}-\lambda_{\tilde{x},x^{\prime }}D\left(P_{Y|X}\left(\cdot |\tilde{x}\right)\left|\right|P_{Y|X}\left(\cdot |x^{\prime }\right)\right)-\psi_{P_{Y|X}\left(\cdot |\tilde{x}\right),\Pi_{\tilde{x},x^{\prime }}}\left(\lambda_{\tilde{x},x^{\prime }}\right)\right]\\ & =0, \end{array}$$

where (A10) follows since each term inside the square braces in the penultimate equation is zero, which in turn follows from Lemma A1 (iii). Additionally,

(A11) $$\begin{array}{ccc} E_{P_{X_{0}X_{1}}}\left[\psi_{P_{Y|X}\left(\cdot |X_{0}\right),\Pi_{X_{0},X_{1}}}^{*}\left(-d_{\min}\right)\right] & =\sum_{\tilde{x},x^{\prime }}P_{X_{0}X_{1}}\left(\tilde{x},x^{\prime }\right)\;\psi_{P_{Y|X}\left(\cdot |\tilde{x}\right),\Pi_{\tilde{x},x^{\prime }}}^{*}\left(-d_{\min}\right)\geq 0, & \end{array}$$

where (A11) follows from the non-negativity of $\psi_{P_{Y|X}\left(\cdot |\tilde{x}\right),\Pi_{\tilde{x},x^{\prime }}}^{*}$ for every $\left(\tilde{x},x^{\prime }\right)\in X^{2}$ stated in Lemma A1 (iii). Combining (A10) and (A11) proves (i). We also have

(A12) $$\begin{array}{ccc} E_{P_{X_{0}X_{1}}}\left[\psi_{P_{Y|X}\left(\cdot |X_{0}\right),\Pi_{X_{0},X_{1}}}^{*}\left(d_{\max}\right)\right]-d_{\max} & =E_{P_{X_{0}X_{1}}}\left[\psi_{P_{Y|X}\left(\cdot |X_{1}\right),\Pi_{X_{0},X_{1}}}^{*}\left(d_{\max}\right)\right]=0, & \end{array}$$

where the final equality follows similarly to the proof of (i). This proves (ii). Finally, (iii) follows from Lemma A1 (iii) and the fact that a weighted sum of convex functions is convex provided the weights are non-negative, thus completing the proof. □
